# SCF/c-kit transactivates CXCR4-serine 339 phosphorylation through G protein-coupled receptor kinase 6 and regulates cardiac stem cell migration

**DOI:** 10.1038/srep26812

**Published:** 2016-06-01

**Authors:** Ke Zuo, Dong Kuang, Ying Wang, Yanli Xia, Weilin Tong, Xiaoyan Wang, Yaobin Chen, Yaqi Duan, Guoping Wang

**Affiliations:** 1 Institute of Pathology, Tongji Hospital, Tongji Medical College, Huazhong University of Science and Technology, Wuhan 430030, P. R. of China; 2 Department of Pathology, School of Basic Medicine, Tongji Medical College, Huazhong University of Science and Technology, Wuhan 430030, P. R. of China

## Abstract

C-kit positive cardiac stem cells (CSCs) have been shown to contribute to myocardial regeneration after infarction. Previously, we have shown that the c-kit ligand stem cell factor (SCF) can induce CSC migration into the infarcted area during myocardial infarction (MI). However, the precise mechanism involved is not fully understood. In this study, we found that CSCs also express C-X-C chemokine receptor type 4 (CXCR4), which is a typical member of the seven transmembrane-spanning G protein-coupled receptor (GPCR). *In vitro*, activation of c-kit signalling by SCF promotes migration of CSCs with increased phosphorylation of CXCR4-serine 339, p38 mitogen-activated protein kinase (p38 MAPK) and extracellular regulated protein kinases 1/2 (ERK1/2). Knockdown of CXCR4 expression by siRNA reduces SCF/c-kit-induced migration and downstream signalling. As previously reported, CXCR4-serine 339 phosphorylation is mainly regulated by GPCR kinase 6 (GRK6); thus, silencing of GRK6 expression by siRNA impairs CXCR4-serine 339 phosphorylation and migration of CSCs caused by SCF. *In vivo*, knockdown of GRK6 impairs the ability of CSCs to migrate into peri-infarcted areas. These results demonstrate that SCF-induced CSC migration is regulated by the transactivation of CXCR4-serine 339 phosphorylation, which is mediated by GRK6.

In the mammalian heart, c-kit-positive cardiac stem cells (CSCs) are clustered in interstitial niches and contribute to myocardial homeostasis and tissue repair following injury^1^,^2^,^3^,^4^,^5^. Stem cell factor (SCF), also called the c-kit ligand, is a critical factor for the development, proliferation and migration of CSCs^6^,^7^. After myocardial infarction (MI), SCF levels can be significantly increased in the left ventricle myocardium, especially in peri-infarcted areas^6^. Because of the chemoattractive effect of SCF, CSCs can migrate from remote areas into peri-infarcted areas. According to our previous work^6^, c-kit, as well as its downstream signalling partner p38 mitogen-activated protein kinase (p38 MAPK), is activated after SCF stimulation. However, inhibition of p38 MAPK cannot fully inhibit SCF-induced CSC migration. Consequently, although p38 MAPK is involved in the migration process, this mechanism cannot solely explain the migration of CSCs.

C-X-C chemokine receptor type 4 (CXCR4) is a typical member of the seven transmembrane-spanning G protein-coupled receptor (GPCR) and is also expressed in CSCs^8^, which can be activated by agonist stimulation, i.e., the stromal cell-derived factor 1 (SDF-1). Activation of CXCR4 by SDF-1 has been implicated in the homeostasis and activation of the immune system and influences a range of biological systems under both normal and pathological conditions^9^,^10^,^11^,^12^. These include angiogenesis, cell survival, cell mobilization and migration, tumour growth and metastasis, and so forth^9^,^10^,^11^. Classically, after SDF-1 treatment, CXCR4 is rapidly phosphorylated with different serine residues in the intracellular C-terminal domain^13^,^14^. This process is predominantly mediated by members of the GPCR kinase (GRK) family, which specifically phosphorylates agonist-occupied GPCRs^15^,^16^. Recently, CXCR4-serine 339 phosphorylation has been inferred to play an important role in tumour cell migration and metastasis and be regarded as a marker for poor prognosis in acute myeloid leukaemia^12^,^17^,^18^. Despite the fact that CXCR4 is expressed in a wide range of tissues and organs, the role of CXCR4, including characterization of signal transduction mechanisms in CSCs, is less well established.

It has been well documented that cross-talk between GPCRs and growth factor receptor tyrosine kinases (RTKs) exists in different cellular systems. For example, C-C motif chemokine 11 (CCL11), a ligand for the GPCR C-C chemokine receptor type 3 (CCR3), could induce EGFR(epidermal growth factor receptor) tyrosine phosphorylation in bronchial epithelial cells^19^. In rat aortic vascular smooth muscle cells, both platelet-derived growth factor receptors (PDGFR) and EGFR are phosphorylated in response to sphingosine 1-phosphate (S1P), which is a ligand for the S1PR family of GPCRs^20^, and stimulation of β1-adrenergic receptors also induces EGFR transactivation and contributes to cardioprotection^21^. Moreover, CXCR4-mediated mobilization is modulated by c-kit activity in bone marrow progenitor cells^22^. In contrast, examples of transactivation of GPCRs by RTKs are less abundant.

Here, we have examined the potential cross-talk in the signal transduction pathways between CXCR4 and c-kit and identified a new mechanism that CXCR4-serine 339 transactivation by SCF/c-kit signalling is necessary for CSC migration.

## Results

### CSC generation and phenotypic characterization

**Figure 1:**
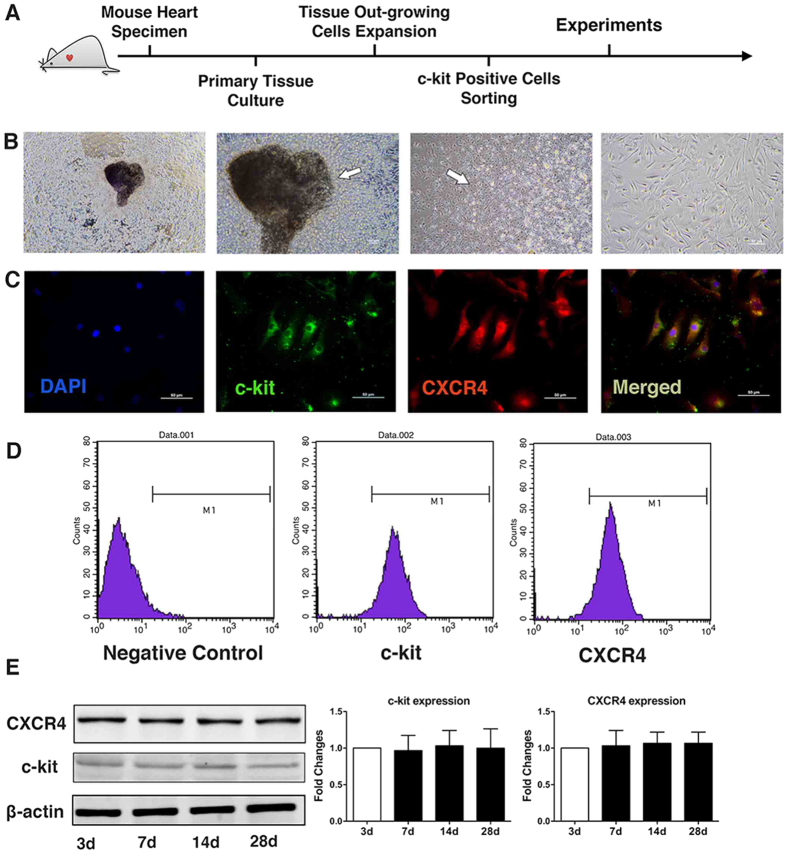
Isolation, expansion, and phenotypic characterization of CSCs. (**A**) Schematic depiction of the steps involved from tissue harvest to immunomagnetic sorting for c-kit(+) cells. (**B**) Heart tissue explant (a) and phase-bright cells migrated from the explant (b). CSCs presented clone-like proliferation and newly formed cells (arrow) migrate away from the centre (c). After cell sorting, c-kit(+) cells present with heterogeneous morphology (d). (**C**) CPCs stained for c-kit and CXCR4 and counterstained with the DNA dye DAPI to reveal nuclei. (**D**) Flow cytometric analyses of CSCs for expression of the cell surface markers c-kit and CXCR4. (**E**) During different time points of cell culture, the expression of c-kit and CXCR4 was detected using western blotting (WB) (normalized to beta-actin, three independent experiments). Error bars represent SEM. Full immunoblots for the cropped images presented here are provided in the Supplementary Information.

CSCs were obtained upon mild enzymatic digestion of adult C57BL/6 mouse hearts, and c-kit(+) cells were enriched using magnetically activated cell sorting (Fig. 1A). After approximately two weeks in culture, a layer of fibroblast-like cells emerged from adherent mouse cardiac explants followed by the migration of small, round, phase-bright cells (Fig. 1B). Inverted phase-contrast microscope examinations showed that CSCs presented clone-like proliferation (Fig. 1B). Cell surface marker expression was analysed by immunocytochemistry staining of c-kit and CXCR4 (Fig. 1C). The purity of sorted CSCs was characterized by flow cytometric analyses of the cell surface marker c-kit and CXCR4 (Fig. 1D). More than 85% of cells were positive for both c-kit and CXCR4. At different time points during cell culture, the expression of c-kit and CXCR4 were found to be maintained well as detected by immunoblotting (Fig. 1E).

### SCF induces CSC migration and activates MAPK signalling

**Figure 2:**
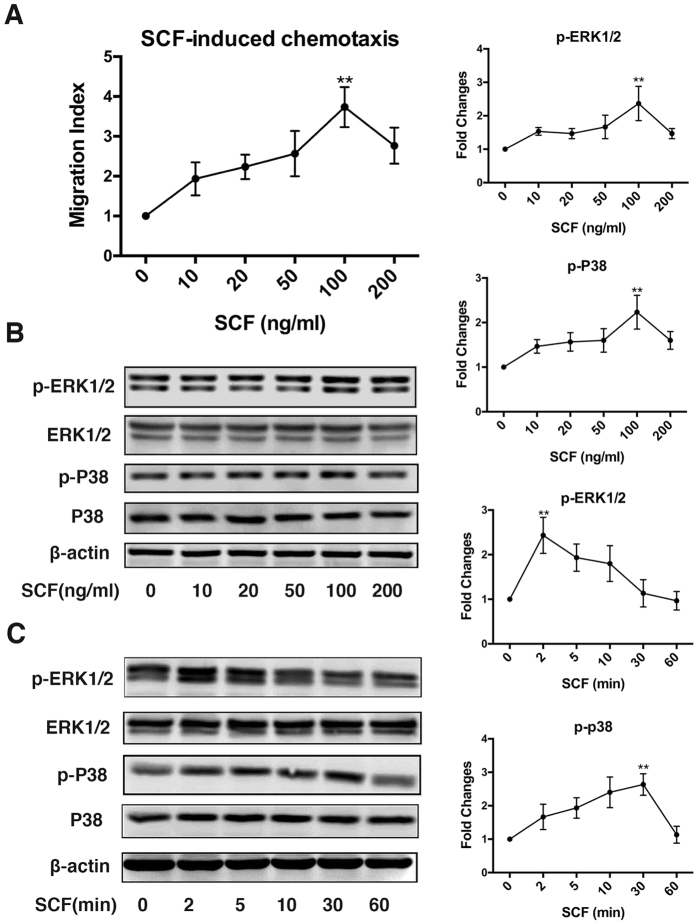
SCF induces CSC migration and activates MAPK signalling. (**A**) SCF induced CSC migration in a dose-dependent manner (three independent experiments). (**B**,**C**) SCF caused p38 MAPK and ERK1/2 phosphorylation in a dose- and time-dependent manner, respectively. The line graphs represent the quantification analysis of p-p38 MAPK and p-ERK1/2 (normalized to unphosphorylated state, three independent experiments). **p < 0.01 vs control group (0 ng/ml or 0 min). Full immunoblots for the cropped images presented here are provided in the Supplementary Information.

To assess the chemotaxis of SCF on CSCs, we used the Transwell migration assay to quantitatively evaluate CSC migration *in vitro*. In the lower chamber was placed medium (DMEM/F12) alone or containing different concentrations of SCF (0, 10, 20, 50, 100, or 200 ng/ml), and CSCs were placed in the upper chamber. After 12 h of culture, the average number of migrated CSCs increased significantly compared with the control group as the concentration of SCF increased, which reached a peak at 100 ng/ml (Fig. 2A).

It has been previously reported that p38 MAPK and ERK1/2 are downstream of the SCF/c-Kit pathway; thus, dose- and time-dependent assays were performed to analyse the effect of SCF on p38 MAPK and ERK1/2 phosphorylation. As shown in Fig. 2B, the phosphorylation of p38 MAPK and ERk1/2 both peaked in a dose-dependent manner at 100 ng/ml of SCF. In analysing time dependence, phospho-p38 MAPK and phospho-ERK1/2 increased significantly after 2 min of SCF stimulation and returned to basal levels 60 min after the application of SCF (Fig. 2C).

### CXCR4 is involved in SCF induced CSC migration and MAPK activation

**Figure 3:**
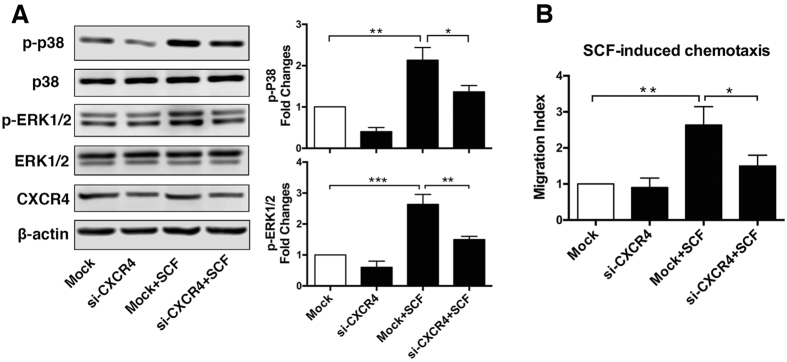
CXCR4 is involved in SCF induced CSC migration and MAPK activation. (**A**) Left panel is a representative western blot (WB) demonstrating specific and efficient knockdown of CXCR4 endogenously expressed in CSCs 48 h post-transfection. With SCF (100 ng/ml) stimulation, selective knockdown of CXCR4 by siRNA attenuated p38 MAPK and ERK1/2 phosphorylation. The right panel is a comparison of p38 MAPK or ERK1/2 phosphorylation after 10 minutes of stimulation with SCF (100 ng/ml). The results are the mean ± SEM from four independent experiments. (**B**) CSCs were pretreated with CXCR4 or mock siRNAs for 48 h and were then added to the upper chamber for the migration assay (lower chamber: with or without 100 ng/ml of SCF; three independent experiments). Error bars represent SEM from three independent experiments. *p < 0.05; **p < 0.01; ***p < 0.001. Full immunoblots for the cropped images presented here are provided in the Supplementary Information.

To test our hypothesis that the SCF/c-kit signalling axis may be dependent on CXCR4 function and activity, we used small interfering RNA (siRNA) to specifically knockdown CXCR4 expression. Then, CSC migration and subsequent MAPK activation were determined when cells were exposed to SCF. As shown in Fig. 3A, efficient and specific knockdown of CXCR4 expression in CSCs was achieved upon treatment with siCXCR4. Furthermore, selective knockdown of CXCR4 by siRNA attenuated SCF-induced p38 MAPK and ERK1/2 phosphorylation (Fig. 3A), which are the crucial downstream signals for cell migration. The Transwell assay results revealed that siCXCR4 significantly attenuated the effect of SCF on CSC migration (Fig. 3B).

### SCF/c-kit signaling transactivates CXCR4-serine 339 phosphorylation

The results from the CXCR4 siRNA experiment raised the possibility that CXCR4 may play an important role in SCF-induced CSC migration and the subsequent downstream MAPK activation. Recently, several studies have demonstrated that CXCR4-serine 339 phosphorylation is crucial for tumour cell migration and metastasis^12^,^17^,^18^. Although CXCR4-serine 339 phosphorylation should mainly be observed upon activation of the ligand SDF-1, it cannot include all situations. For example, EGF can stimulate CXCR4-339 phosphorylation *in vitro* in a glioblastoma multiforme cell line^23^. Thus, it is possible that SCF induces migration in CSCs and that subsequent signalling could be associated with transactivation of CXCR4-serine 339 phosphorylation.

**Figure 4:**
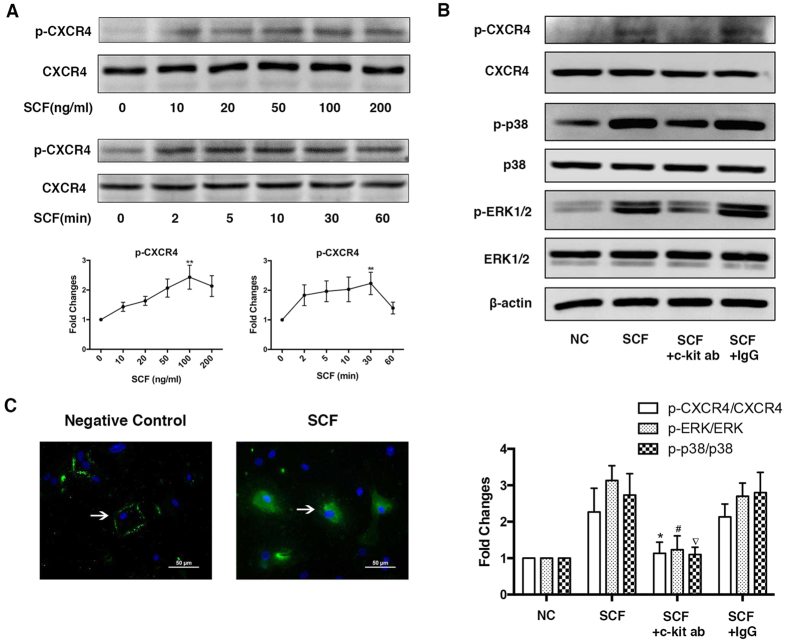
SCF/c-kit signaling transactivates CXCR4-serine 339 phosphorylation. (**A**) Dose- and time-dependent assays to determine CXCR4-serine 339 phosphorylation when exposed to SCF. Data presented as fold increase over baseline level in the control (±SEM, three independent experiments; *p < 0.05; **p < 0.01; ***p < 0.001). (**B**) CXCR4-serine 339, p38 and p-ERK1/2 phosphorylation were significantly reduced after blocking c-kit. *p < 0.05, #p < 0.05, ▿p < 0.05 (SCF+c-kit ab vs SCF). (**C**) CXCR4 internalization following SCF (100 ng/ml) stimulation for 60 minutes visualized using fluorescence microscopic analysis of CSCs. Full immunoblots for the cropped images presented here are provided in the Supplementary Information.

CXCR4-positive CSCs were then stimulated for either various times or doses with SCF, and cell lysates were electrophoresed and blotted with anti-serine(P)-339 antibody to assess the kinetics of phosphorylation. CXCR4-serine 339 was rapidly phosphorylated after 2 minutes of SCF stimulation and returned to near basal levels within 60 min (Fig. 4A). Furthermore, there was also a dose-dependent increase in the phosphorylation of CXCR4-serine 339 when the cells were exposed to SCF (Fig. 4A). Hela cells were used as positive control for CXCR4-serine 339 phosphorylation. (Supplementary Information) Additionally, the total expression of CXCR4 did not change over the whole process as assessed by western blotting and RT-PCR.

According to our previous study by Kuang *et al*.^6^ we have shown that SCF-induced CSC migration could be interrupted when blocking the cells with an anti-c-kit antibody. To further demonstrate that CXCR4-serine 339 phophorylation depends on c-kit activation while not on the directly interaction with SCF, we used SCF to stimulate CSCs after blocking c-kit, and the results showed that CXCR4 serine 339, p38 and p-ERK1/2 phosphorylation were significantly reduced after blocking c-kit (Fig. 4B).

Previous studies have identified that serine 339 is critical for SDF-1-induced internalization and degradation^17^,^24^. However, whether SCF induced CXCR4 internalization was unknown. We then examined CXCR4 surface expression of CSCs upon SCF stimulation by immunocytochemistry. In the absence of SCF, CXCR4 had a uniform membrane distribution (Fig. 4C, arrows). In contrast, SCF stimulation caused internalization of CXCR4 as visualized by marked redistribution into cellular aggregates (Fig. 4C).

### Impact of CXCR4 lacking serine 339 (LS339-CXCR4) on SCF-induced migration

**Figure 5:**
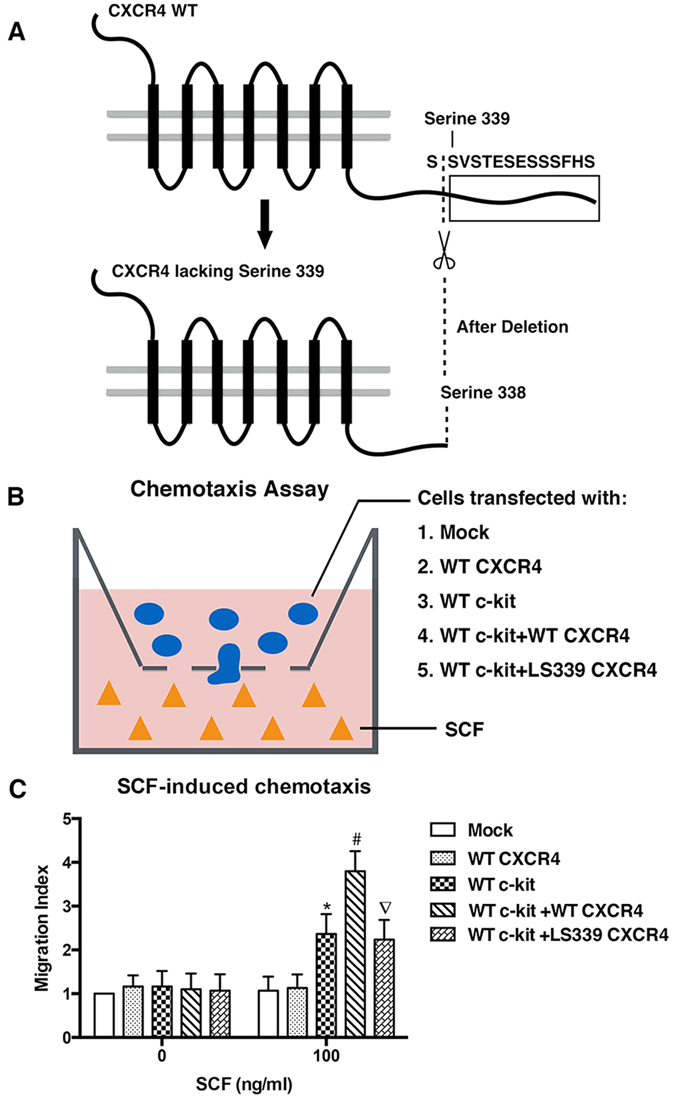
Impact of CXCR4 lacking serine 339 (LS339-CXCR4) on SCF-induced migration. (**A**) Construction of the amino-acid sequence of WT CXCR4 and the method of generating CXCR4 lacking serine 339. (**B**) Schematic representing the procedures of transfection and chemotaxis assays. (**C**) SCF-induced chemotaxis of CXCR4 variants in HEK-293 cells measured using Transwell assays. Data represent the mean ± SEM of three independent experiments performed in duplicate. *p < 0.05 (WT c-kit vs WT CXCR4); #p < 0.05 (WT c-kit+WT CXCR4 vs WT c-kit); ▿p < 0.05 (WT c-kit+LS339 CXCR4 vs WT c-kit+WT CXCR4).

To better understand the role of CXCR4-serine 339 phosphorylation in SCF/c-kit signalling, we engineered a CXCR4 variant lacking serine 339 (LS339-CXCR4) by cutting the tail off of the intracellular C-terminal domain of CXCR4 from serine 339 to the end (Fig. 5A). Then, HEK-293 cells were co-transfected with a CXCR4 plasmid (WT or LS339) and WT c-kit plasmid (Fig. 5B). HEK-293 cells were selected because of their very low expression of endogenous CXCR4 and c-kit. In a Transwell migration assay, cells only expressing WT CXCR4 barely migrate toward SCF stimulation. However, with the expression of both WT c-kit and WT CXCR4, cells showed an increased chemotaxis capacity toward SCF when compared to cells transfected with c-kit only (Fig. 5C). As expected, cells expressing LS339-CXCR4 showed decreased migration in comparison with that of WT CXCR4 (Fig. 5C). These results indicate that CXCR4 and its serine 339 phosphorylation can regulate the migration caused by SCF.

### Transactivation of CXCR4 by SCF/c-kit signaling is mediated through GRK6

Because GPCR phosphorylation and desensitization is primarily mediated by members of the GPCR kinase (GRK) family^15^,^16^,^25^ and GRK6 has been implicated in mediating CXCR4-serine 339 phosphorylation^25^, we thus used siRNA to specifically knockdown GRK6 because there are no GRK6-specific inhibitors available. Furthermore, we also used siRNA to knockdown GRK2, which is the most abundant G protein-coupled receptor kinase in the heart^26^, to compare the specific roles that these two kinases play during SCF-induced CSC migration.

**Figure 6:**
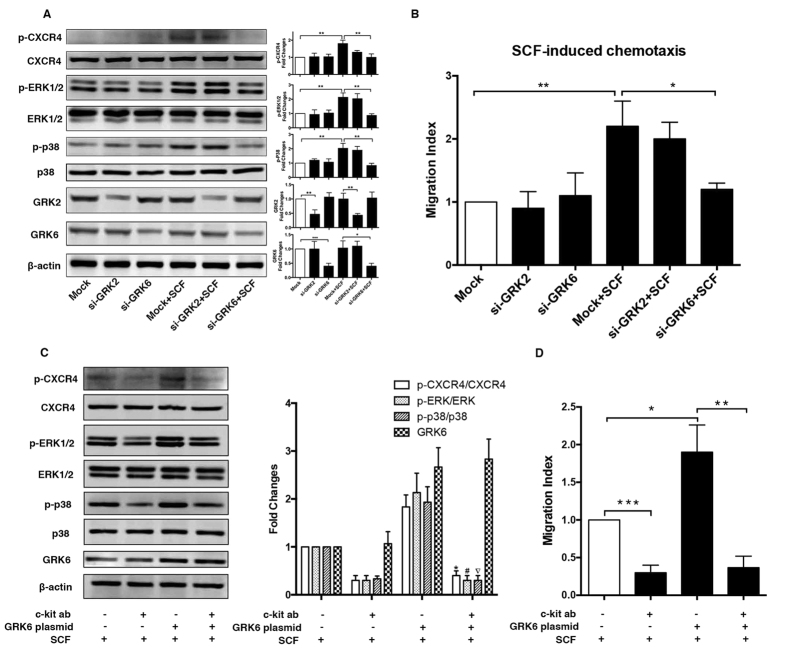
Transactivation of CXCR4 by SCF/c-kit signaling is mediated through GRK6. (**A**) CSCs were transfected with either GRK2 or GRK6 siRNAs. Then, cells were stimulated with 100 ng/ml of SCF for 10 minutes. The efficiency of transfection and the phosphorylation of CXCR4-serine 339, p38 MAPK and ERK1/2 were analysed using western blotting (left panel), and the data were quantified in the right panel (±SEM, three independent experiments). (**B**) After transfection with GRK2 or GRK6 siRNA, the chemotaxis of SCF (100 ng/ml) on CSCs was measured by the Transwell migration assay. Data represent the mean ± SEM of three independent experiments performed in duplicate. *p < 0.05; **p < 0.01; ***p < 0.001. (**C,D**) Overexpression of GRK6 with the pretreatment of c-kit blocking antibody in CSCs. CXCR4-serine 339, p38 and ERK1/2 phosphorylation and also migration in cells overexpressing GRK6 were enhanced relative to that in control cells, but after blocking c-kit, they were reduced even upon overexpression of GRK6. (**C**) Data represent the mean±SEM of three independent experiments performed in duplicate. *,#,▿p < 0.05 (c-kit ab+GRK6 plamid+SCF vs GRK6 plasmid+SCF). (**D**) Data represent the mean ± SEM of three independent experiments performed in duplicate. *p < 0.05; **p < 0.01; ***p < 0.001. Full immunoblots for the cropped images presented here are provided in the Supplementary Information.

Interestingly, cells treated with GRK6 siRNA showed dramatically decreased chemotaxis and CXCR4-serine 339 phosphorylation compared with those of the control group, whereas cells transfected with GRK2 siRNA showed no significant changes in either chemotaxis or CXCR4-serine 339 phosphorylation upon SCF treatment (Fig. 6A,B). Furthermore, when cells were transfected with only GRK6 or GRK2 siRNA, changes in ERK1/2 and p38 MAPK phosphorylation were hard to distinguish (Fig. 6A). In contrast, when cells were first treated with GRK6 siRNA and then stimulated with SCF, we found a marked decrease in p38 MAPK and ERK1/2 phosphorylation but no adverse effects toward SCF or GRK2 siRNA (Fig. 6A). These data indicated that SCF-induced CXCR4-serine 339 phosphorylation and the subsequent MAPK signalling were likely regulated by GRK6 but not GRK2.

To make more clarification for the c-kit-GRK6-CXCR4 mechanism, we transfected CSCs with WT GRK6 plasmid, pretreated the cells with a mock or c-kit blocking antibody, and then stimulated the cells with SCF. The results from western blot and Transwell assays (Fig. 6C,D) showed that CXCR4-serine 339, p38 and ERK1/2 phosphorylation and also migration in cells overexpressing GRK6 were enhanced relative to that in control cells, but after blocking c-kit, they were reduced even upon overexpression of GRK6. This demonstrates that, although GRK6 is critical for SCF-induced CSC migration and CXCR4-serine 339 phosphorylation, without c-kit activity CSCs still could not migrate toward SCF stimulation.

### GRK6 regulates CSC migration *in vivo* after myocardial infarction

Migration of CSCs to a peri-infarcted area is essential for the cells to exert their repair function. Based on the experiments *in vitro* we have showed that GRK6 is critical for CXCR4-serine 339 phosphorylation and could also regulate CSCs migration. Thus, we performed the experiment *in vivo* to investigate what extent role of GRK6 could be played in the process of CSCs migration.

**Figure 7:**
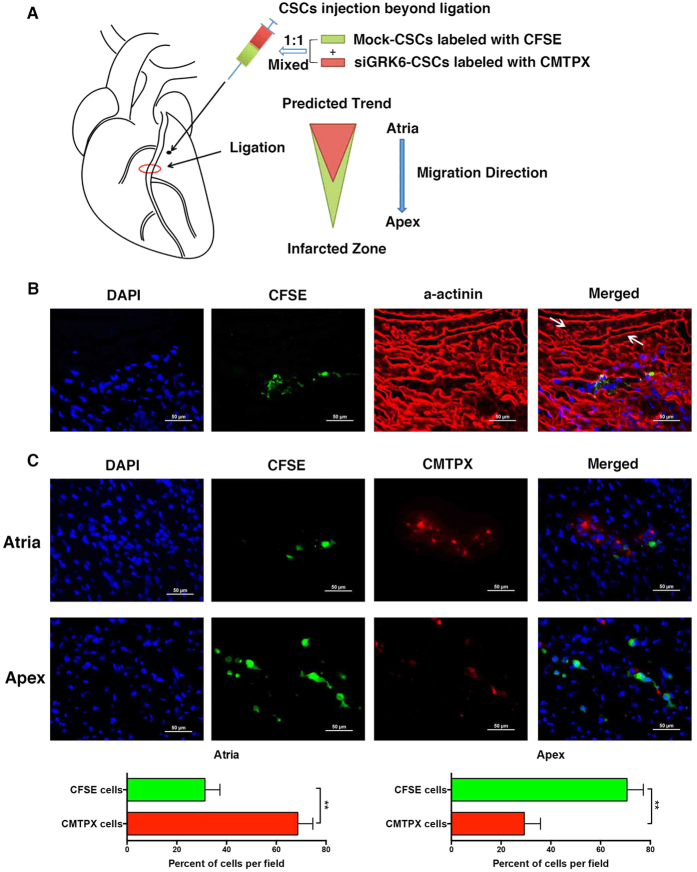
GRK6 regulates CSC migration *in vivo* after myocardial infarction. (**A**) Process diagram illustrating the series of steps for *in vivo* cell tracking. Left panel: newly isolated CSCs divided into two groups with one group transfected with GRK6 siRNA and stained with CMTPX (red) and the other transfected with mock siRNA and stained with CFSE (green) as the control. Equal numbers of cells from each group were mixed together and injected into the border zone beyond the ligature of the infarcted heart. Mice hearts were harvested 3 days later, and serial 5-μm frozen sections from the ligature to the apex were made. Right panel: predicted trend showing that the migration ability of cells transfected with GRK6 siRNA was impaired relative to that of mock cells. (**B**) With only mock cell (green) administration to the hearts, translocation and homing of CSCs to the site of myocardial injury (arrows) could be observed (n = 3–5 mice in three independent experiments). (**C**) In the heart sections near the ligature, more red cells (siGRK6 CSCs) were detected, whereas in the heart sections near the infarction site, an accumulation of green cells (mock CSCs) was detected (n = 4 mice in independent experiments). The percent of green vs red cells per field was determined by counting at least 100 cells per group in randomly selected fields upon microscope examination. **p < 0.01.

For *in vivo* CSC tracing, mock and siGRK6 CSCs were labelled with CFSE and CMTPX, respectively, and an equal number of cells were mixed together before being injected into the border zone of an infarcted heart (Fig. 7A). Three days later, the animals were euthanized; their hearts were harvested, and fluorescence was assessed by fluorescent microscopy.

As expected, when the heart was only administered with mock cells, we observed the translocation and homing of CSCs to the site of myocardial injury (Fig. 7B). When the hearts were administered both types of CSCs (Fig. 7C, mock cells in green and siGRK6 cells in red), we found a significant reduction in the accumulation of siGRK6 CSCs in the peri-infarcted regions, especially the region near the apex, where almost only mock cells could be found. These results confirm that knockdown of GRK6 affects the migration ability of CSCs.

## Discussion

**Figure 8:**
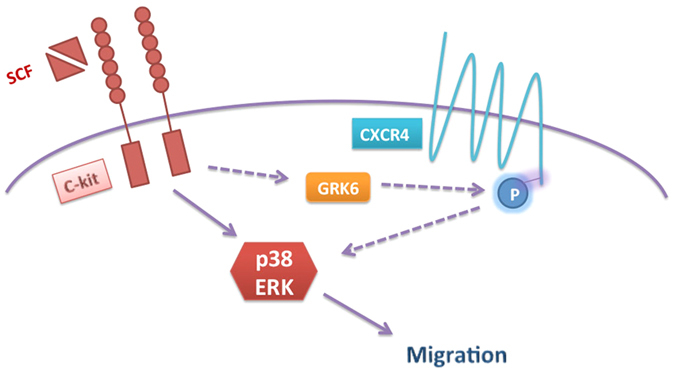
Schematic representation of crosstalk between c-kit and CXCR4. SCF/c-kit induced CXCR4-serine 339 phosphorylation is regulated by GRK6, and the subsequent downstream signalling and migration caused by SCF in CSCs require CXCR4-serine 339 phosphorylation and GRK6 activity.

According to our previous study, SCF (c-kit ligand) has a promising effect on CSC migration via activation of p38 MAPK^6^. However, inhibition of p38 MAPK by SB203580 did not completely inhibit CSC migration, suggesting that other signalling pathways may be involved in SCF-induced CSC migration. Cheng *et al*. have demonstrated potential cross-talk between CXCR4 and c-kit such that CXCR4-mediated mobilization is modulated by c-kit activity in bone marrow progenitor cells^22^. Here, we present evidence that SCF-induced migration and subsequent signalling in CSCs are regulated by CXCR4 activity (Fig. 8). Furthermore, the basis of this cross-talk appears to depend on CXCR4 transactivation by SCF/c-kit signalling.

For testing this hypothesis, CXCR4 siRNA was used to knockdown CXCR4 expression. Our results showed that SCF-induced CSC migration was significantly attenuated by pretreating cells with CXCR4 siRNA. Furthermore, the SCF-induced downstream signalling activation, e.g., p38 MAPK or ERK1/2, were also impaired after CXCR4 siRNA treatment.

CXCR4 is a seven transmembrane-spanning G protein-coupled receptor (GPCR) with no intrinsic kinase activity. Multiple serines and threonines, as well as tyrosines, can be phosphorylated in response to both ligand binding and activity in parallel signalling pathways^27^. To evaluate the role of CXCR4 in SCF-induced CSC migration, we sought to correlate CXCR4 phosphorylation and function.

Classically, the phosphorylation of CXCR4 on serine 339 was mainly observed by SDF-1 stimulation, which indicates that ligand activation of CXCR4 is the predominant mechanism for regulating its activity. However, it has been demonstrated that EGF receptor activation can transactivate CXCR4-serine 339 phosphorylation in a glioblastoma multiforme cell line^23^. In our study, SCF could promote CXCR4-serine 339 phosphorylation in CSCs, and this effect could be abolished by a c-kit blocking antibody. These results indicated on one hand that CXCR4-serine 339 phosphorylation needs activated c-kit signalling first and on the other hand confirms the concept that different forms of cross-talk between GPCR and RTK systems can be found in different cellular systems. Additionally, after SCF stimulation, we observed the internalization of CXCR4. It is reported that CXCR4-serine 339 phosphorylation is related to its internalization^23^,^31^. Normally, SDF-1 induced CXCR4 internalization is regarded as a mark of receptor desensitization and also plays a role in the downstream signaling and cells functions such as MAPK activation and cells migration in the particular cell types^14^,^32^. And in our study, we have observed CXCR4-serine 339 phosphorylation and internalization in response to SCF stimulation. Thus, CXCR4 internalization may participate in the SCF induced signaling and cell functions, however, the precise mechnism remains to be elucidated.

Previous studies have shown that site-specific phosphorylation of different serine residues (Ser-321, Ser-324, Ser-325, Ser-330, Ser-339, Ser-346 and Ser-352) in the intracellular C-terminal domain of CXCR4 is involved in regulating its function^25^,^27^. Interestingly, there is increasing evidence showing that CXCR4-serine 339 phosphorylation is important in solid cancer cells for regulating cellular adhesion, retention and mobilization^17^,^18^,^24^, and it has also been reported that CXCR4-serine 339 is significantly associated with poor overall survival in B-cell acute lymphoblastic leukaemia^17^. Thus, CXCR4-serine 339 phosphorylation may not only be a concomitant phenomenon in the presence of SCF stimulation but may also play an important role in SCF-induced cell migration.

On the basis of this hypothesis, we co-transfected HEK-293 cells with WT c-kit plasmid and WT CXCR4 or LS339 plasmids to understand the role that CXCR4-serine 339 plays after SCF stimulation^17^. Upon SCF stimulation, cells could barely migrate when only expressing WT CXCR4; however, when cells were co-transfected with WT c-kit and WT CXCR4 plasmids, they showed a substantial increase in their chemotaxis when compared to cells only expressing c-kit. Surprisingly, cells expressing LS339-CXCR4, on the contrary, had decreased chemotaxis compared to those with WT CXCR4. These results suggest that SCF-induced cell chemotaxis could be dependent on CXCR4 serine 339.

It has been well-defined that GPCR phosphorylation and desensitization is primarily mediated by members of the GPCR kinase (GRK) family^15^,^16^,^25^. GRKs are divided into three subfamilies: GRK1 (GRK 1 and 7), GRK2 (GRK 2 and 3), and GRK4 (GRK 4, 5 and 6)^28^,^29^. Different GRKs can phosphorylate different carboxyl terminus sites of GPCRs. GRK6 phosphorylates multiple sites, including Ser-324/5, Ser-330, and Ser-339, whereas GRK2, -3, and -5 do not contribute to CXCR4 phosphorylation at these sites^25^; thus, we used GRK6 and GRK2 siRNAs to specifically knockdown their expression. SiGRK6 CSCs showed a reduced migration ability combined with attenuation of CXCR4-serine 339 phosphorylation and MAPK activation upon SCF stimulation, whereas GRK2 knockdown in CSCs did not have the same effect. These results demonstrated that GRK6 plays an important role in SCF/c-kit induced CXCR4-serine 339 phosphorylation and CSCs migration. Furthermore, the *in vivo* experiments also clearly showed that GRK6 was involved in the CSCs migration during myocardial infarction. Interestingly, overexpression of GRK6 could not restore the migration ability of CSCs when c-kit activity was blocked by an antibody. It indicated that despite the critical role of GRK6, c-kit activity is necessary for SCF-induced CSC migration and CXCR4-serine 339 phosphorylation.

Although we know that there are multiple growth factors and chemoattractive factors upregulated during the MI process, our lab has focused on SCF and has more interest in its effect on chemotaxis. This is because, on one hand, SCF is the only known ligand for c-kit to date, and SCF/c-kit signalling participates in a wide range of cellular behaviours^30^. On the other hand, despite the effect of SCF on chemotaxis, SCF has shown more attractive functions in CSCs, such as activating quiescent CSCs *in situ* to re-enter the cell cycle and contributing to the reversal of myopathy associated with aging^7^. Thus, we believe that SCF treatment may be a potential strategy for the treatment of myocardial diseases.

## Conclusions

Our study revealed a new pathway for cross-talk between c-kit and CXCR4. SCF/c-kit signalling-induced cardiac stem cell migration is regulated by the transactivation of CXCR4-serine 339 phosphorylation, and GRK6 may play a crucial role in SCF-induced CXCR4-serine 339 phosphorylation and participate in SCF-induced cardiac stem cell migration and downstream signalling.

## Methods

### Ethics statement

All animal studies were performed in accordance with the Guidelines of the Hubei Council of Animal Care and approved by the Experimental Animals Committee of the Huazhong University of Science and Technology in China.

### Isolation and culture of CSCs

CSCs were isolated from the hearts of wild type male C57BL/6 (male or female) mice (8 weeks) using a method described previously with a minor modification. Briefly, the hearts were cut into pieces, and washed and digested 2 times with Liberase TH (Roche) at 37 °C for 5 minutes. The digested tissue fragments were then cultured as explants in Ham’s F12 medium supplemented with 10% foetal bovine serum (Gibco), L-glutamine (2 mmol/L; Gibco), 2-mercaptoethanol (0.1 mmol/L; Gibco) and antibiotics. After two to three weeks of growth, the cells were digested and subjected to immunomagnetic sorting with microbeads (Miltenyi Biotech) to obtain c-kit positive CSCs. The isolated CSCs were cultured in DMEM/F12 medium containing 10% foetal bovine serum, LIF (10 ng/ml; Millipore), bFGF (10 ng/ml; Peprotech), EGF (20 ng/ml; Peprotech), insulin-transferrin-selenite (ITS; Invitrogen) and antibiotics. Anti-c-kit blocking antibody (eBioscience) was used to block c-kit activity by pretreated cells with 20 ug/ml for 2 hours in some experiments.

### Immunocytochemistry

CSCs were fixed and labelled with c-kit and CXCR4 antibodies. After overnight incubation, cells were washed 3 times with PBS and then stained with Alexa-Fluor 488- or Alexa-Fluor 594-conjugated secondary antibodies. Nuclei were visualized by DAPI labelling. Images were acquired using Nikon fluorescence microscopy.

### Flow cytometry

For examination of the expression of stem cell surface markers, CSCs were trypsinized, resuspended in phosphate buffered saline (PBS) and blocked with 5% FBS for 15 min. CSCs were then labelled with a monoclonal c-kit(PE) or CXCR4(PE) antibody in a dark room for 30 min and then washed twice with cold PBS. Data were collected and analysed using a FACSCalibur flow cytometer (BD Biosciences, USA).

### siRNA transfection

Small interfering RNA (siRNA stock solution: 50 μmol/L) transfection is performed with cells plated in 24-well dishes and transfected with 0.5 μL of siRNA and 3 μL of Lipofectamine RNAiMAX reagent (Life Technologies) in 50 μL of Opti-MEM medium. All siRNAs were commercially synthesized at Ambion (Life Technologies, USA).

### Plasmid constructs and transfection of HEK-293 cells

The plasmids for wild type GRK6 (WT GRK6), wild type c-Kit (WT c-Kit) and wild type CXCR4 (WT CXCR4) were purchased from Vigene Biosciences (CXCR4: CH864538; c-Kit: CH805968), and the CXCR4 lacking serine 339 (LS339-CXCR4) plasmid was generated using PCR. HEK-293 cells were grown in 6-well plates (Corning, Tissue Culture Treated) with Dulbecco’s Modified Eagle medium containing 10% foetal calf serum and antibiotics in the presence of 5% CO2 in a humidified atmosphere. When cells reached 60% confluence, they were co-transfected with c-Kit WT plasmid and either CXCR4 WT or LS339-CXCR4 plasmid using Lipofectamine 3000 Reagent (Invitrogen). After 2 days of culture, the cells were trypsinized for further experiments.

### Chemotaxis assay

Cells resuspended in DMEM/F12 (100 μl) were placed in the upper well of a Transwell chamber (8.0-μm pore size; Corning). For chemotaxis, a gradient of SCF (0–200 ng/ml) was applied in the lower chamber. For transfection experiments, cells were pretreated with different transfections before being transferring to the upper well of the Transwell chamber. After 12 h, the membrane was removed and scraped to remove non-migrating CSCs from the upper surface, and the number of cells that had migrated to the lower chamber was determined.

### Western blotting

Crude cell extracts were prepared by lysing cells using RIPA lysis buffer (Pierce) supplemented with phosphatase and protease inhibitor cocktails (Roche). The extracted proteins (20–50 μg) were resolved with 10% SDS polyacrylamide gels and transferred onto a nitrocellulose membrane (Millipore) according to standard protocols. Polyclonal anti-CXCR4 antibody (1:1000, Abcam), anti-c-kit antibody (1:500, Abcam), anti-CXCR4-phospho-serine 339 antibody (1:500, Abcam), anti-ERK1/2 antibody (1:1000, Cell Signalling Technology), anti-phospho-ERk1/2 antibody (1:1000, Cell Signalling Technology), anti-p38 antibody (1:1000, Cell Signalling Technology), anti-phospho-p38 antibody (1:1000, Cell Signalling Technology), anti-GRK6 antibody (1:1000, Santa Cruz), and anti-GRK2 antibody (1:1000, Santa Cruz) were used overnight at 4 °C. This was followed by incubation with a horseradish peroxidase-conjugated secondary antibody (1:10,000; Pierce). Peroxide activity was detected using the enhanced chemiluminescence Supersignal West Dura system (Pierce). As a loading control, mouse anti-beta-actin antibody was used at a concentration of 1:1,000.

### Quantitative real-time PCR

Total RNA was extracted from CSCs with Trizol reagent (Invitrogen) following the manufacturer’s protocol. Real-time PCR was carried out using the following primers: CXCR4: sense, 5′-GACTGGCATAGTCGGCAATG-3′; anti-sense, 5′-AGAAGGGGAGTGTGATGACAAA-3′, yielding a 131-bp product; GRK6: sense, 5′-GAGAACATCGTAGCGAACACG-3′; anti-sense, 5′-ACAGAACTCACGAAATAACAGGC-3′, yielding a 204-bp product; GRK2: sense, 5′-GGGGACGTGTTCCAGAAGTTC-3′; anti-sense, 5′-ATTCGATGCACACTGAAGTCAT-3′, yielding a 113-bp product; and beta-actin: sense, 5′-GATATCGCTGCGCTGGTC-3′; anti-sense, 5′-CATCACACCCTGGTGCCTA-3′, yielding a 123-bp product. Duplicate real-time quantitative PCR was performed to analyse CXCR4, GRK6, and GRK2 mRNA expression by monitoring the increase in fluorescence of the SYBR Green dye using iTaq (Bio-Rad, Hercules, CA, USA) in accordance with the manufacturer’s instructions. Beta-actin was used as an internal standard to verify equal PCR product loading for each experiment. The relative abundance of mRNA was determined from the CT values and was plotted as fold-change compared with control.

### Myocardial infarction and analysis of CSC migration and tracing *in vivo*

Under ketamine and acepromazine anaesthesia, myocardial infarction was generated in male C57 wild type mice at 2 months of age by ligation of the left coronary artery near its origin. Newly isolated CSCs were divided into two groups. In one group, cells were transfected with GRK6 siRNA, whereas in another group, cells were transfected with mock siRNA as a control. Then, cells from the first group were stained with CMTPX, and control cells were stained with carboxyfluorescein diacetate succinimidyl ester (CFSE) dyes (Molecular Probes) according to the manufacturer’s instructions. Equal numbers of cells from each group were mixed together and injected into the border zone beyond the ligature of the infarcted heart. Mice hearts were harvested 3 days later, and serial 5-μm frozen sections from the ligature to the apex were made. Following fixation, myocytes were labelled by a-cardiac actinin, and stained cells were identified by fluorescence microscopy (Nikon).

### Statistical analysis

All values are expressed as the mean ± SEM. For analysis of differences between two groups, Student’s t test was performed. For multiple groups, ANOVA was carried out followed by Student–Newman–Keuls test with P < 0.05 considered as statistically significant.

## Additional Information

**How to cite this article**: Zuo, K. *et al*. SCF/c-kit transactivates CXCR4-serine 339 phosphorylation through G protein-coupled receptor kinase 6 and regulates cardiac stem cell migration. *Sci. Rep*. **6**, 26812; doi: 10.1038/srep26812 (2016).

## Supplementary Material

Supplementary Information

Supplementary Figures
