# Supplementary material for: SCF/c-kit transactivates CXCR4-serine 339 phosphorylation through G protein-coupled receptor kinase 6 and regulates cardiac stem cell migration
Source: Sci Rep. 2016 Jun 1;6:26812. doi: 10.1038/srep26812 (PMC4887787; doi:10.1038/srep26812)
Supplement: Supplementary Information [file srep26812-s1.pdf]

SCF/c-kit transactivates CXCR4-serine 339 phosphorylation through G protein-coupled receptor kinase 6 and regulates cardiac stem cell migration

Ke Zuo[a,b,*], Dong Kuang[a,*], Ying Wang[a,b], Yanli Xia[a,b]

Weilin Tong[a,b], Xiaoyan Wang[a], Yaobin Chen[a], Yaqi Duan[a,b¶], Guoping Wang[a,b¶]

[a] Institute of Pathology, Tongji Hospital, Tongji Medical College, Huazhong University of Science and Technology, Wuhan 430030, P. R. of China
[b] Department of Pathology, School of Basic Medicine, Tongji Medical College, Huazhong University of Science and Technology, Wuhan 430030, P. R. of China

* These authors contributed equally to the present study

¶ Corresponding authors

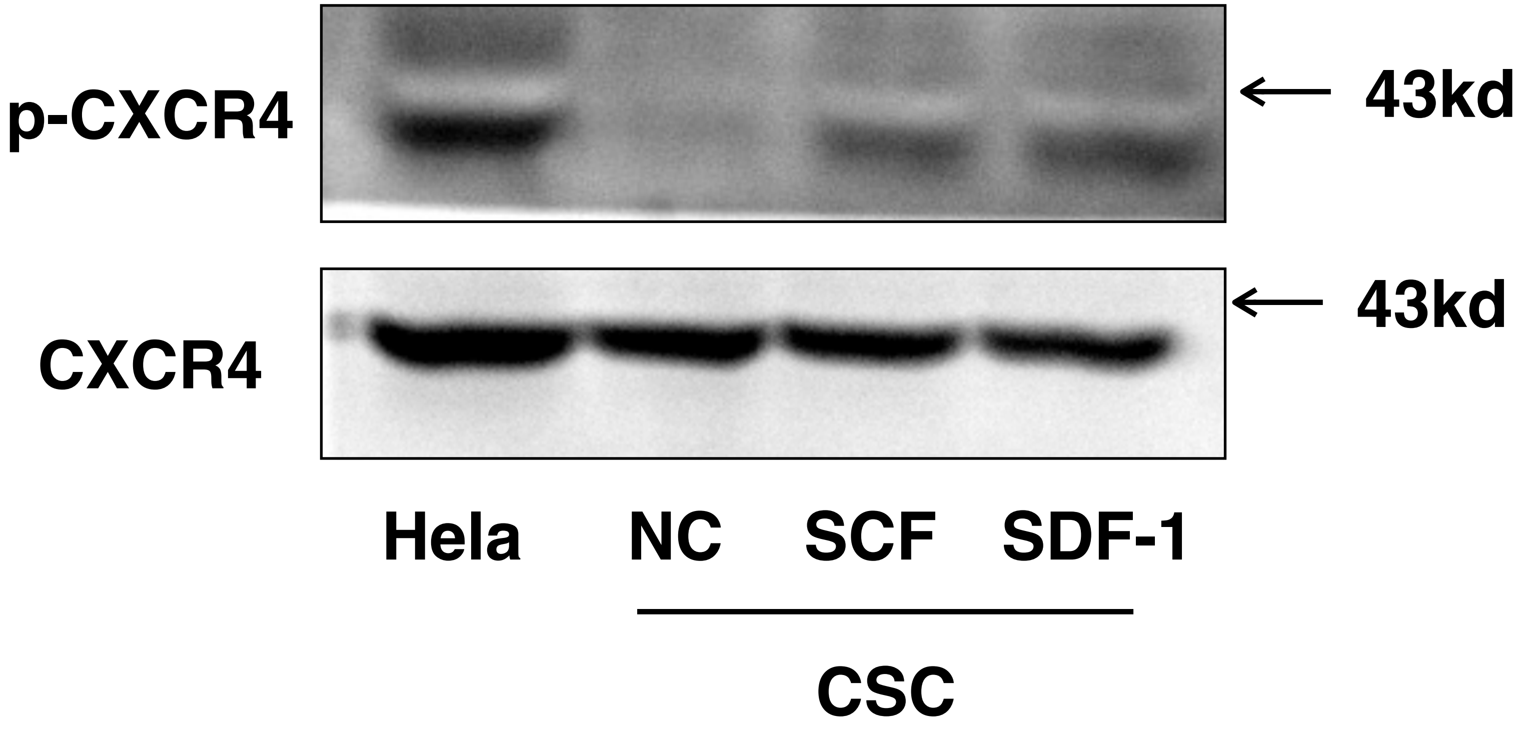
p-CXCR4
43kd
CXCR4
43kd
Hela
NC
SCF
SDF-1
CSC
