# Supplementary material for: SCF/c-kit transactivates CXCR4-serine 339 phosphorylation through G protein-coupled receptor kinase 6 and regulates cardiac stem cell migration
Source: Sci Rep. 2016 Jun 1;6:26812. doi: 10.1038/srep26812 (PMC4887787; doi:10.1038/srep26812)

SCF/c-kit transactivates CXCR4-serine 339 phosphorylation through G protein-coupled receptor kinase 6 and regulates cardiac stem cell migration

Ke Zuo[a,b,*], Dong Kuang[a,*], Ying Wang[a,b], Yanli Xia[a,b]

Weilin Tong[a,b], Xiaoyan Wang[a], Yaobin Chen[a], Yaqi Duan[a,b¶], Guoping Wang[a,b¶]

[a] Institute of Pathology, Tongji Hospital, Tongji Medical College, Huazhong University of Science and Technology, Wuhan 430030, P. R. of China

[b] Department of Pathology, School of Basic Medicine, Tongji Medical College, Huazhong University of Science and Technology, Wuhan 430030, P. R. of China

* These authors contributed equally to the present study

¶ Corresponding authors

Figure. 1E CXCR4 expression Experiment 1 (Representative)

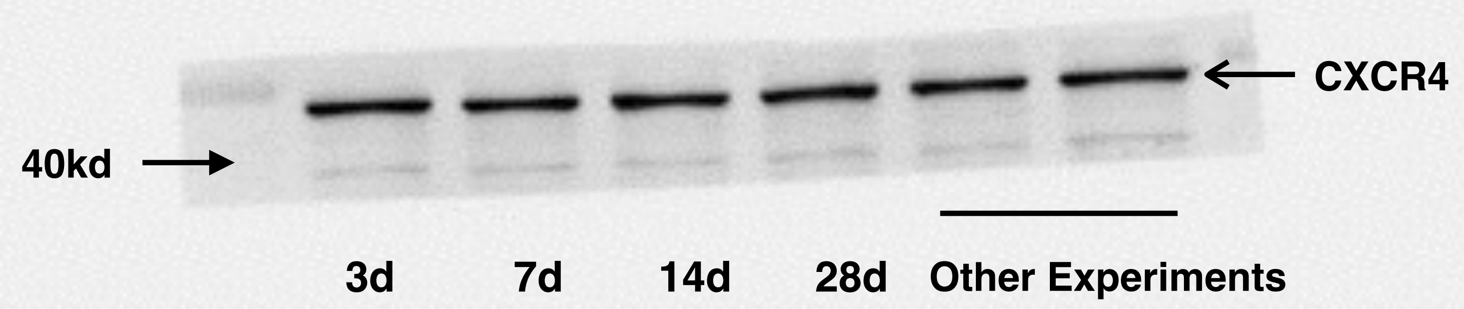

Figure. 1E CXCR4 expression Experiment 2 (Repeated)

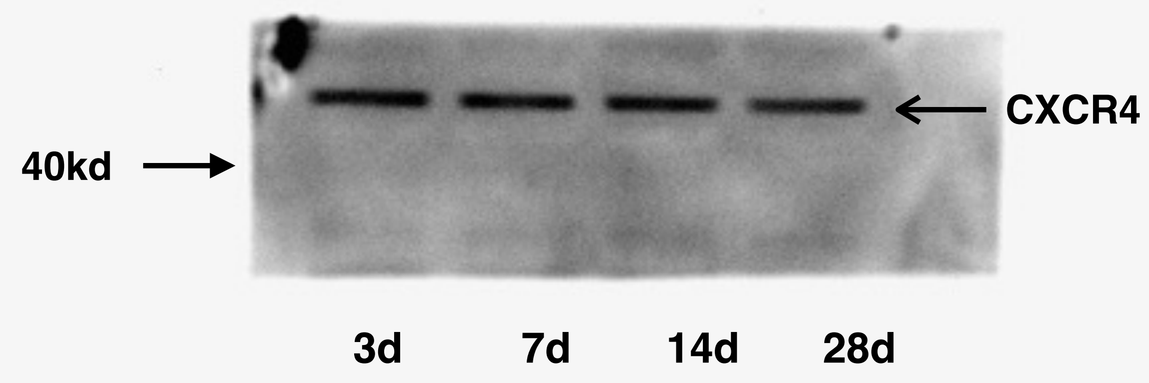

Figure. 1E CXCR4 expression **Experiment 3 (Repeated)**

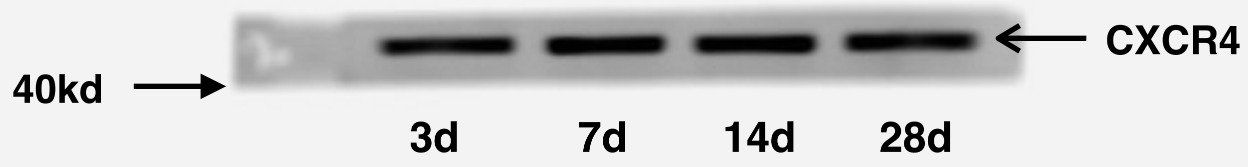

Figure. 1E c-kit expression Experiment 1 (Representative)

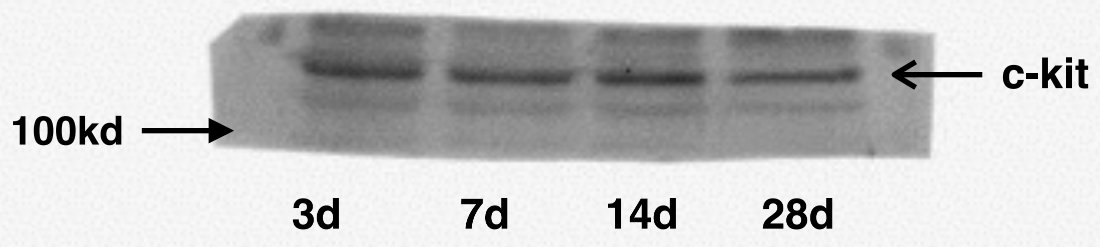

Figure. 1E c-kit expression **Experiment 2 (Repeated)**

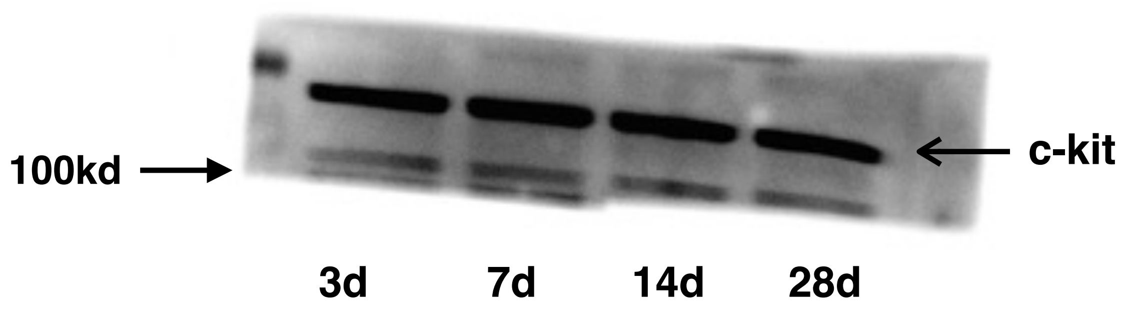

Figure. 1E c-kit expression Experiment 3 (Repeated)

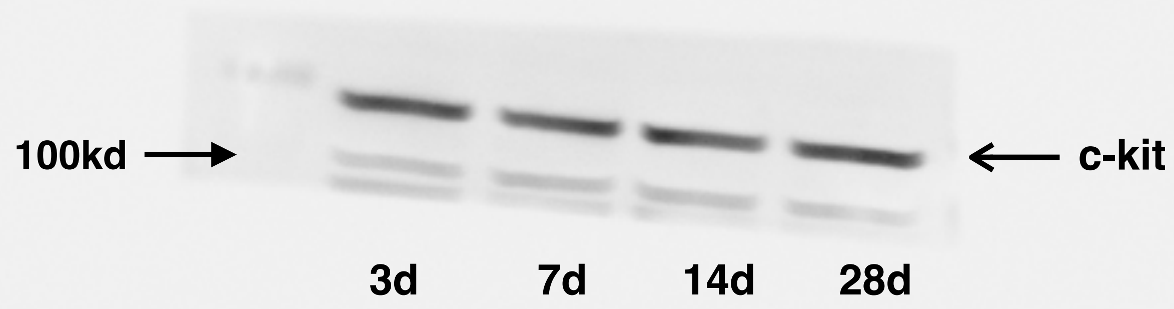

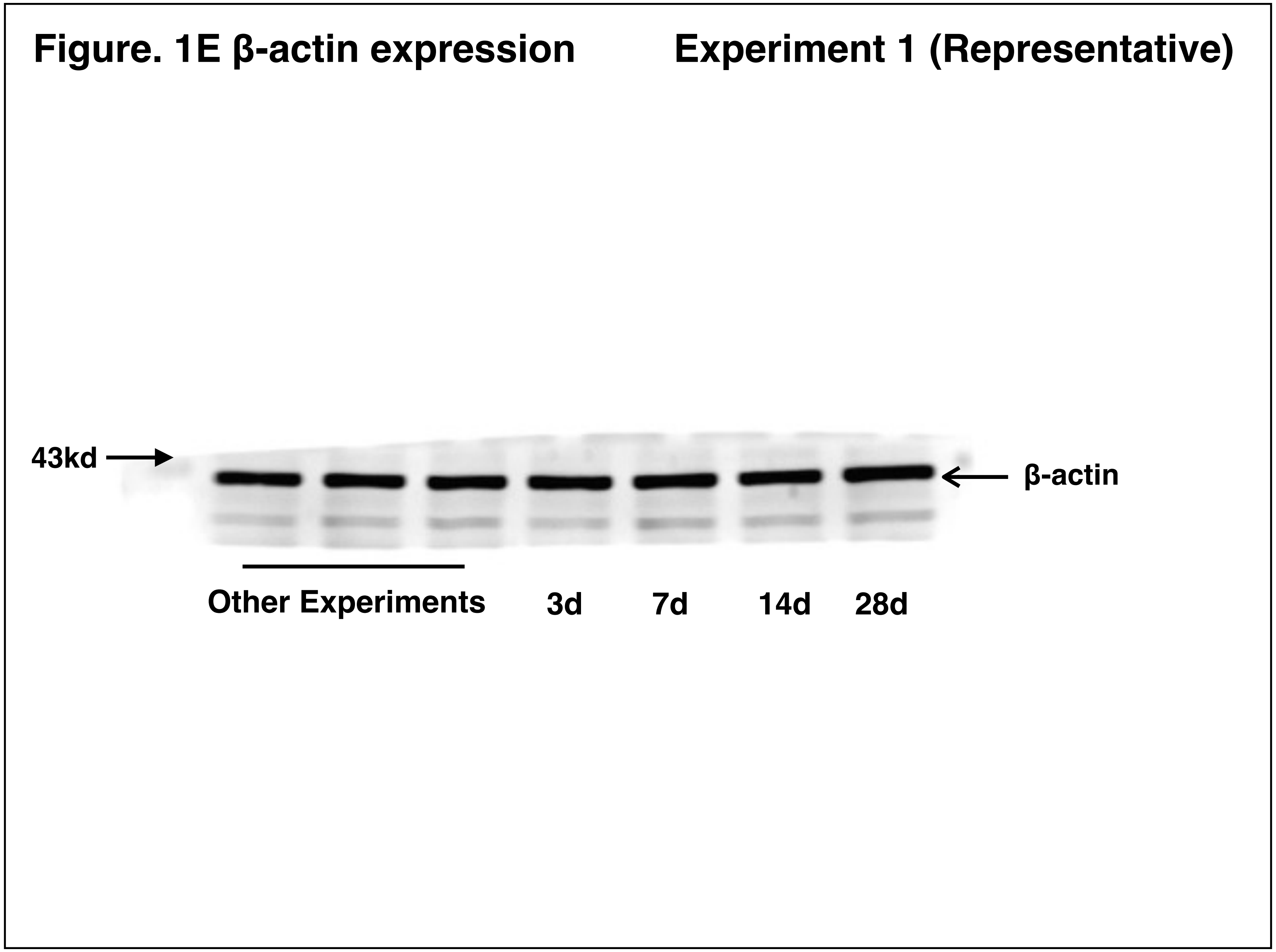
Figure. 1E β-actin expression
Experiment 1 (Representative)
43kd
β-actin
Other Experiments
3d
7d
14d
28d

Figure. 1E β-actin expression Experiment 2 (Repeated)

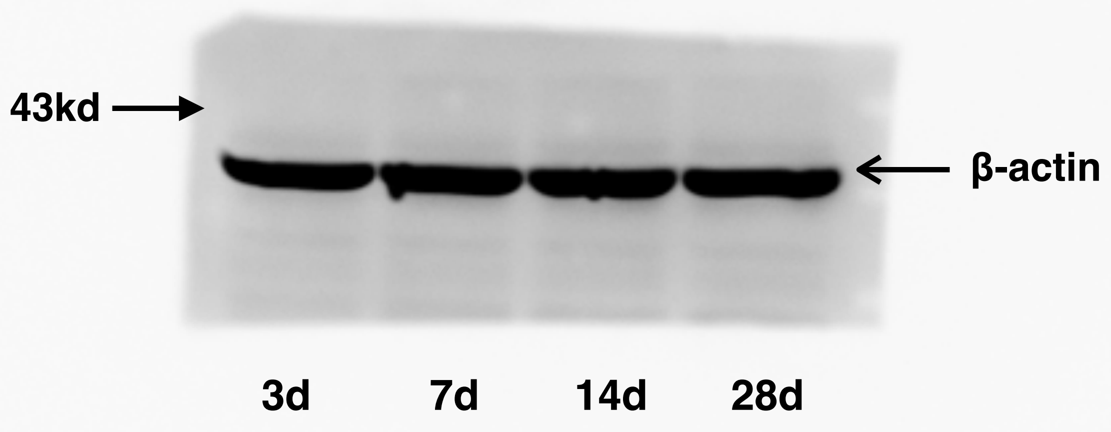

Figure. 1E β-actin expression Experiment 3 (Repeated)

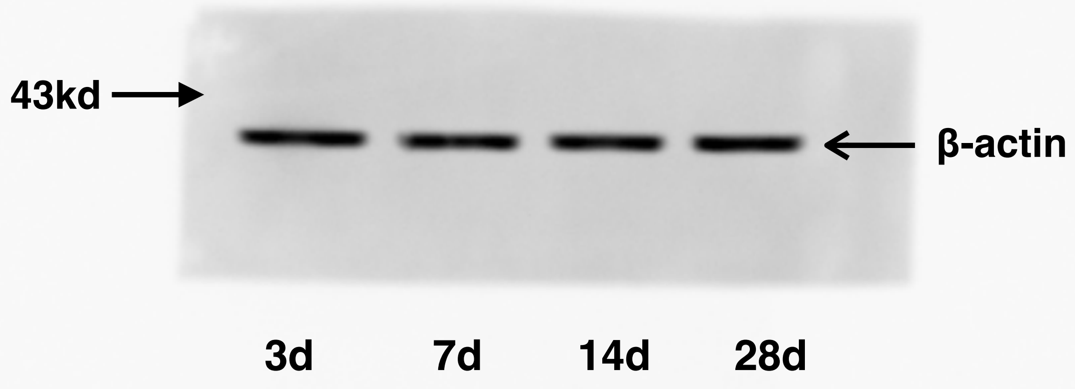

Figure. 2B p-ERK1/2 of dose dependent experiment

Experiment 1(Representative)

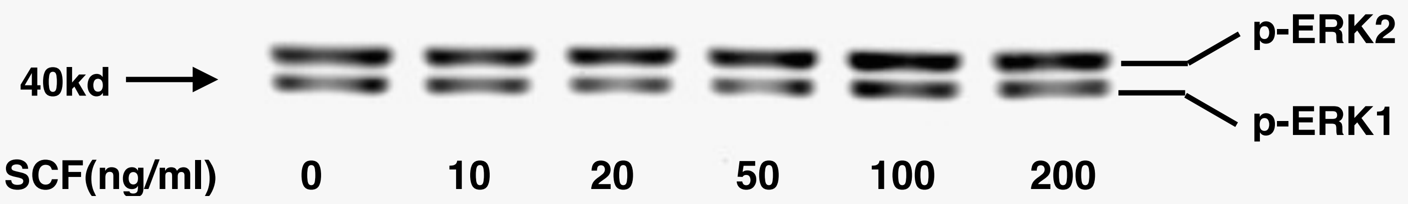

Figure. 2B p-ERK1/2 of dose dependent experiment

Experiment 2 (Repeated)

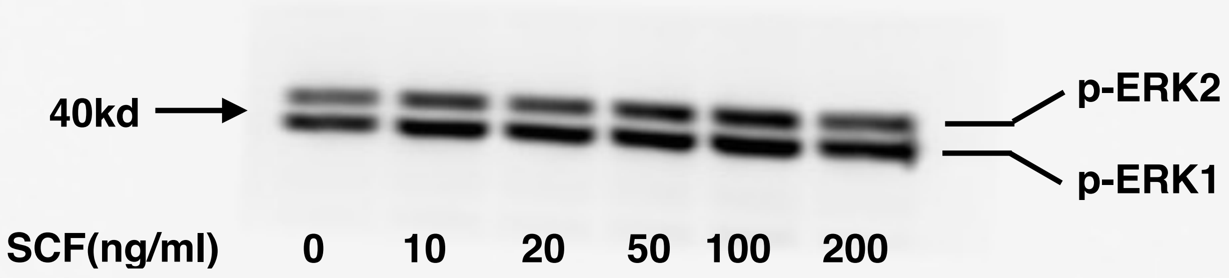

Figure. 2B p-ERK1/2 of dose dependent experiment

Experiment 3 (Repeated)

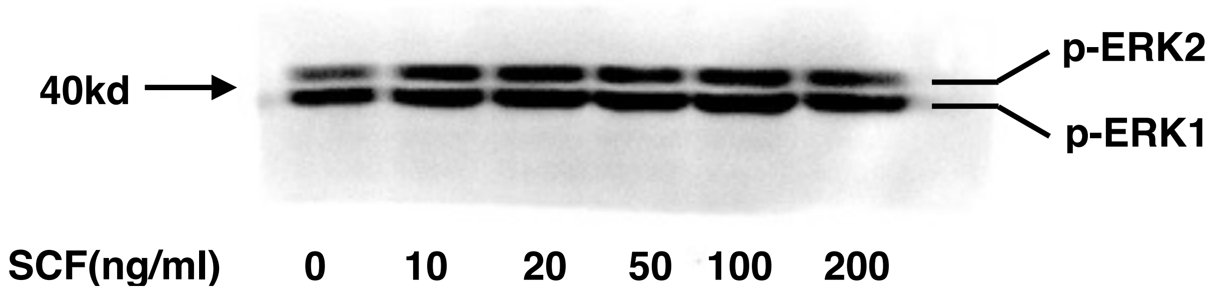

Figure. 2B ERK1/2 of dose dependent experiment

Experiment 1 (Representative)

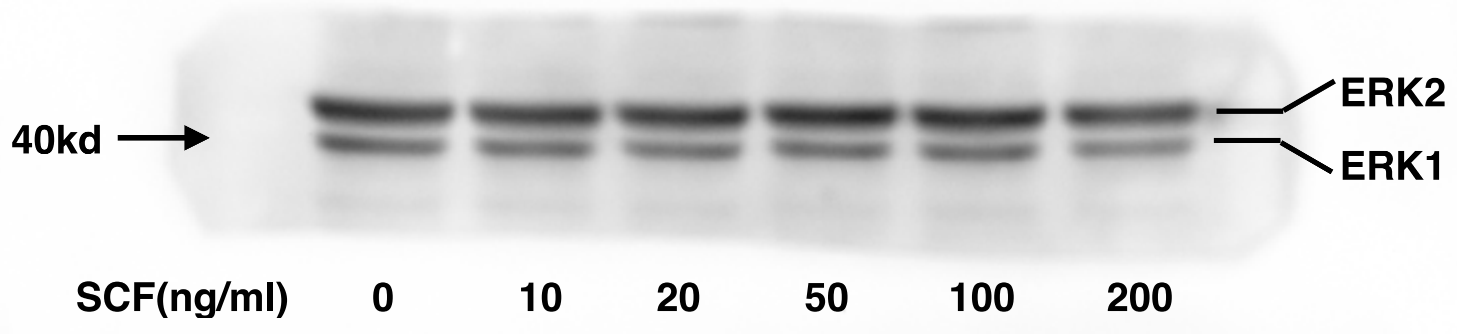

Figure. 2B ERK1/2 of dose dependent experiment

Experiment 2 (Repeated)

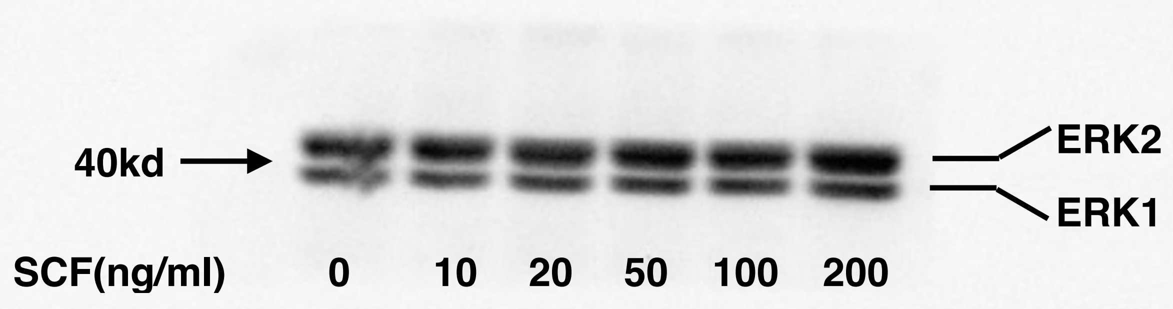

Figure. 2B ERK1/2 of dose dependent experiment

Experiment 3 (Repeated)

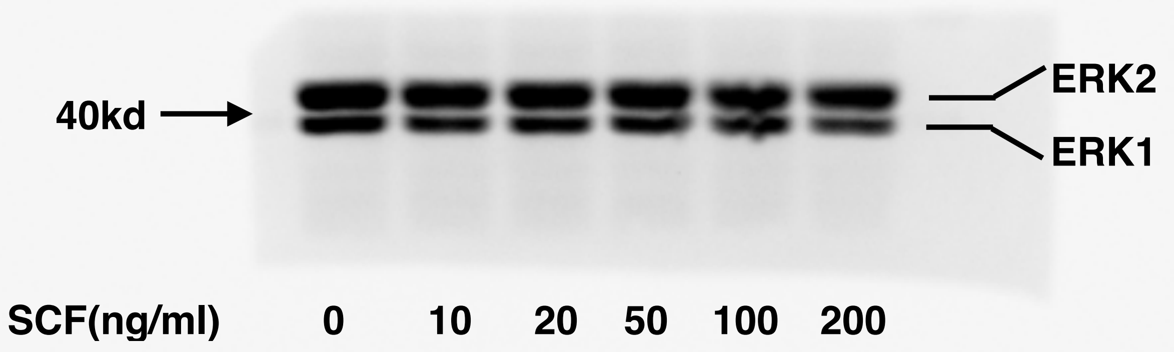

Figure. 2B p-p38 of dose dependent experiment

Experiment 1 (Representative)

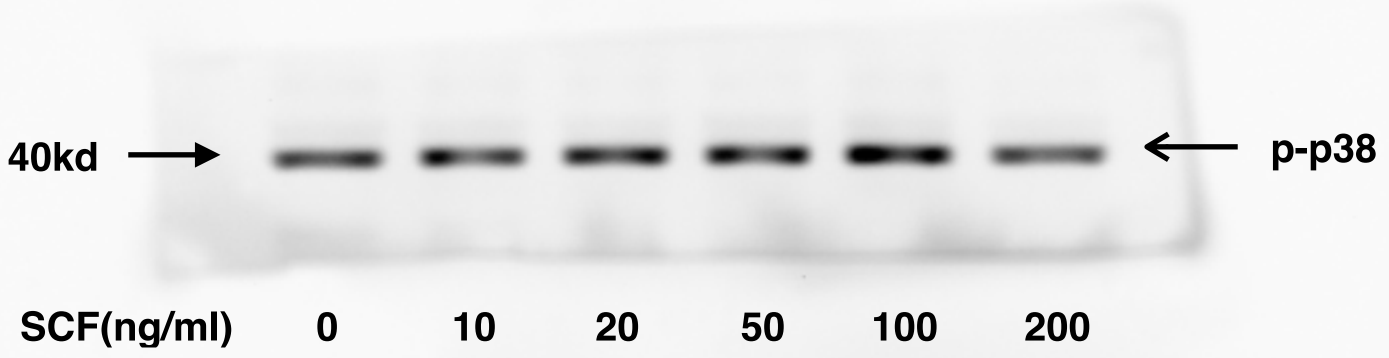

Figure. 2B p-p38 of dose dependent experiment

Experiment 2 (Repeated)

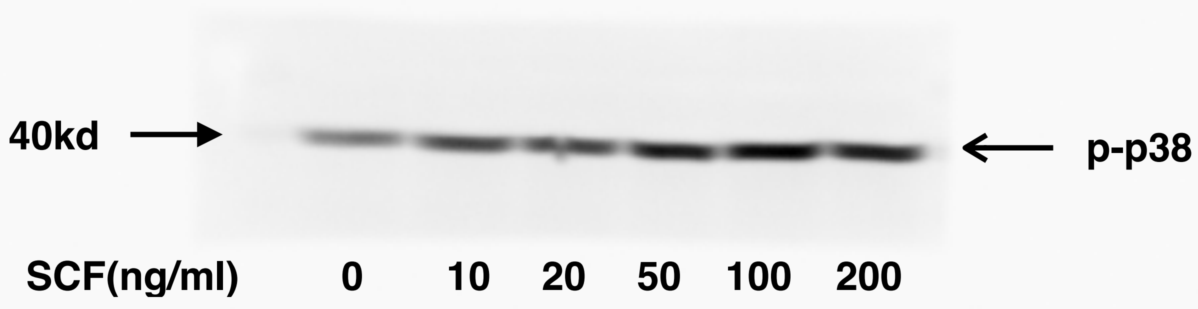

Figure. 2B p-p38 of dose dependent experiment

Experiment 3 (Repeated)

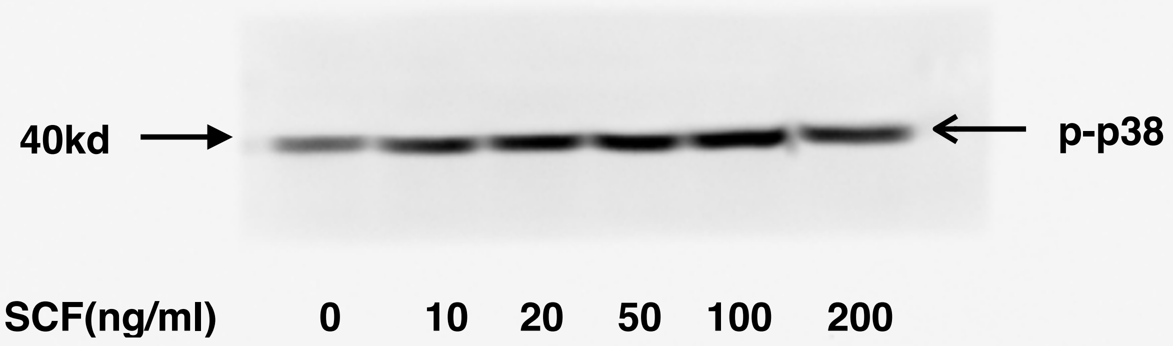

Figure. 2B p38 of dose dependent experiment

Experiment 1 (Representative)

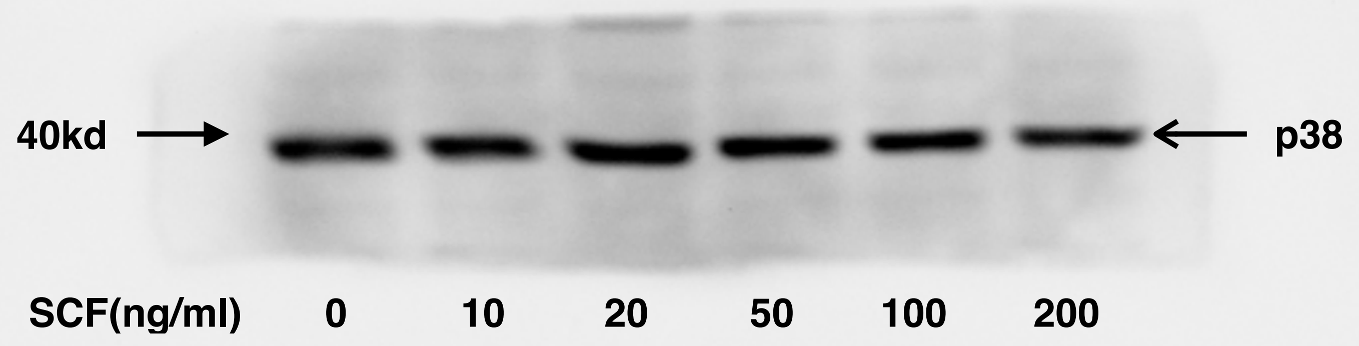

Figure. 2B p38 of dose dependent experiment

Experiment 2 (Repeated)

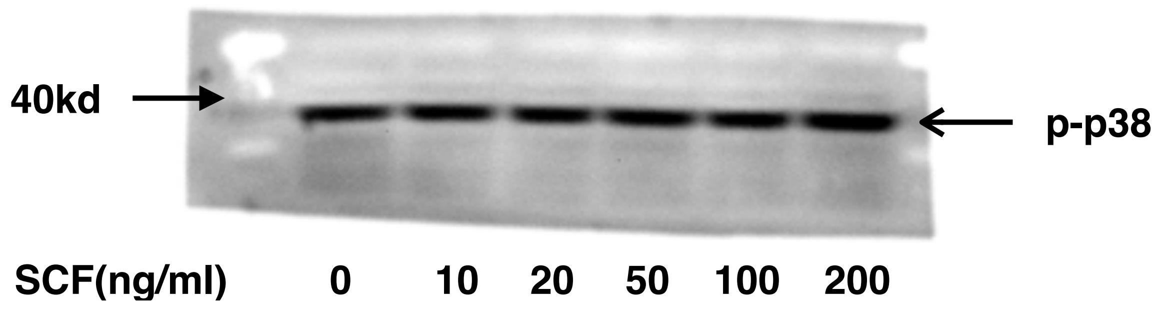

Figure. 2B p38 of dose dependent experiment

Experiment 3 (Repeated)

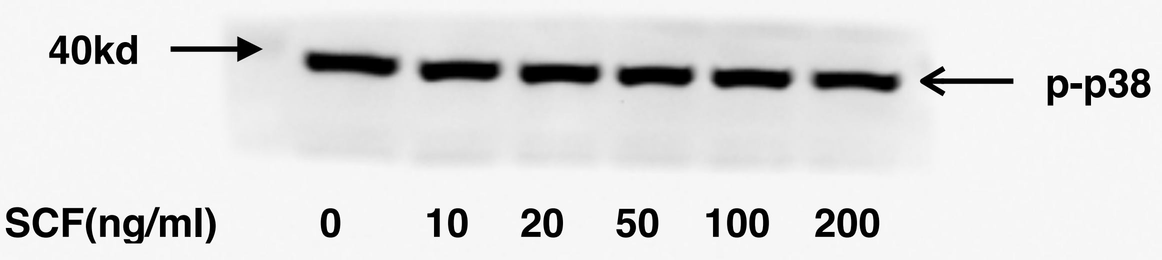

Figure. 2B β-actin of dose dependent experiment

Experiment 1 (Representative)

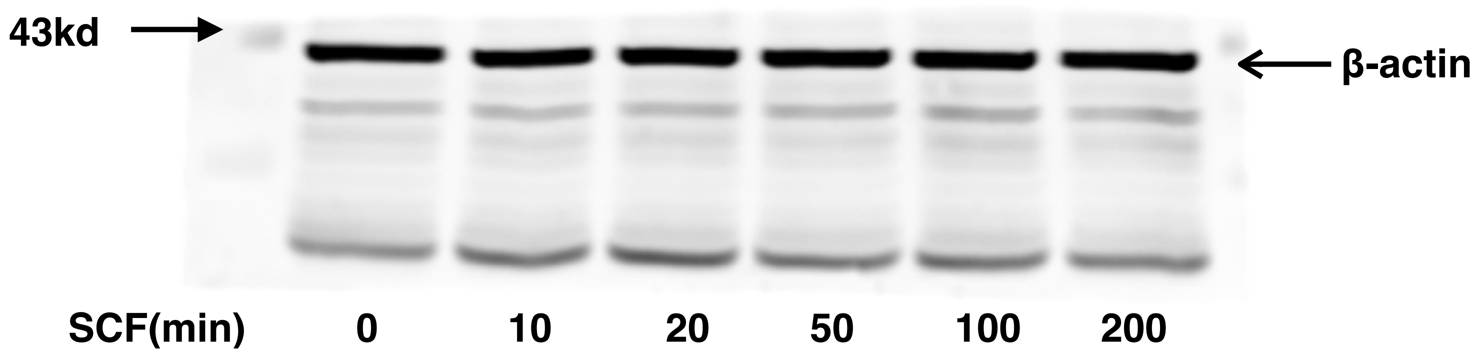

Figure. 2B β-actin of dose dependent experiment

Experiment 2 (Repeated)

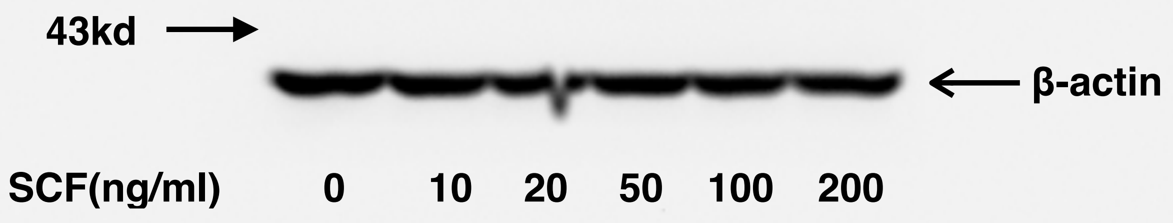

Figure. 2B β-actin of dose dependent experiment

Experiment 3 (Repeated)

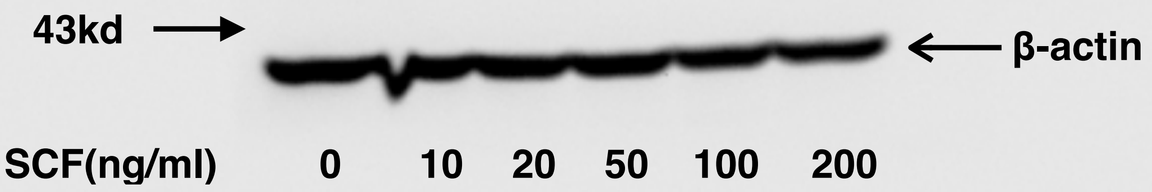

Figure. 2C p-ERK1/2 of time dependent experiment

Experiment 1 (Representative)

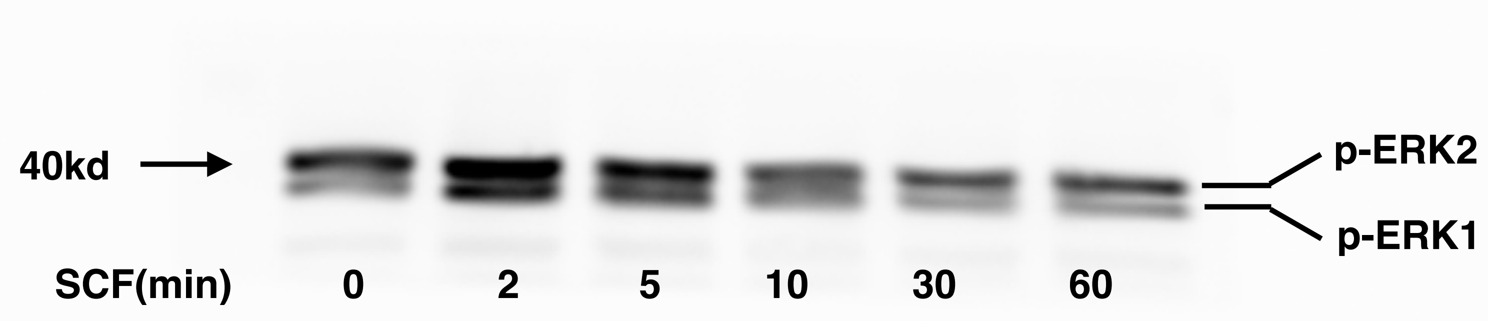

Figure. 2C p-ERK1/2 of time dependent experiment

Experiment 2 (Repeated)

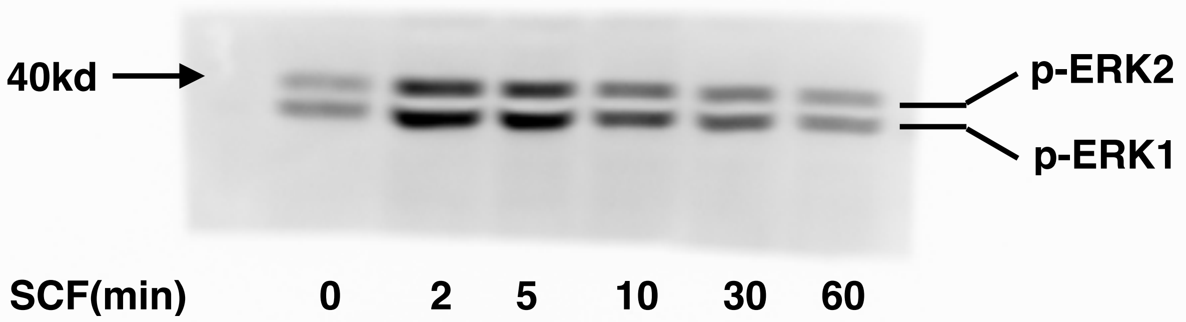

Figure. 2C p-ERK1/2 of time dependent experiment

Experiment 3 (Repeated)

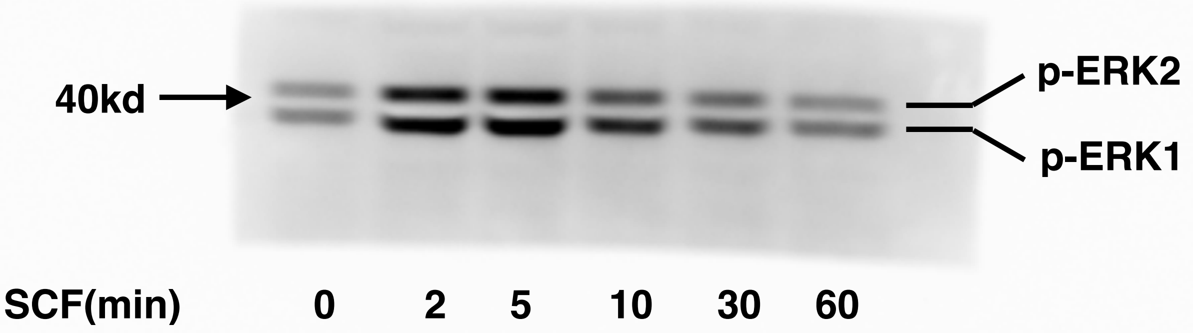

Figure. 2C ERK1/2 of time dependent experiment

Experiment 1 (Representative)

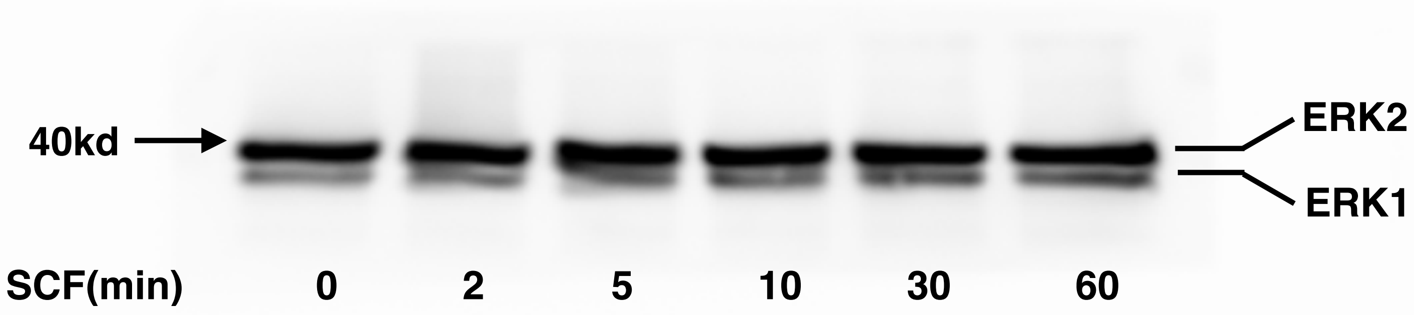

Figure. 2C ERK1/2 of time dependent experiment

Experiment 2 (Repeated)

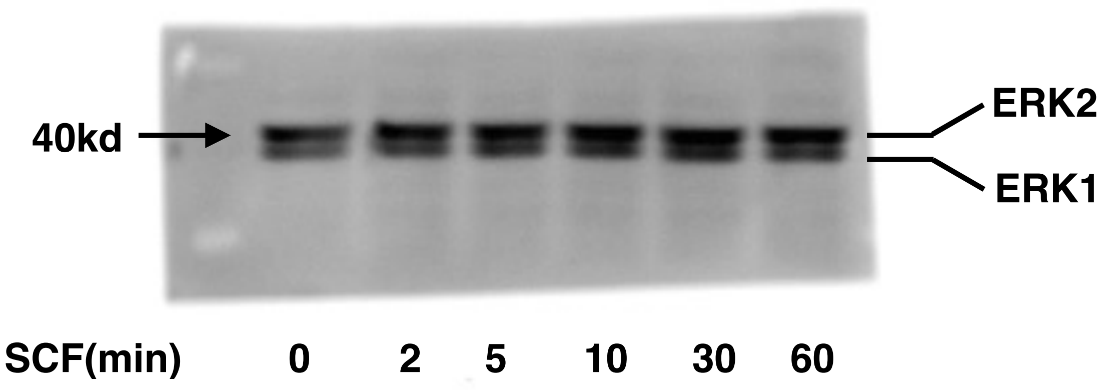

Figure. 2C ERK1/2 of time dependent experiment

Experiment 3 (Repeated)

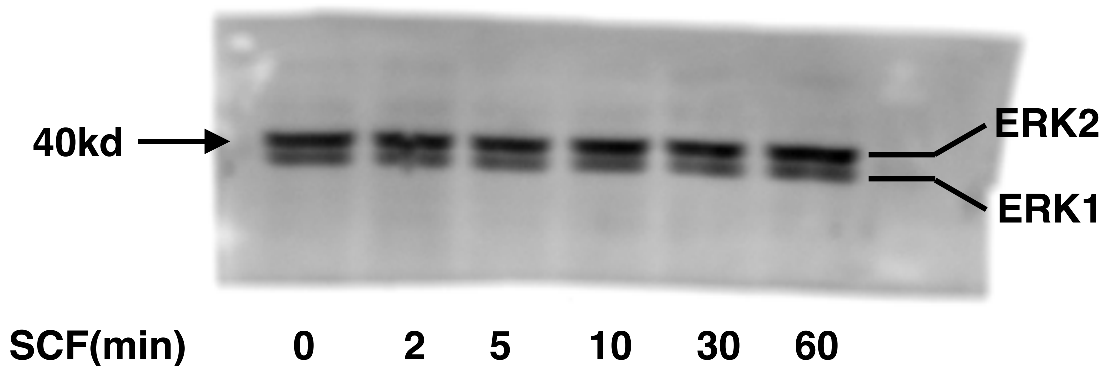

Figure. 2C p-p38 of time dependent experiment

Experiment 1 (Representative)

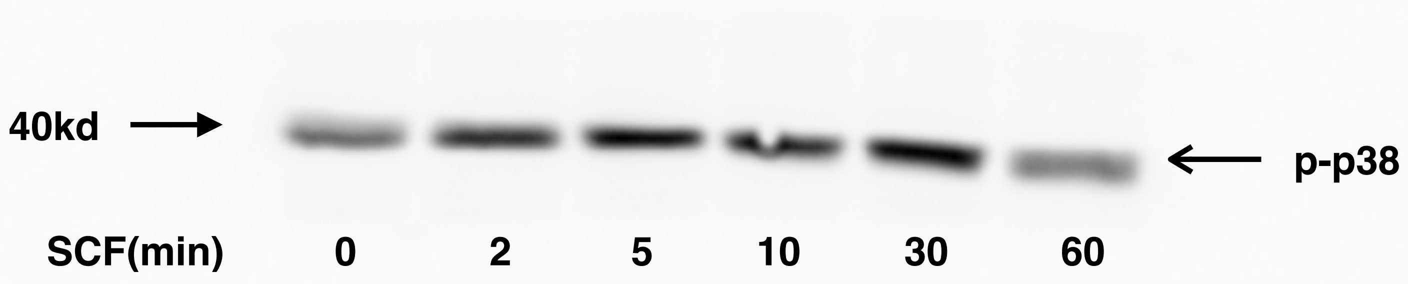

Figure. 2C p-p38 of time dependent experiment

Experiment 2 (Repeated)

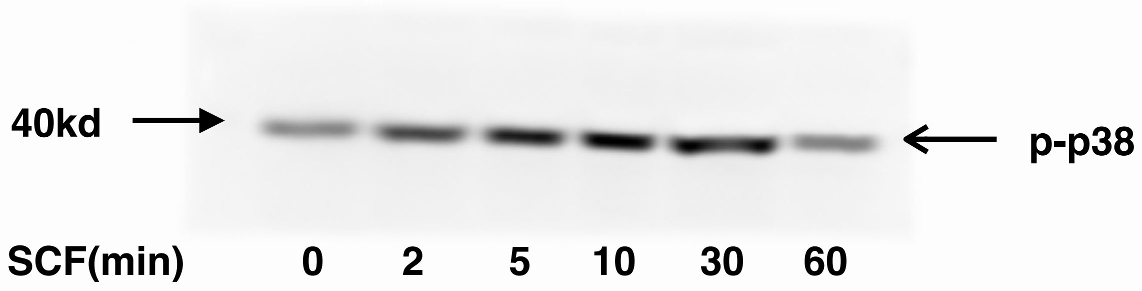

Figure. 2C p-p38 of time dependent experiment

Experiment 3 (Repeated)

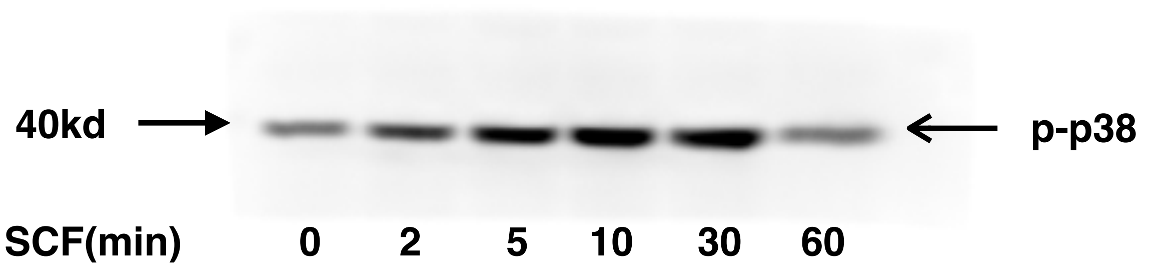

Figure. 2C p38 of time dependent experiment

Experiment 1 (Representative)

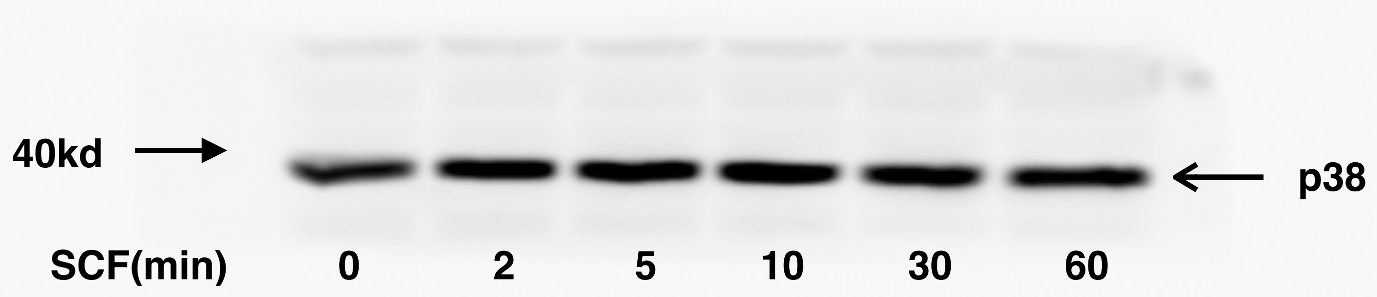

Figure. 2C p38 of time dependent experiment

Experiment 2 (Repeated)

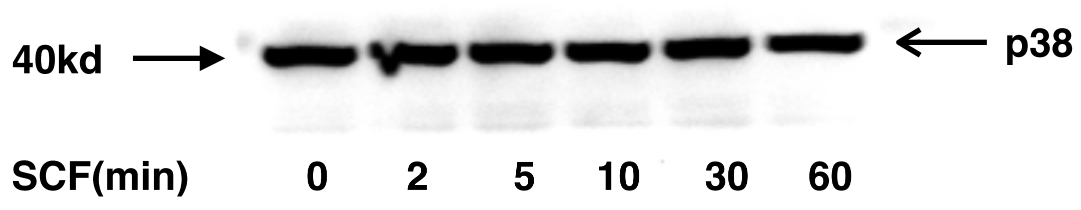

Figure. 2C p38 of time dependent experiment

Experiment 3 (Repeated)

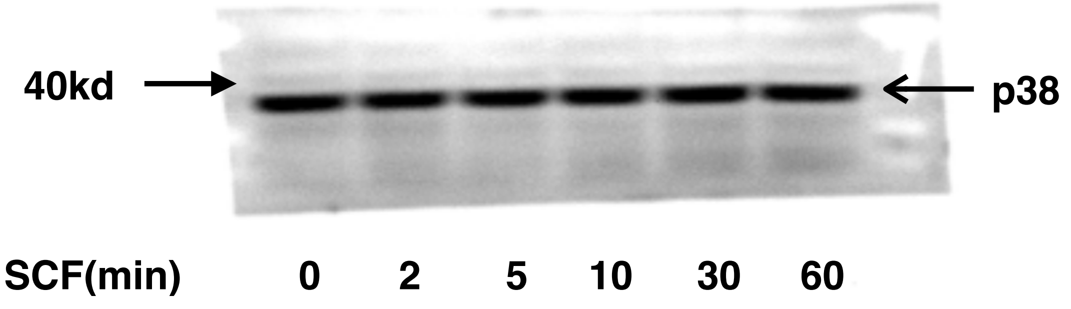

Figure. 2C β-actin of time dependent experiment

Experiment 1 (Representative)

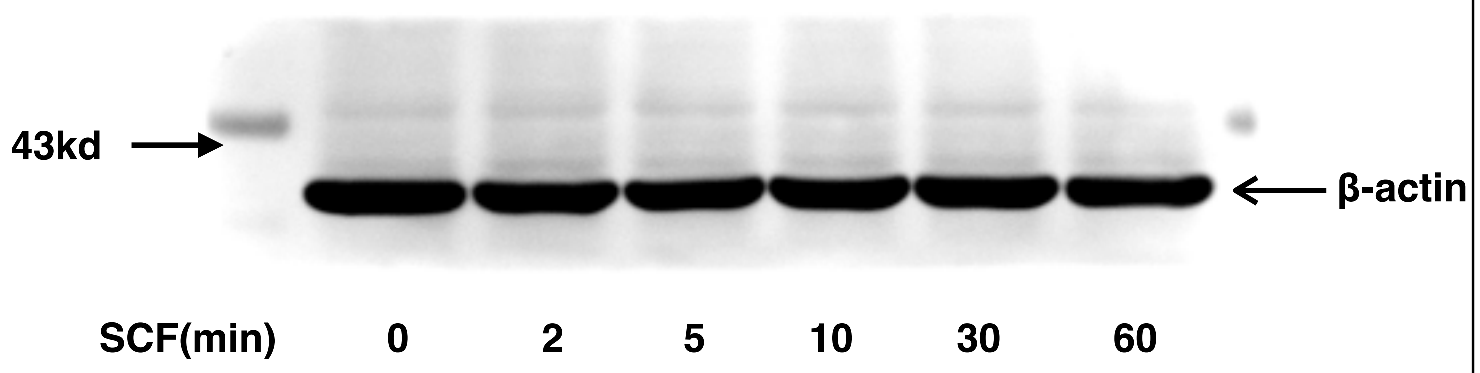

Figure. 2C β-actin of time dependent experiment

Experiment 2 (Repeated)

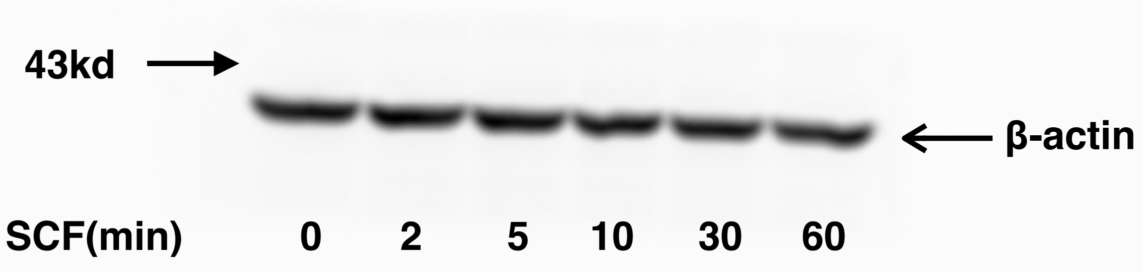

Figure. 2C β-actin of time dependent experiment

Experiment 3 (Repeated)

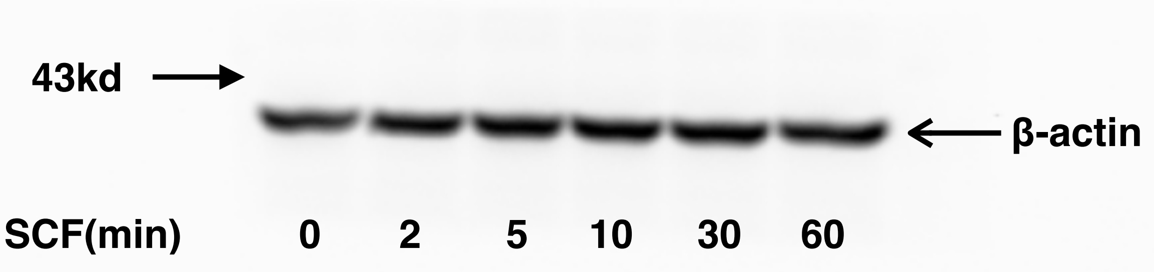

Figure. 3A p-p38 of Knockdown of CXCR4

Experiment 1 (Representative)

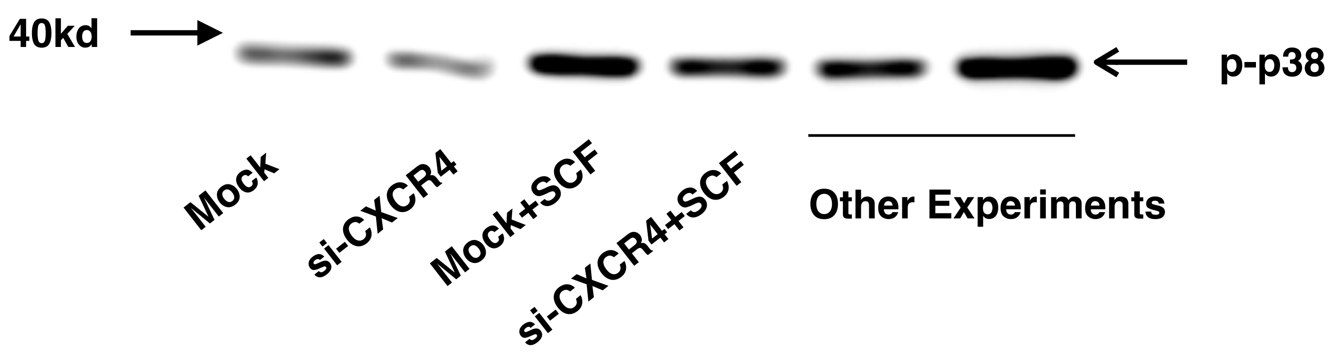

Figure. 3A p-p38 of Knockdown of CXCR4

Experiment 2 (Repeated)

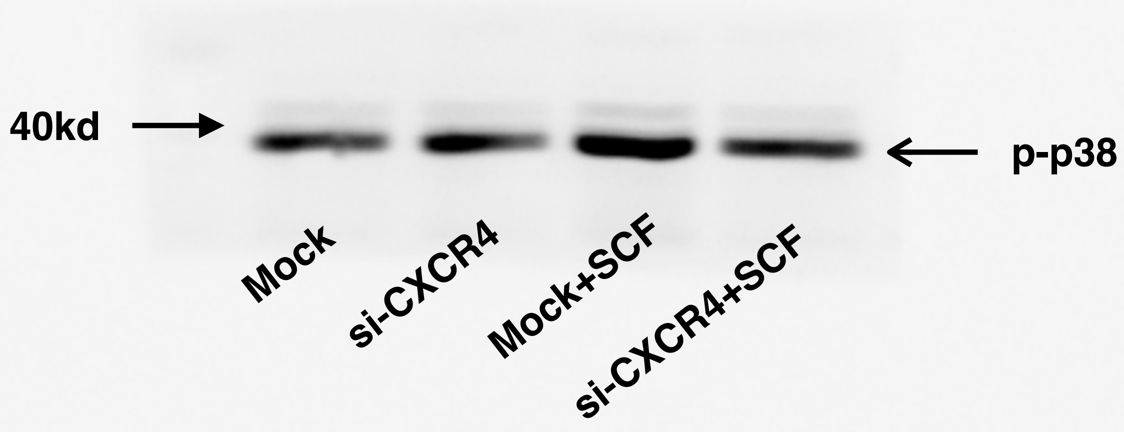

Figure. 3A p-p38 of Knockdown of CXCR4

Experiment 3 (Repeated)

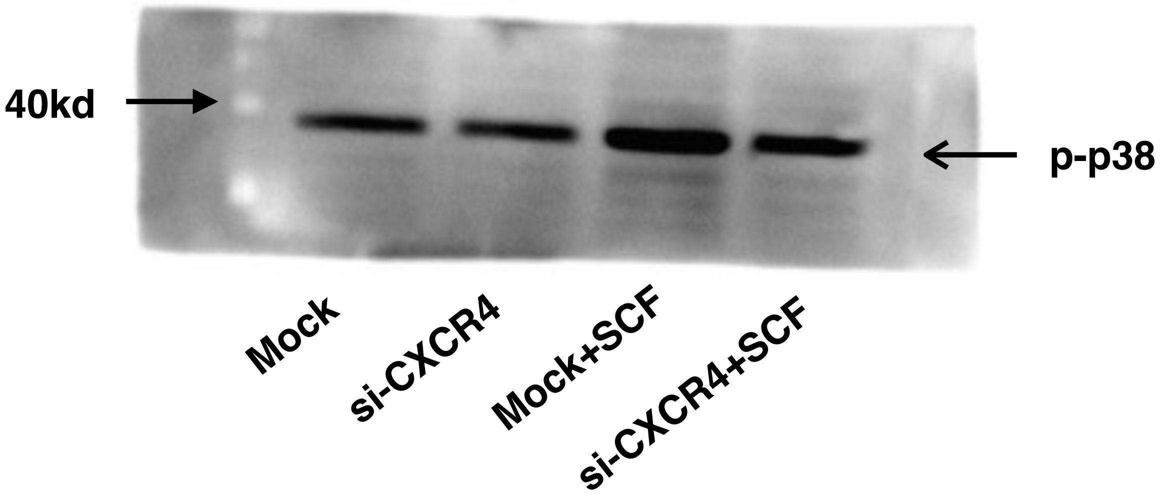

Figure. 3A p38 of Knockdown of CXCR4

Experiment 1 (Representative)

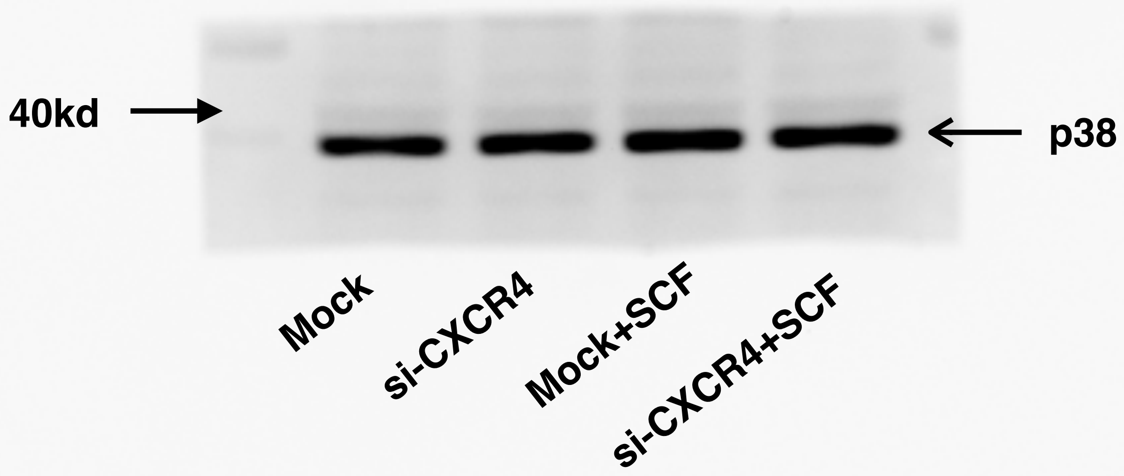

Figure. 3A p38 of Knockdown of CXCR4

Experiment 2 (Repeated)

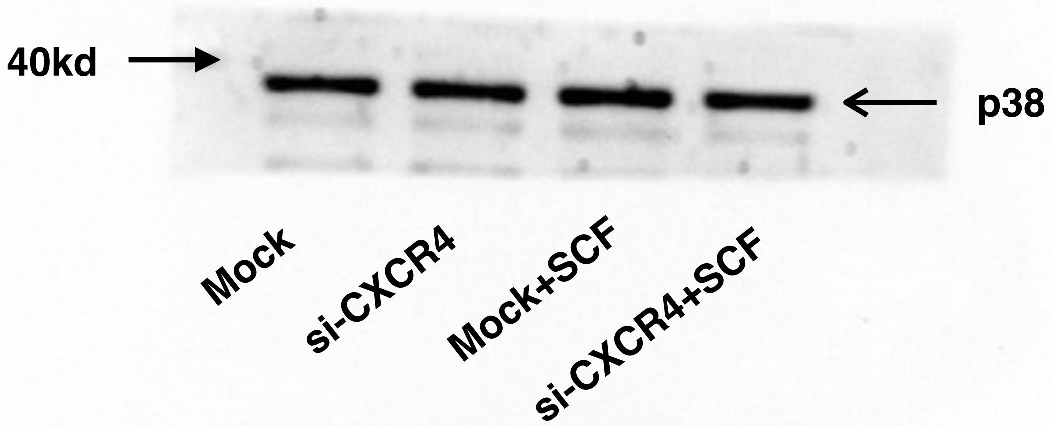

Figure. 3A p38 of Knockdown of CXCR4

Experiment 3 (Repeated)

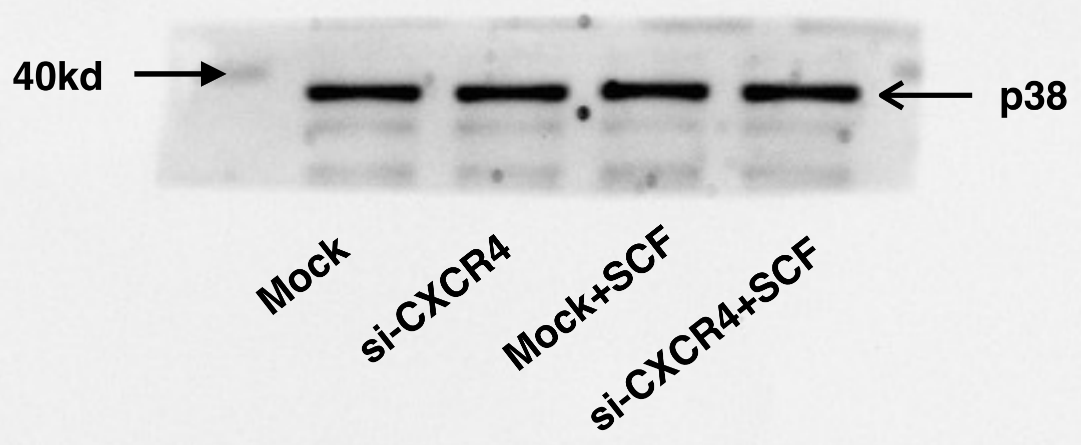

Figure. 3A p-ERK1/2 of knockdown of CXCR4

Experiment 1 (Representative)

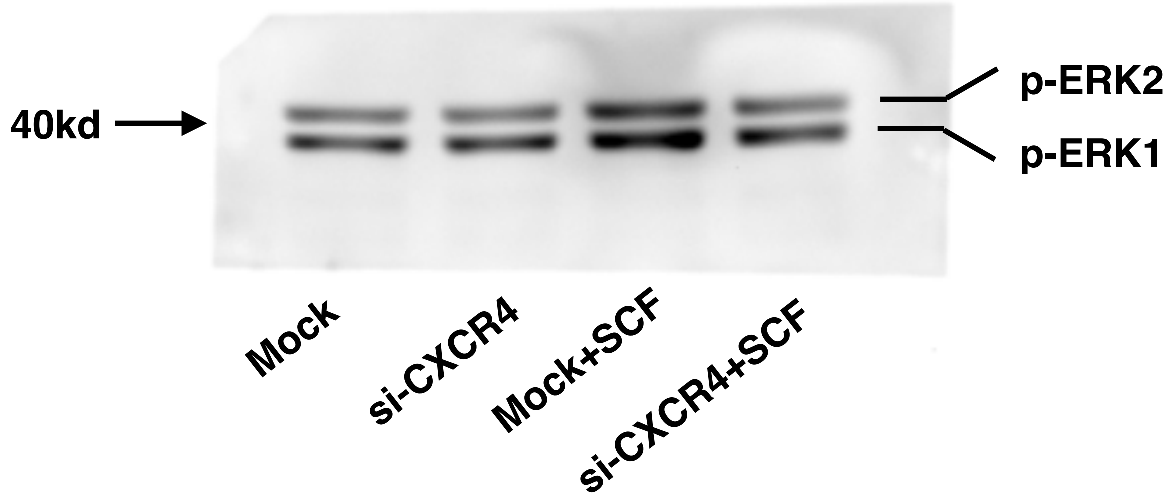

Figure. 3A p-ERK1/2 of knockdown of CXCR4

Experiment 2 (Repeated)

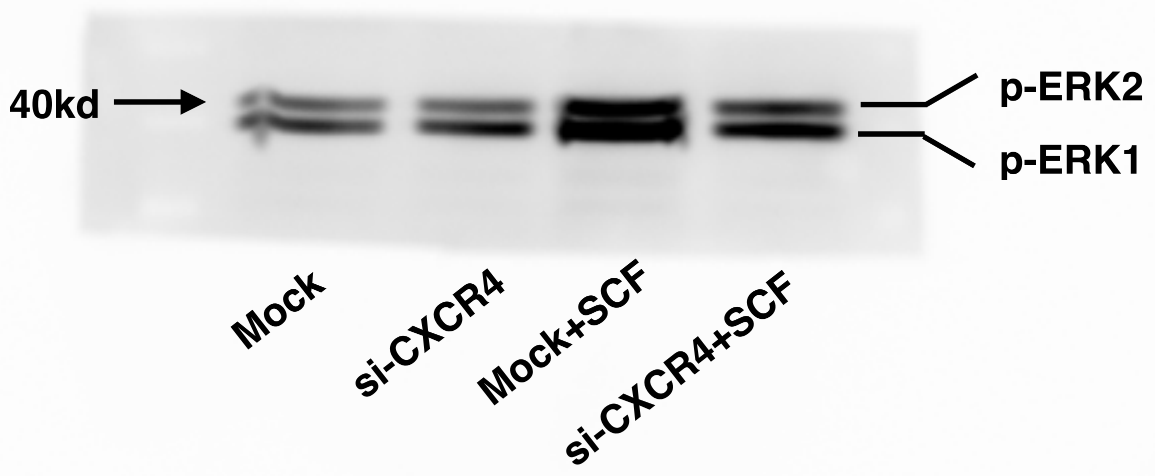

Figure. 3A p-ERK1/2 of knockdown of CXCR4

Experiment 3 (Repeated)

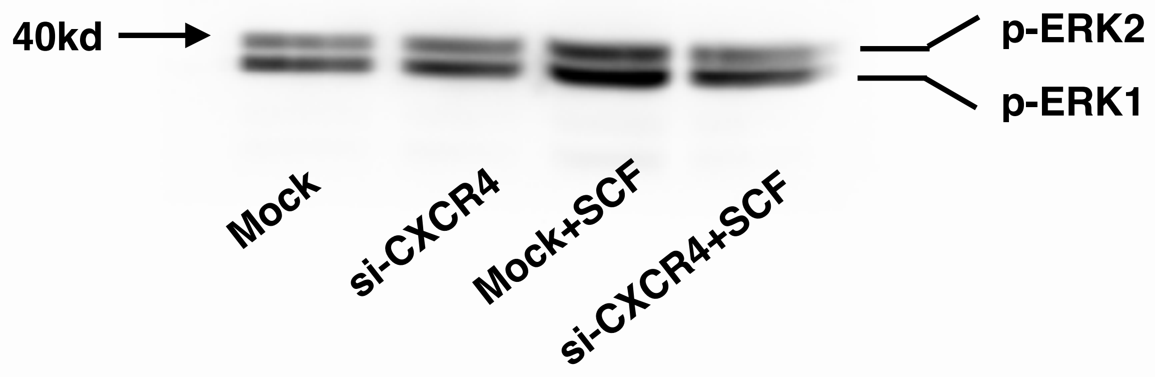

Figure. 3A ERK1/2 of Knockdown of CXCR4

Experiment 1 (Representative)

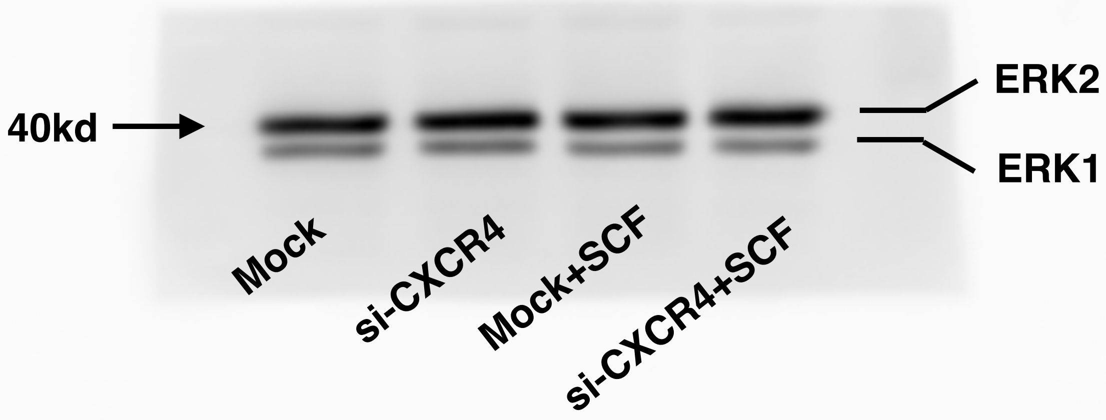

Figure. 3A ERK1/2 of Knockdown of CXCR4

Experiment 2 (Repeated)

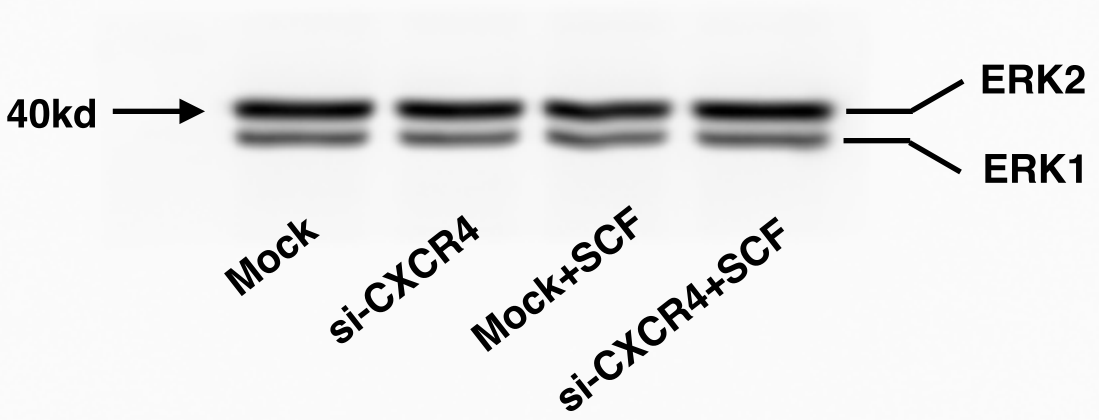

Figure. 3A ERK1/2 of Knockdown of CXCR4

Experiment 3 (Repeated)

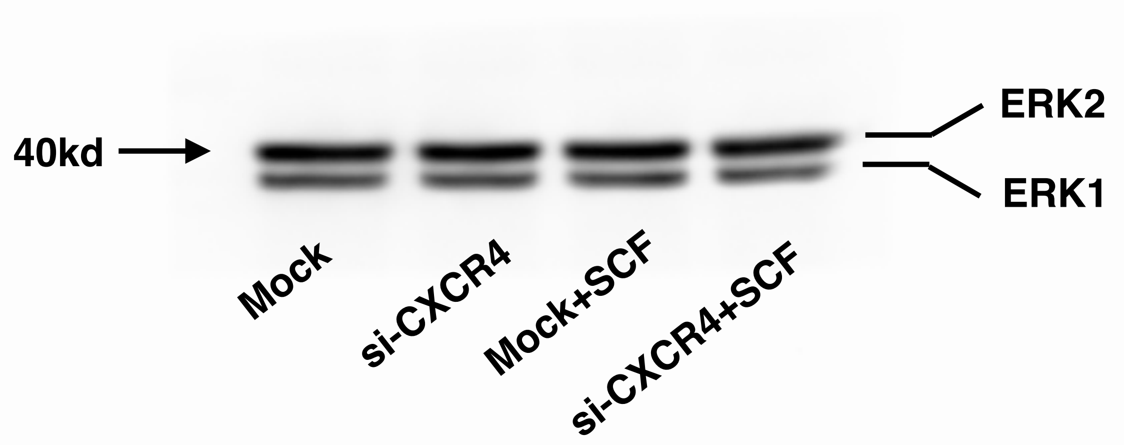

Figure. 3A Knockdown of CXCR4   Experiment 1 (Representative)

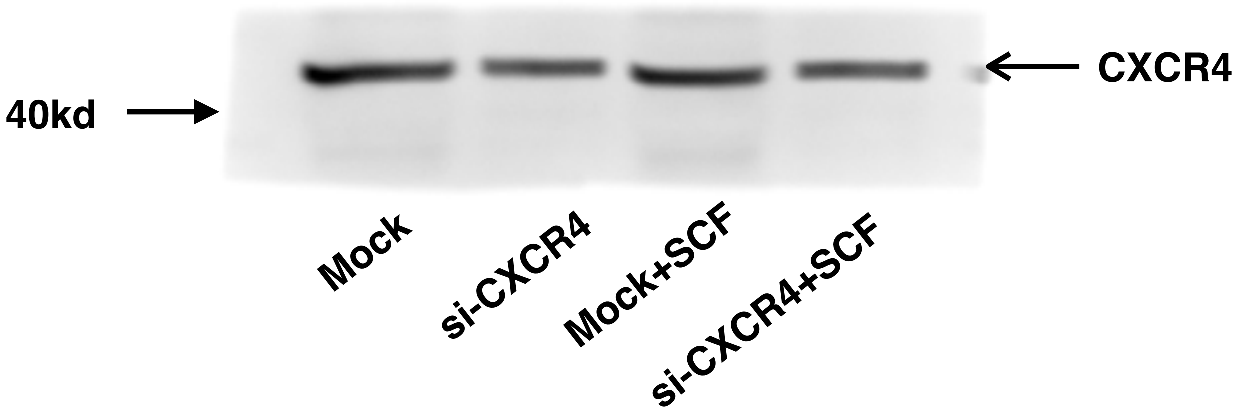

Figure. 3A Knockdown of CXCR4   Experiment 2 (Repeated)

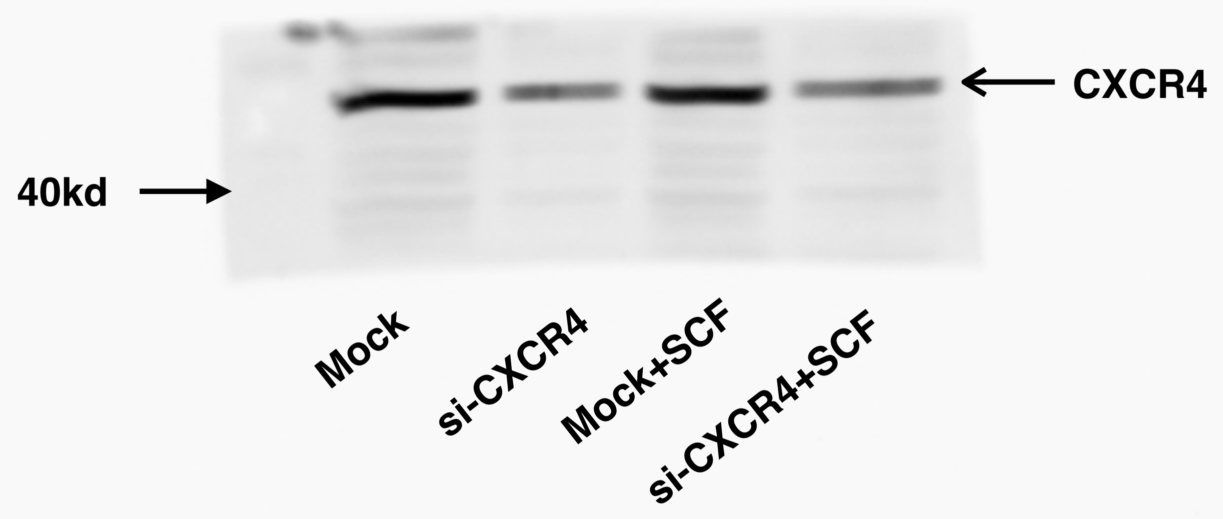

Figure. 3A Knockdown of CXCR4   Experiment 3 (Repeated)

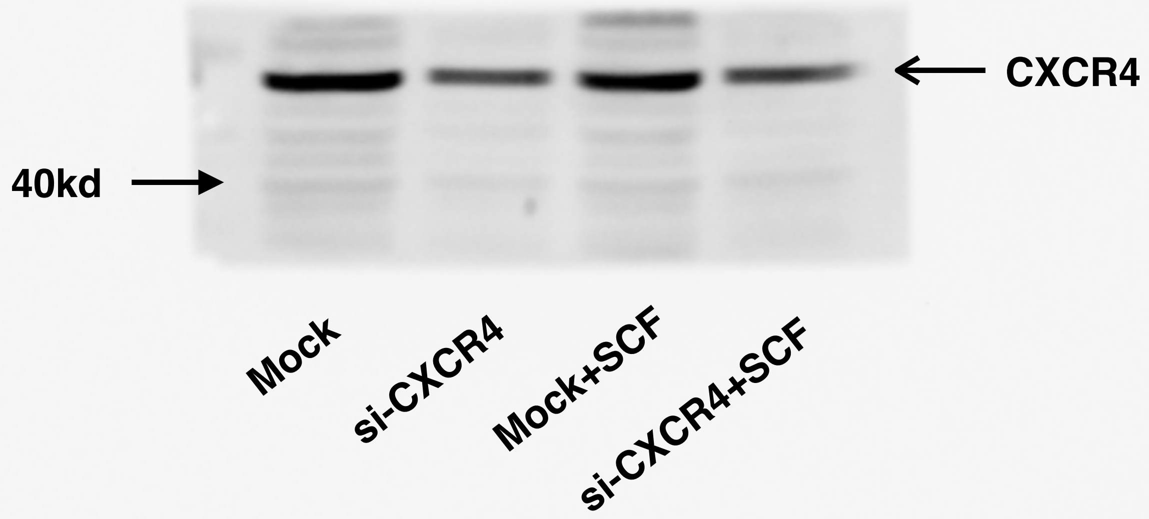

Figure. 3A β-actin Knockdown of CXCR4

Experiment 1 (Representative)

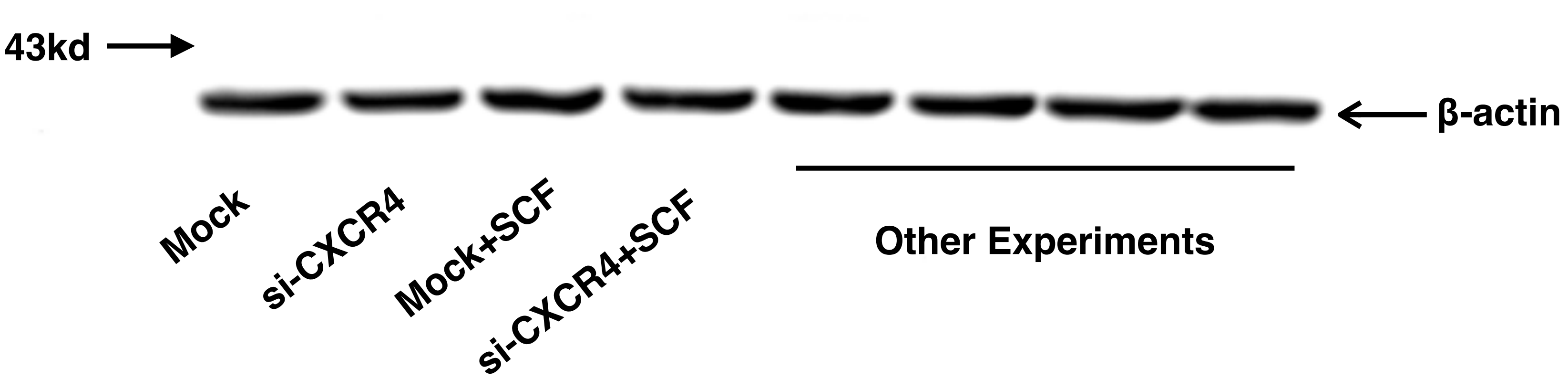

Figure. 3A β-actin Knockdown of CXCR4

Experiment 2 (Repeated)

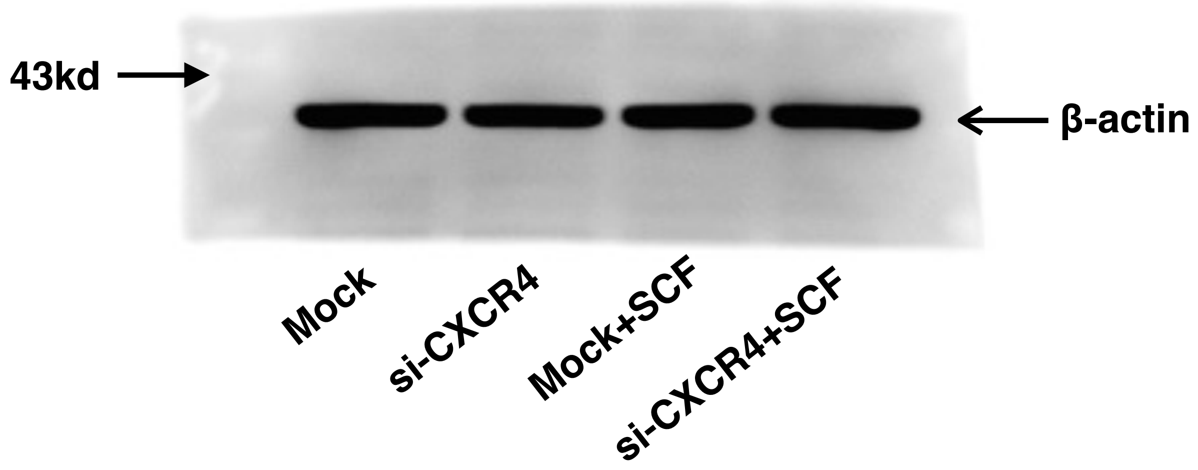

Figure. 3A β-actin Knockdown of CXCR4

Experiment 3 (Repeated)

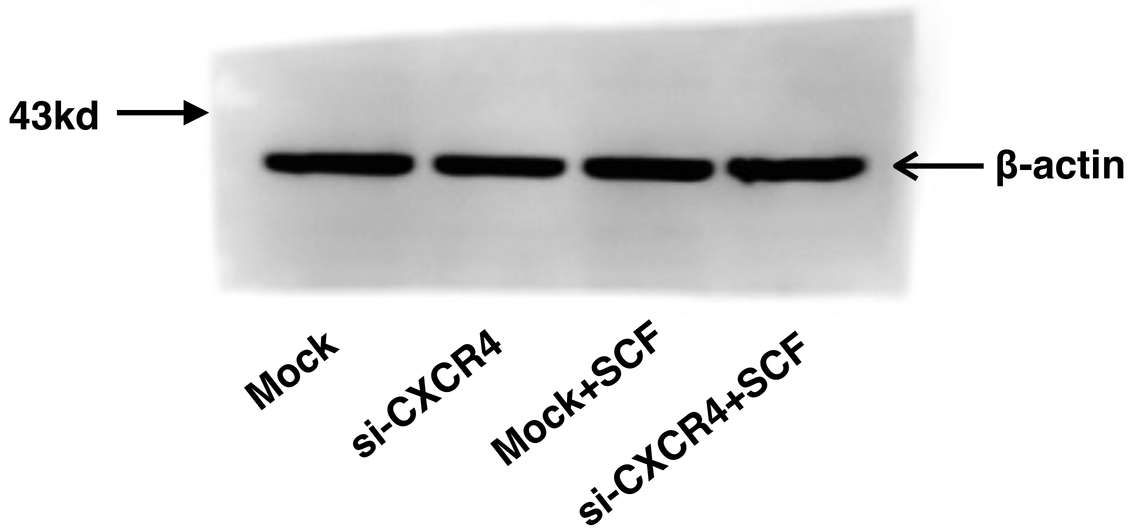

Figure. 4A p-CXCR4 of dose dependent experiment

Experiment 1 (Representative)

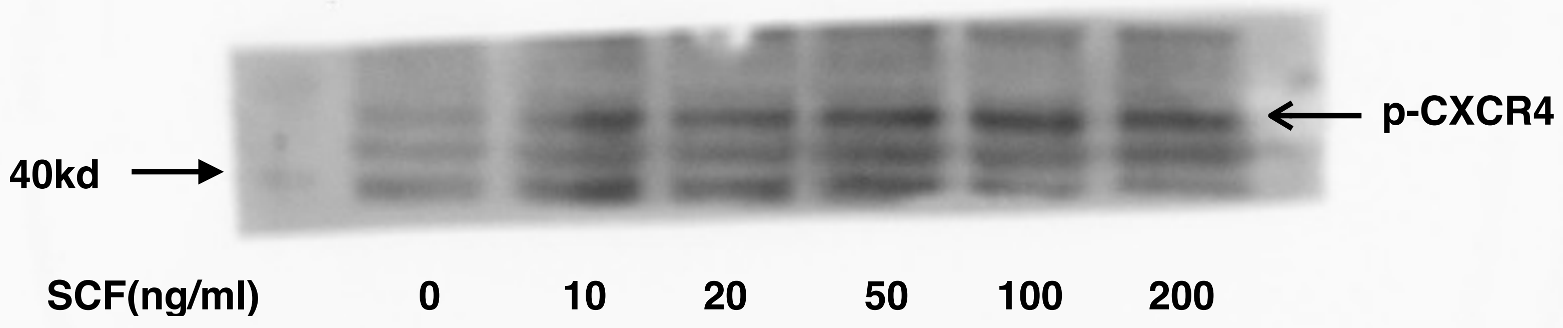

Figure. 4A p-CXCR4 of dose dependent experiment

Experiment 2 (Repeated)

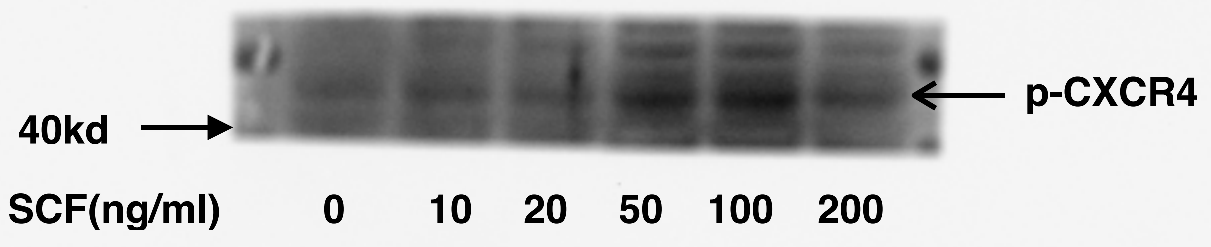

Figure. 4A p-CXCR4 of dose dependent experiment

Experiment 3 (Repeated)

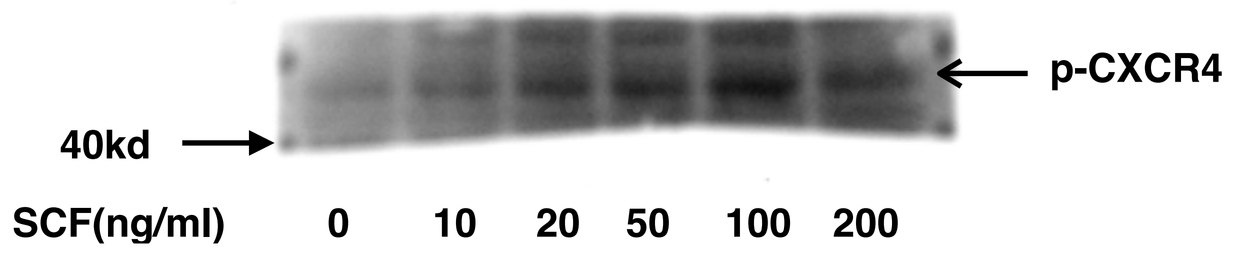

Figure. 4A CXCR4 of dose dependent experiment

Experiment 1 (Representative)

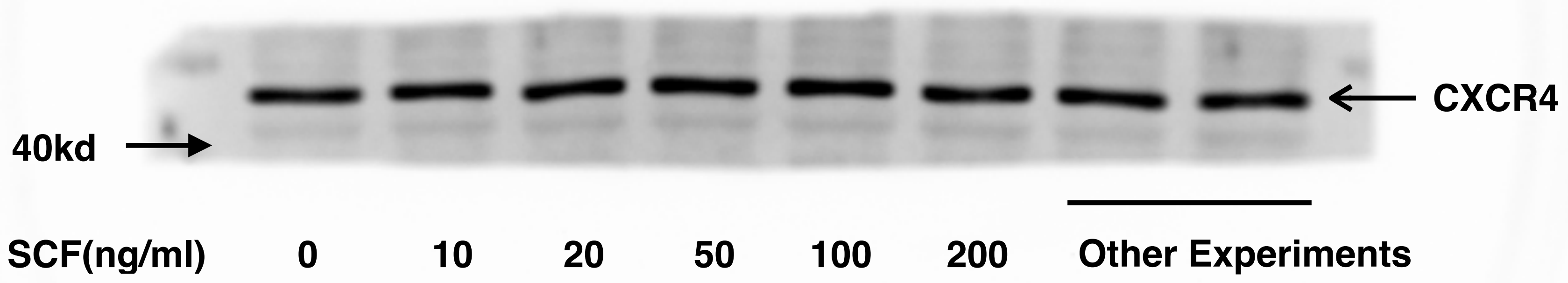

Figure. 4A CXCR4 of dose dependent experiment

Experiment 2 (Repeated)

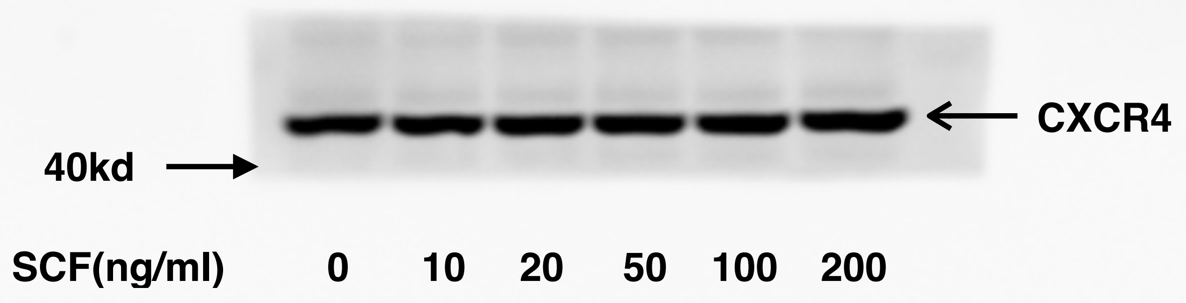

Figure. 4A CXCR4 of dose dependent experiment

Experiment 3 (Repeated)

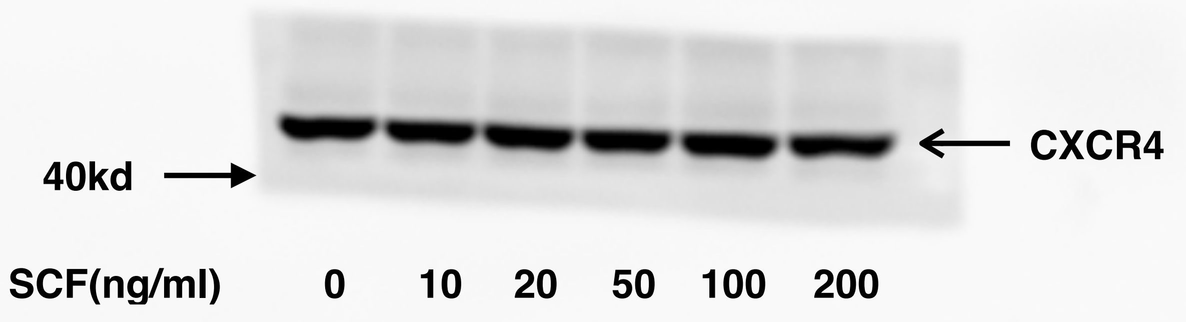

Figure. 4A p-CXCR4 of time dependent experiment

Experiment 1 (Representative)

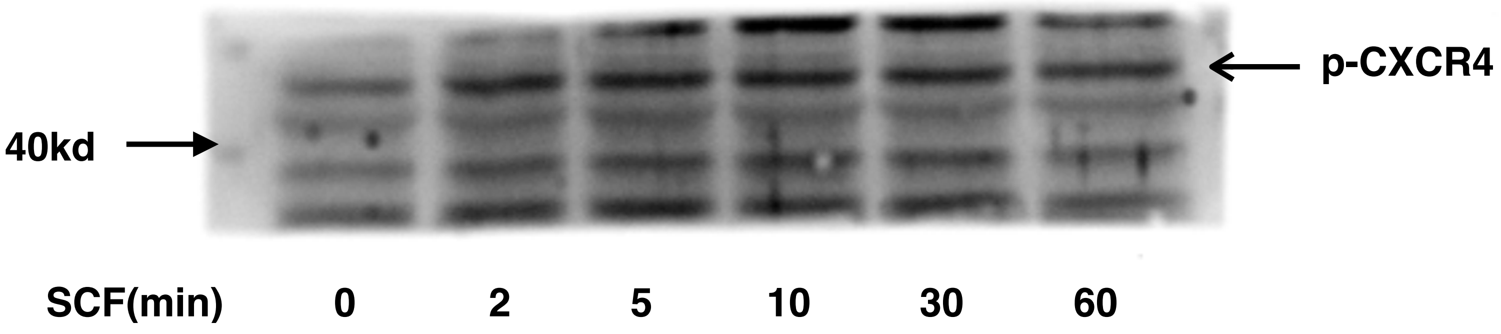

Figure. 4A p-CXCR4 of time dependent experiment

Experiment 2 (Repeated)

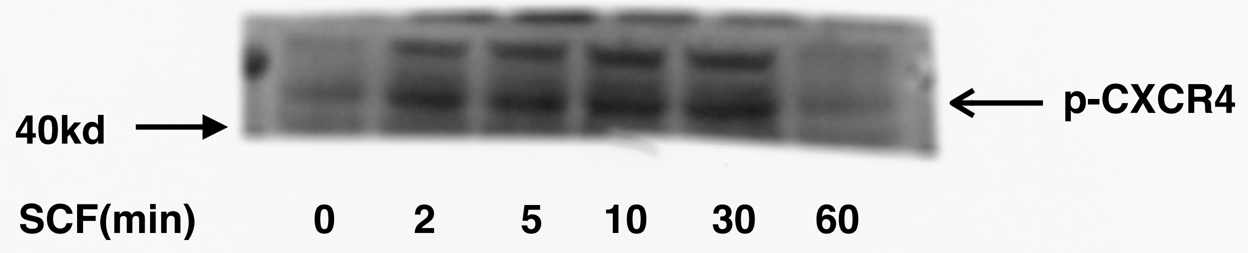

Figure. 4A p-CXCR4 of time dependent experiment

Experiment 3 (Repeated)

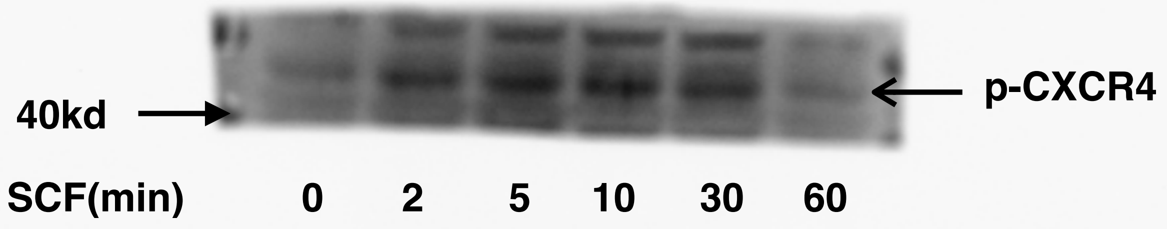

Figure. 4A CXCR4 of time dependent experiment

Experiment 1 (Representative)

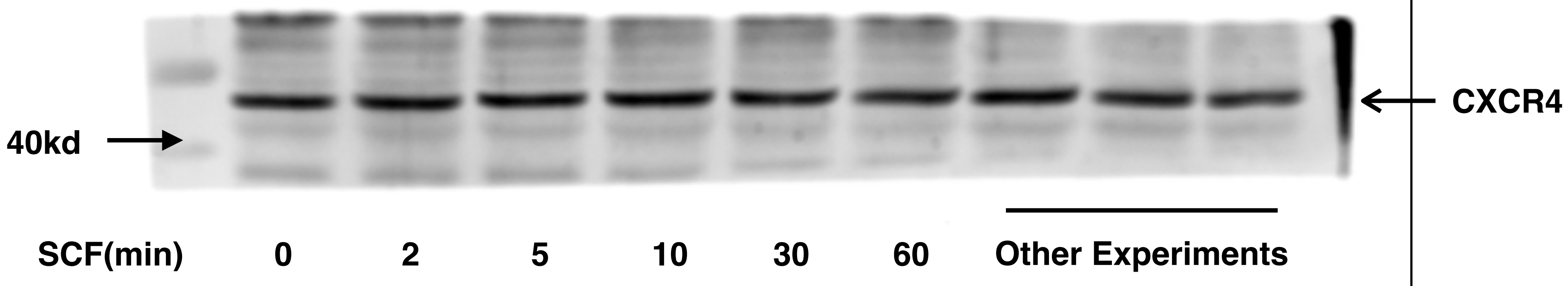

Figure. 4A CXCR4 of time dependent experiment

Experiment 2 (Repeated)

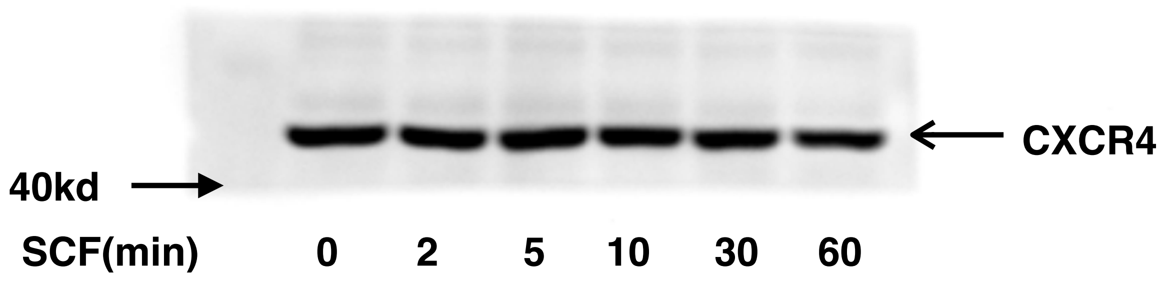

Figure. 4A CXCR4 of time dependent experiment

Experiment 3 (Repeated)

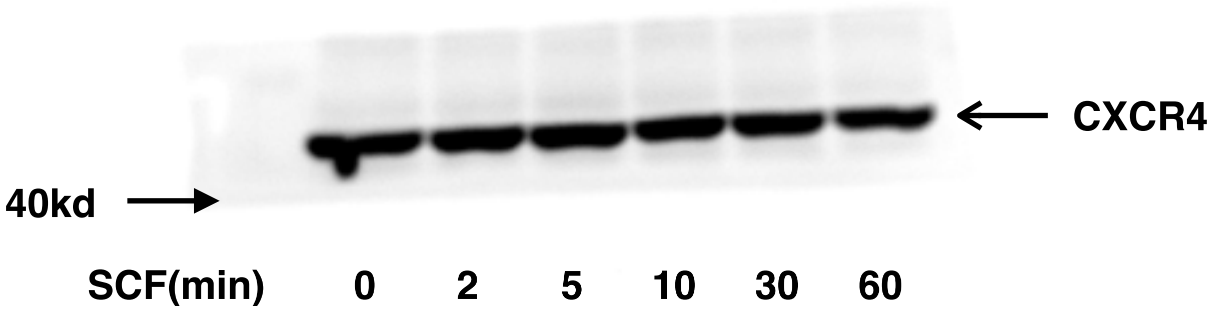

Figure. 4B p-CXCR4 of c-kit blocking

Experiment 1 (Representative)

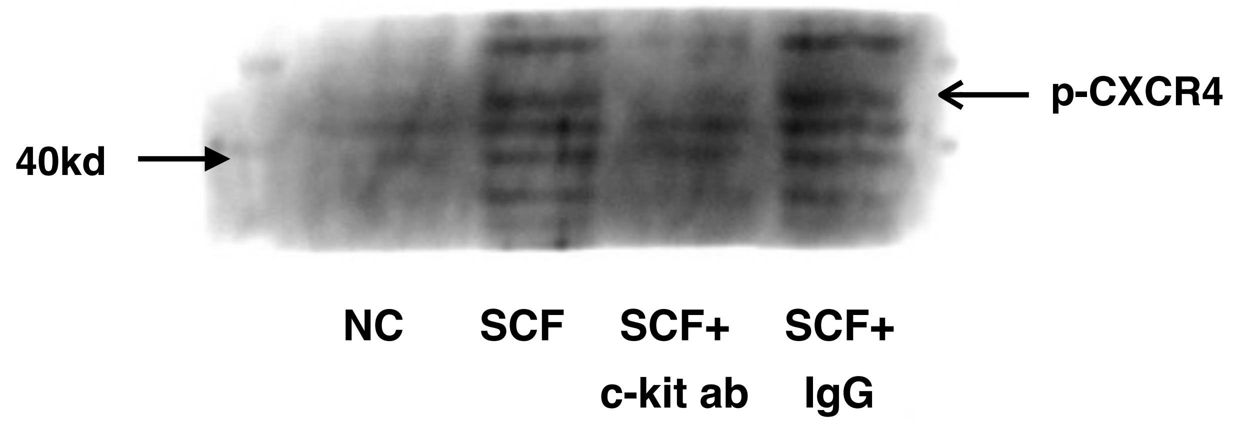

Figure. 4B p-CXCR4 of c-kit blocking

Experiment 2 (Repeated)

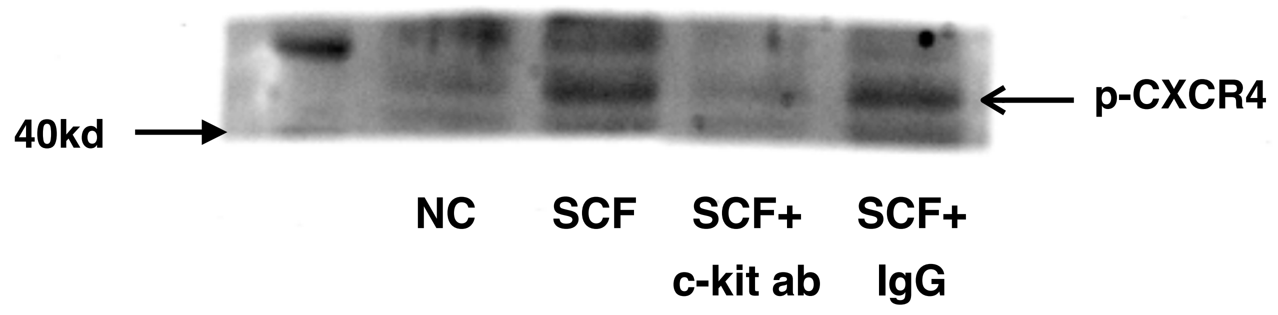

Figure. 4B p-CXCR4 of c-kit blocking

Experiment 3 (Repeated)

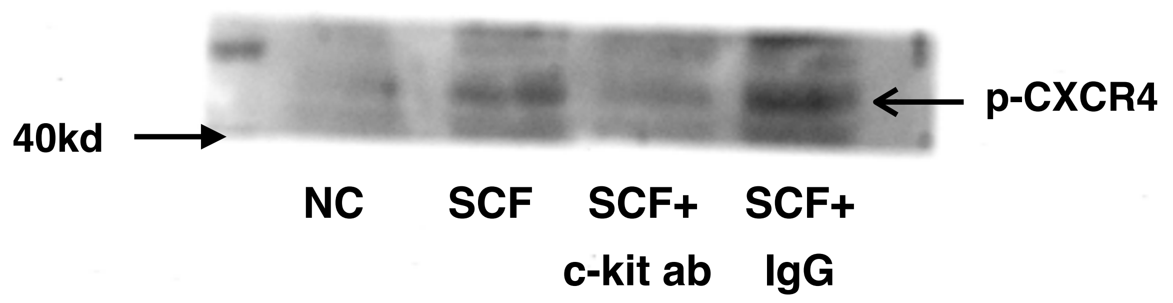

Figure. 4B CXCR4 of c-kit blocking

Experiment 1 (Representative)

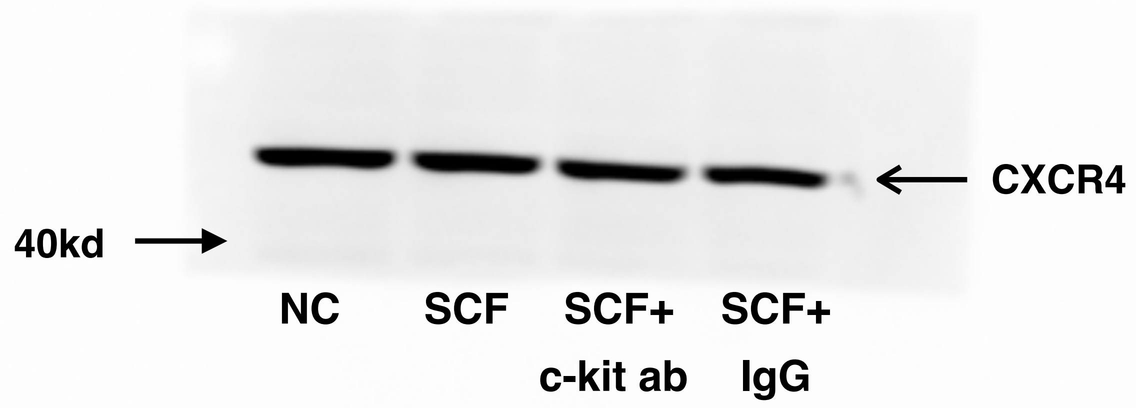

Figure. 4B CXCR4 of c-kit blocking

Experiment 2 (Repeated)

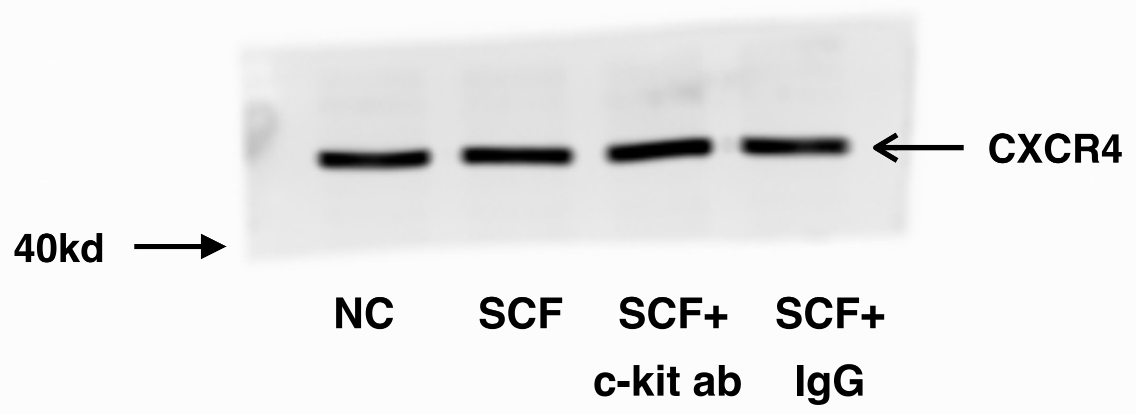

Figure. 4B CXCR4 of c-kit blocking

Experiment 3 (Repeated)

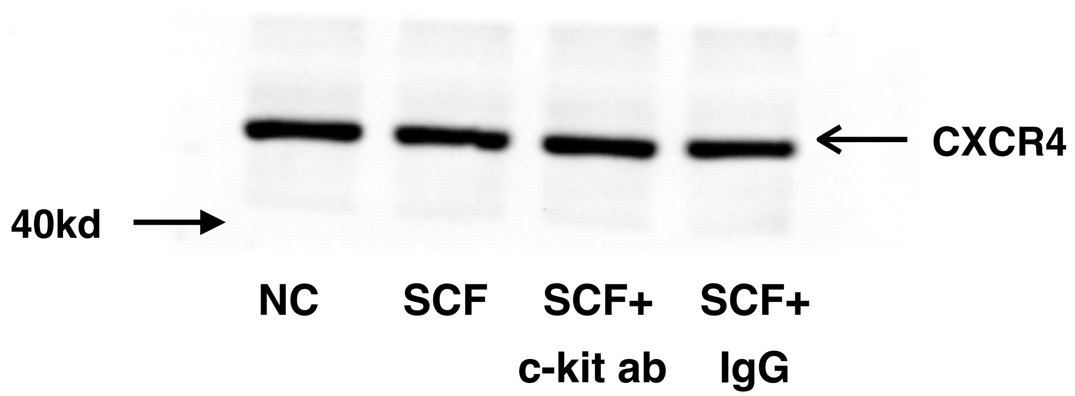

Figure. 4B p-p38 of c-kit blocking

Experiment 1 (Representative)

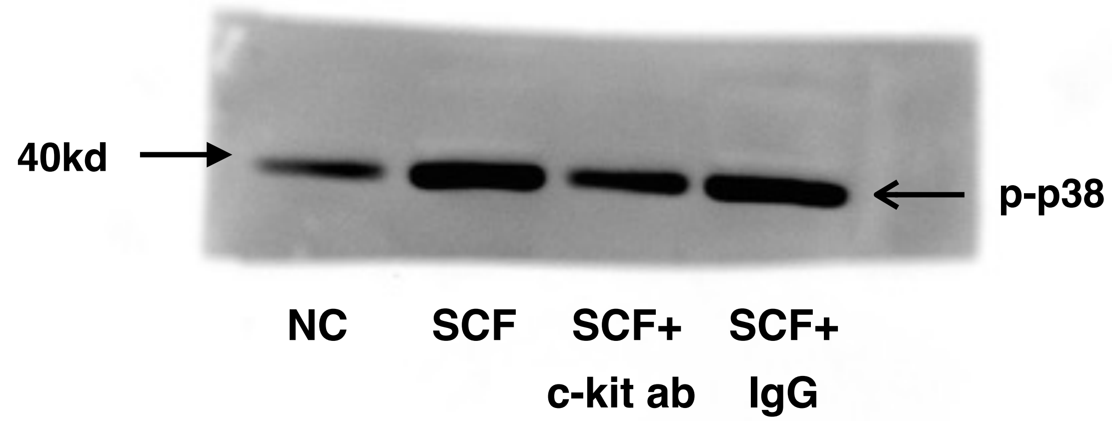

Figure. 4B p-p38 of c-kit blocking

Experiment 2 (Repeated)

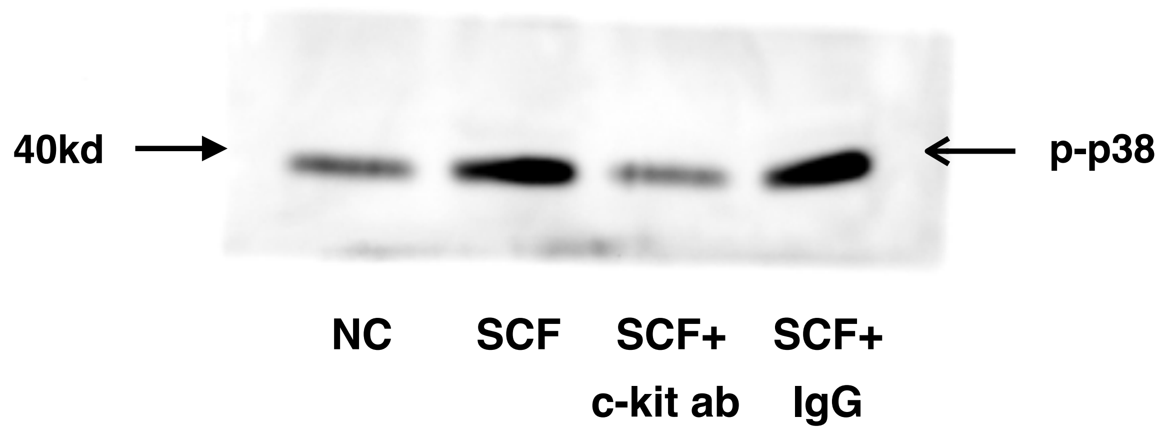

Figure. 4B p-p38 of c-kit blocking

Experiment 3 (Repeated)

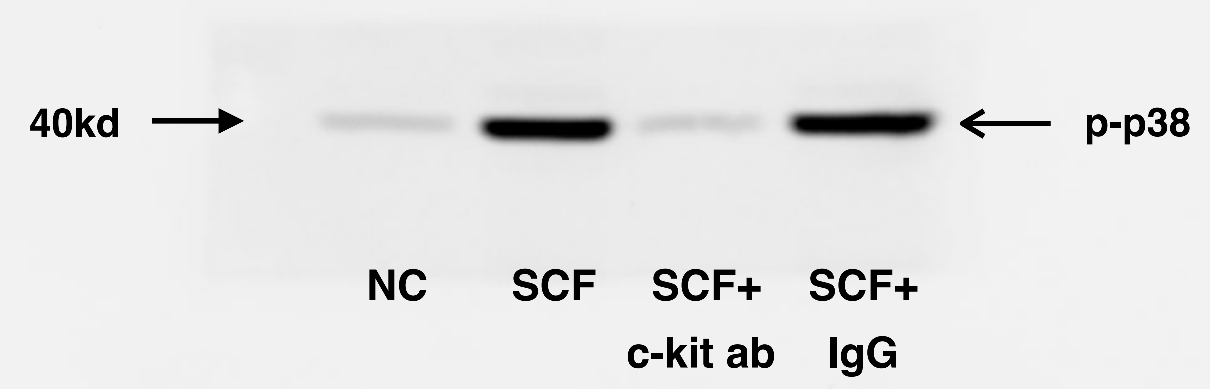

Figure. 4B p38 of c-kit blocking

Experiment 1 (Representative)

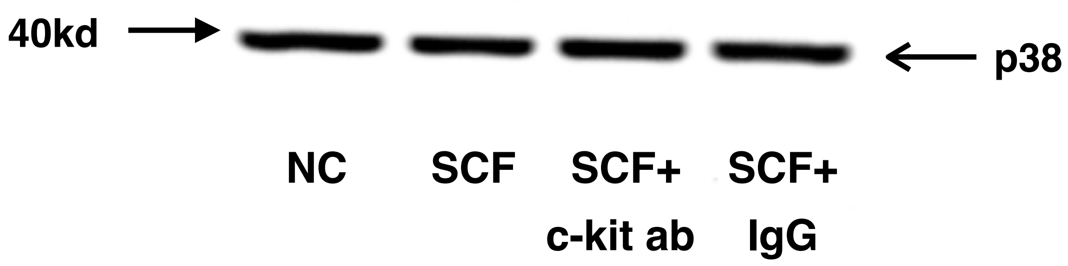

Figure. 4B p38 of c-kit blocking

Experiment 2 (Repeated)

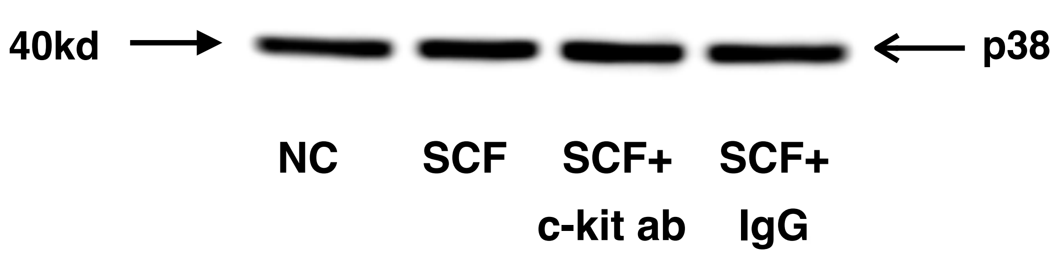

Figure. 4B p38 of c-kit blocking

Experiment 3 (Repeated)

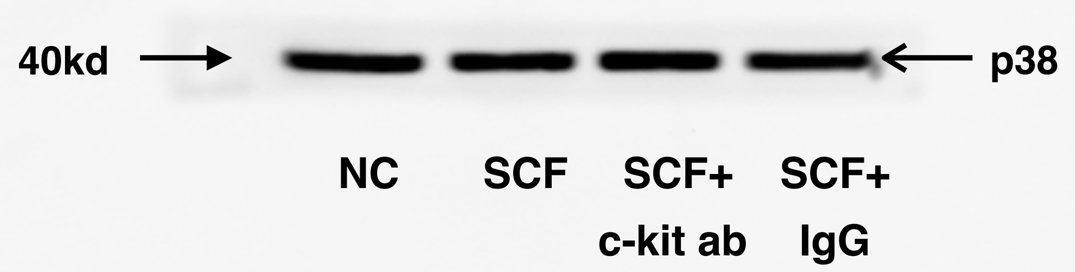

Figure. 4B p-ERK1/2 of c-kit blocking

Experiment 1 (Representative)

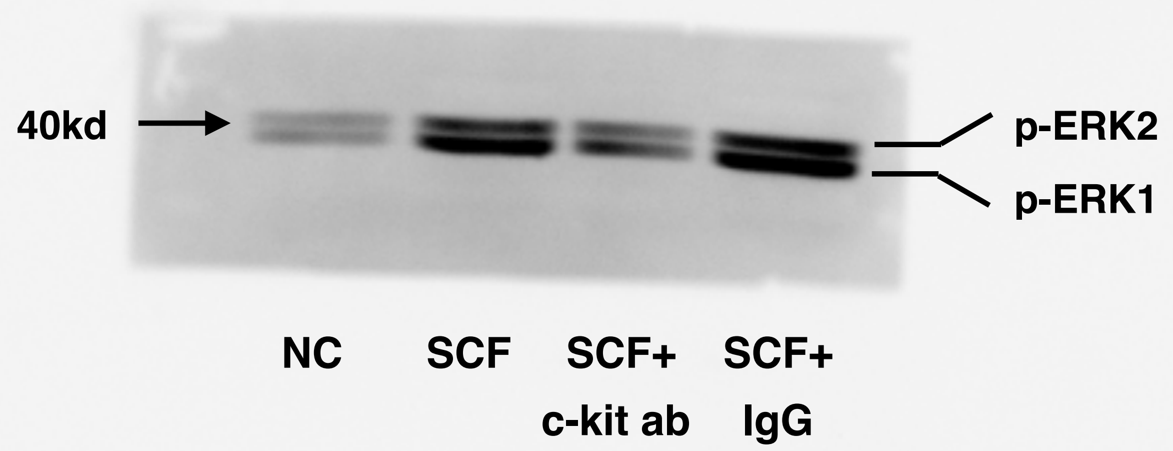

Figure. 4B p-ERK1/2 of c-kit blocking

Experiment 2 (Repeated)

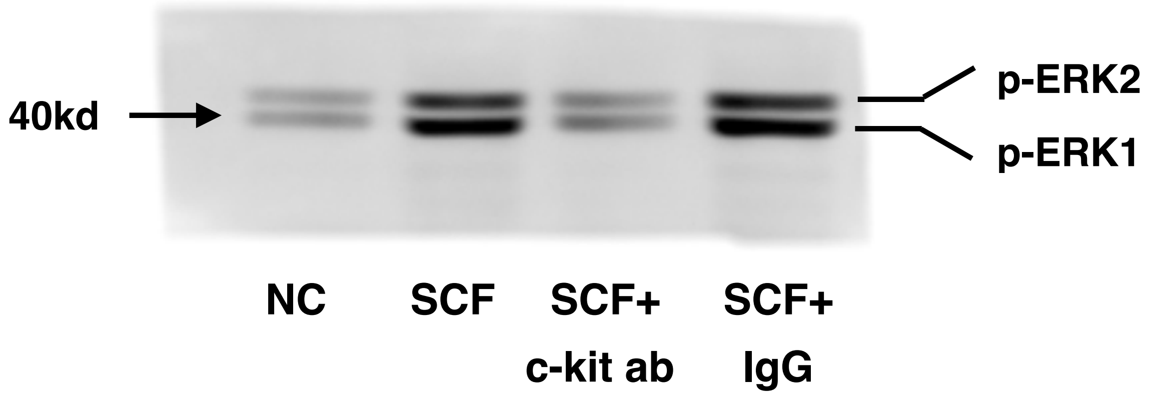

Figure. 4B p-ERK1/2 of c-kit blocking

Experiment 3 (Repeated)

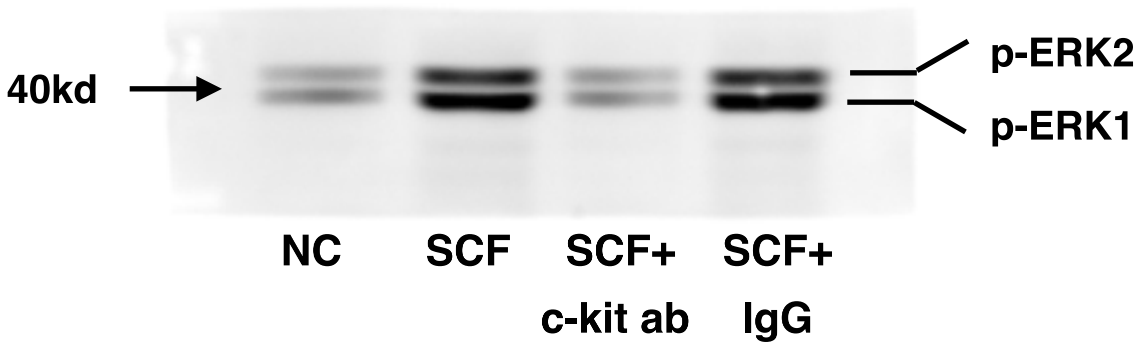

Figure. 4B ERK1/2 of c-kit blocking

Experiment 1 (Representative)

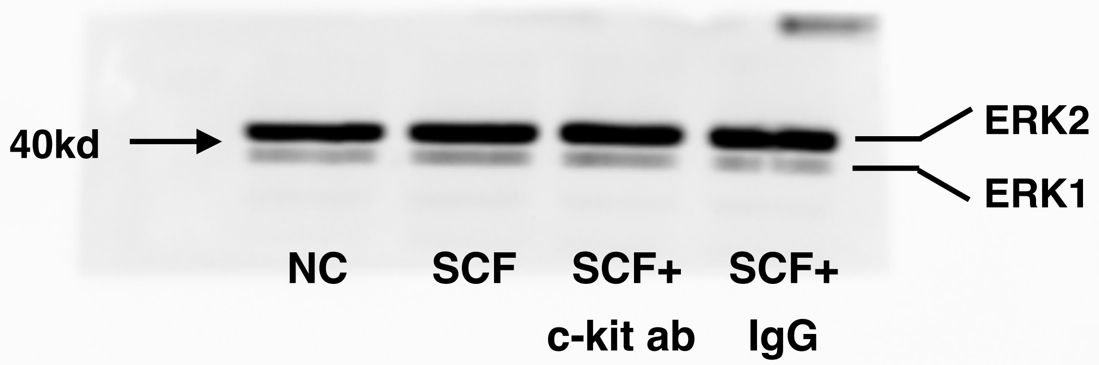

Figure. 4B ERK1/2 of c-kit blocking

Experiment 2 (Repeated)

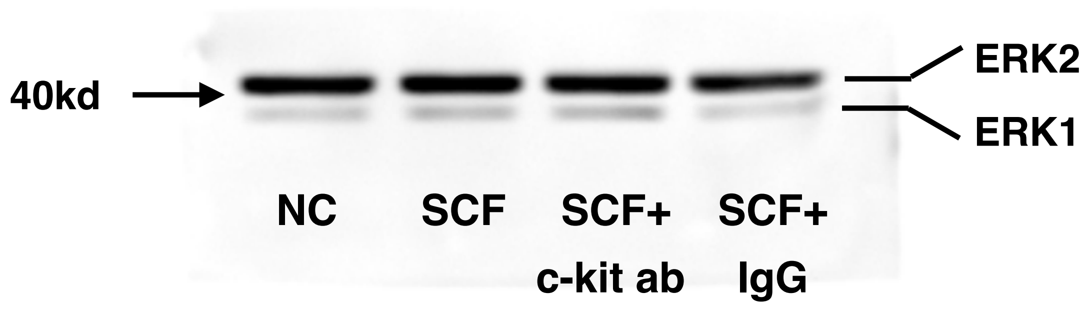

Figure. 4B ERK1/2 of c-kit blocking

Experiment 3 (Repeated)

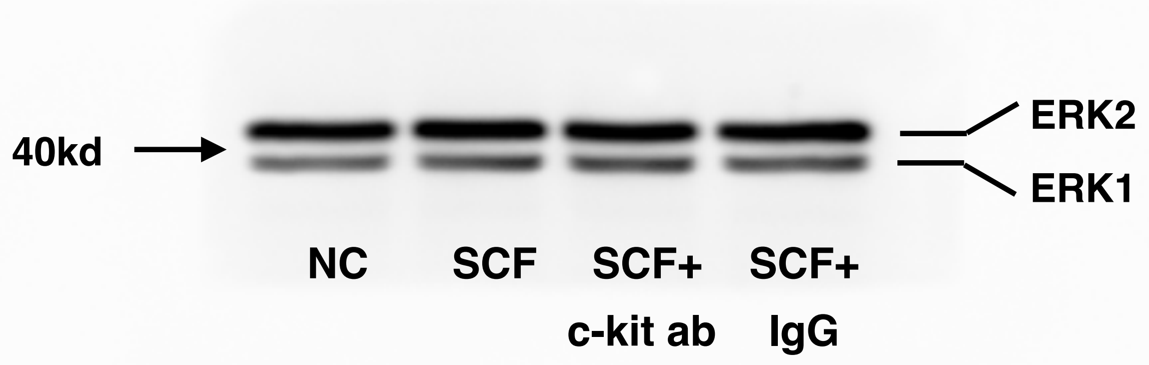

Figure. 4B β-actin of c-kit blocking

Experiment 1 (Representative)

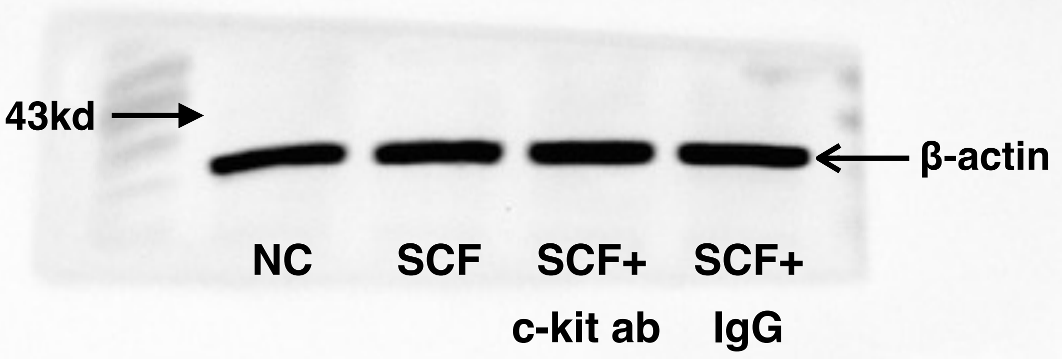

Figure. 4B β-actin of c-kit blocking

Experiment 2 (Repeated)

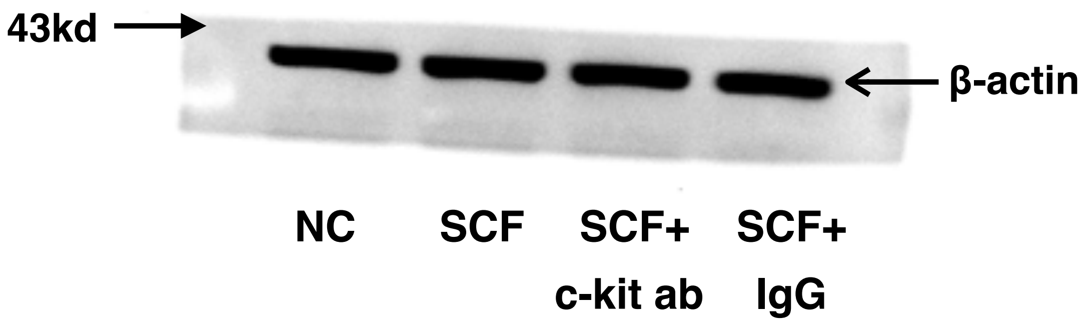

Figure. 4B β-actin of c-kit blocking

Experiment 3 (Repeated)

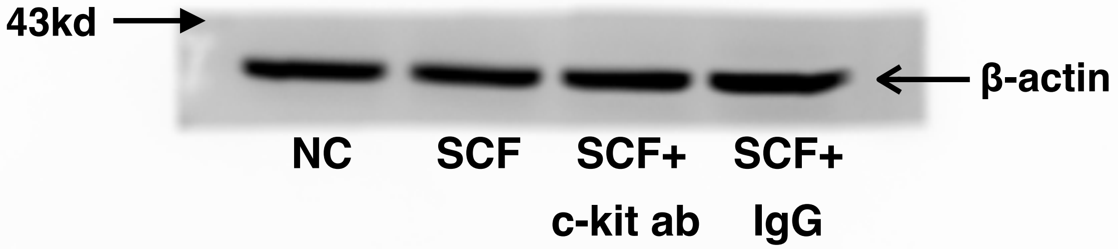

Figure. 6A p-CXCR4 of Knockdown of GRK2 and GRK6

Experiment 1 (Representative)

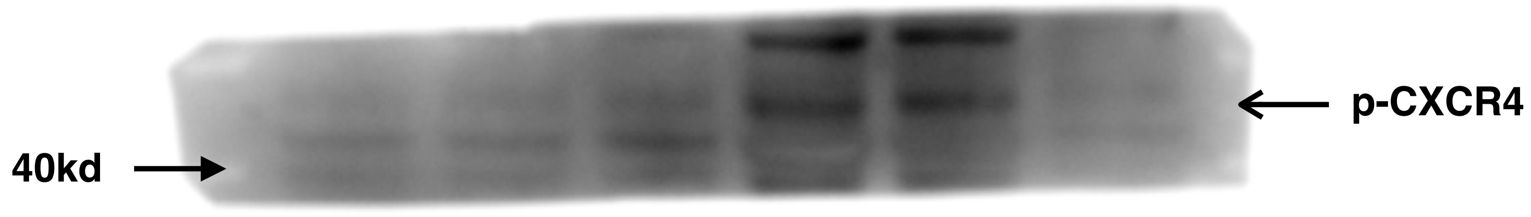

SCF	-	-	-	+	+	+
si-GRK2	-	+	-	-	+	-
si-GRK6	-	-	+	-	-	+

Figure. 6A p-CXCR4 of Knockdown of GRK2 and GRK6

Experiment 2 (Repeated)

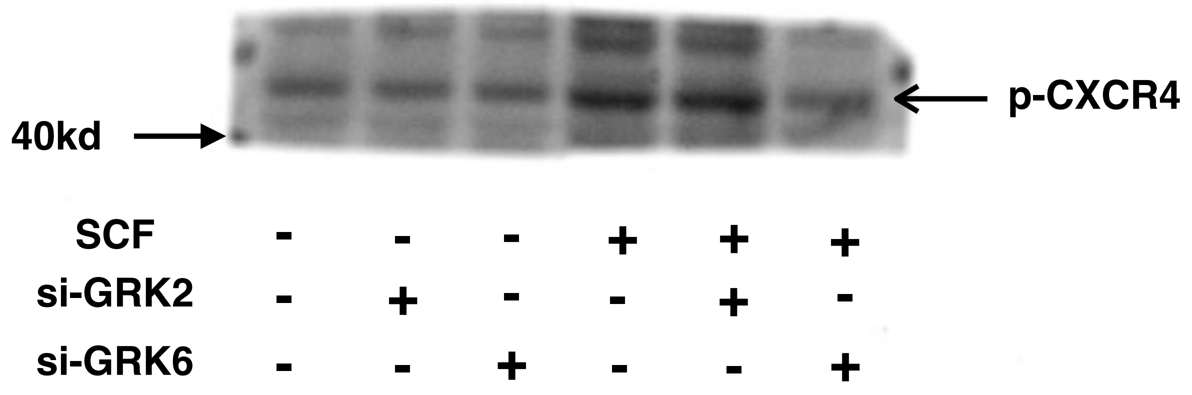

Figure. 6A p-CXCR4 of Knockdown of GRK2 and GRK6

Experiment 3 (Repeated)

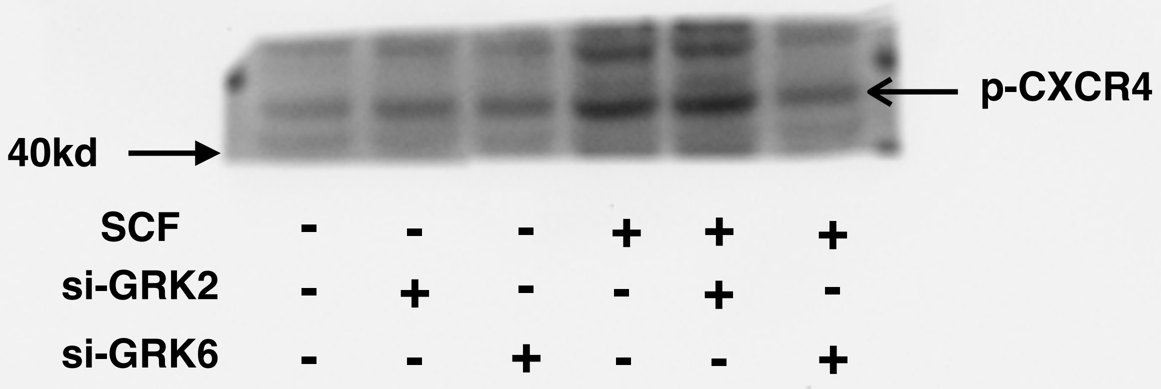

Figure. 6A CXCR4 of Knockdown of GRK2 and GRK6

Experiment 1 (Representative)

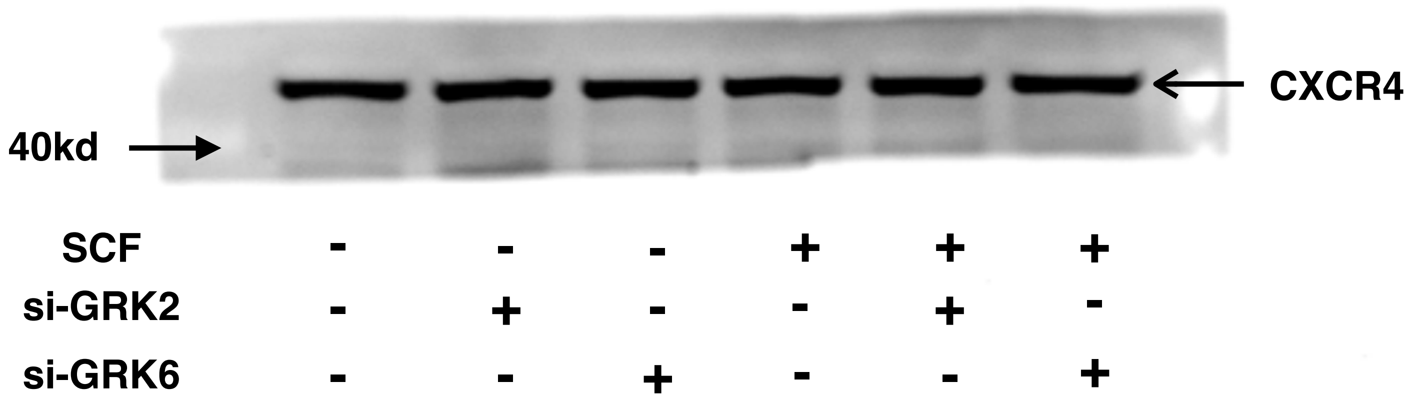

Figure. 6A CXCR4 of Knockdown of GRK2 and GRK6

Experiment 2 (Repeated)

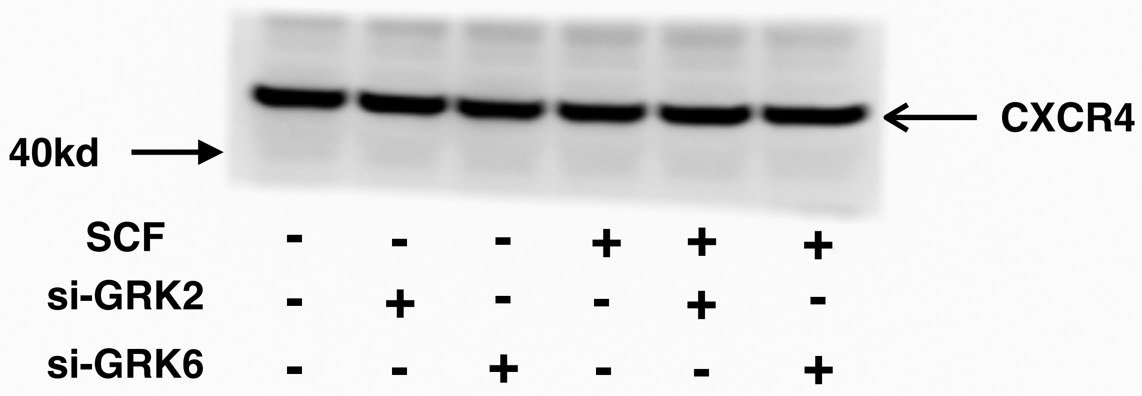

Figure. 6A CXCR4 of Knockdown of GRK2 and GRK6

Experiment 3 (Repeated)

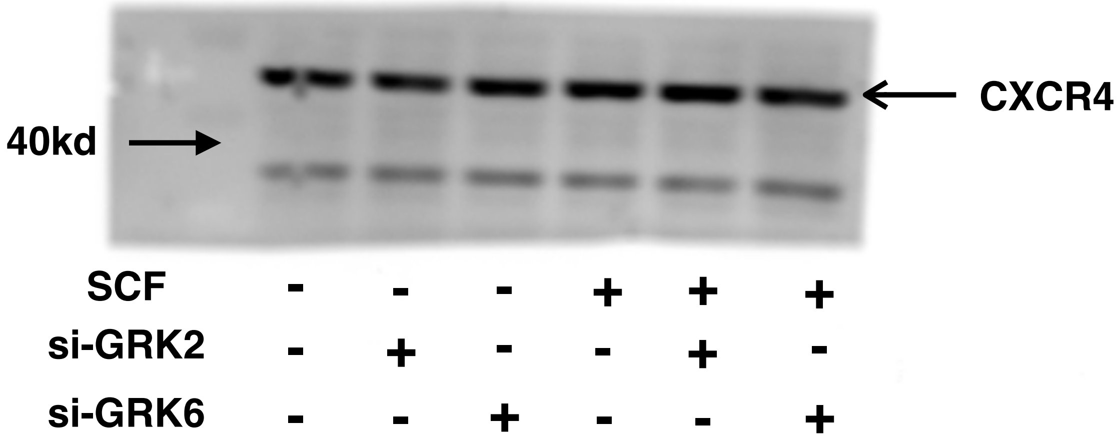

Figure. 6A p-ERK1/2 of Knockdown of GRK2 and GRK6

Experiment 1 (Representative)

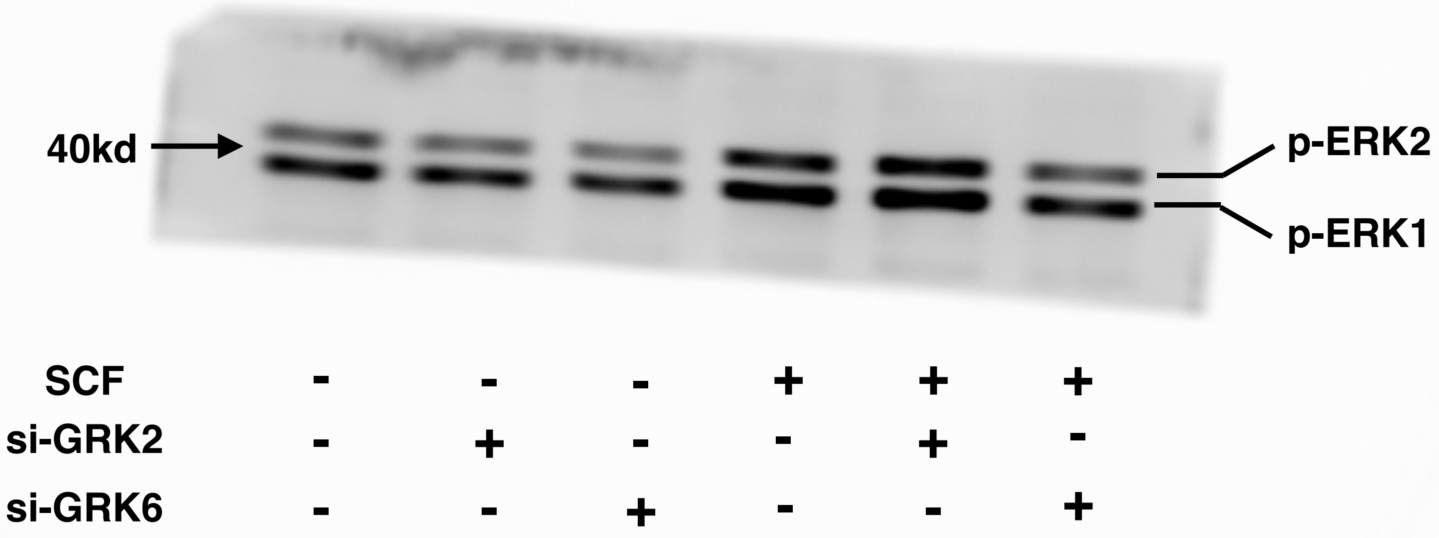

Figure. 6A p-ERK1/2 of Knockdown of GRK2 and GRK6

Experiment 2 (Repeated)

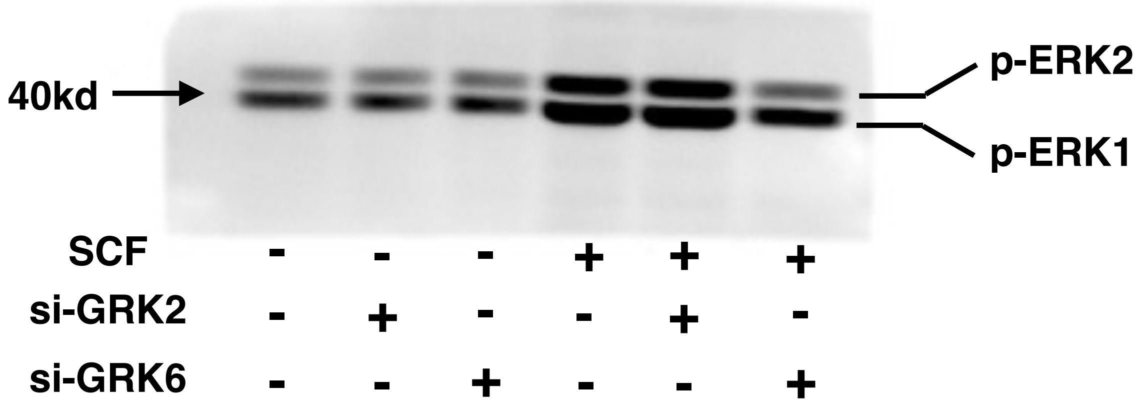

Figure. 6A p-ERK1/2 of Knockdown of GRK2 and GRK6

Experiment 3 (Repeated)

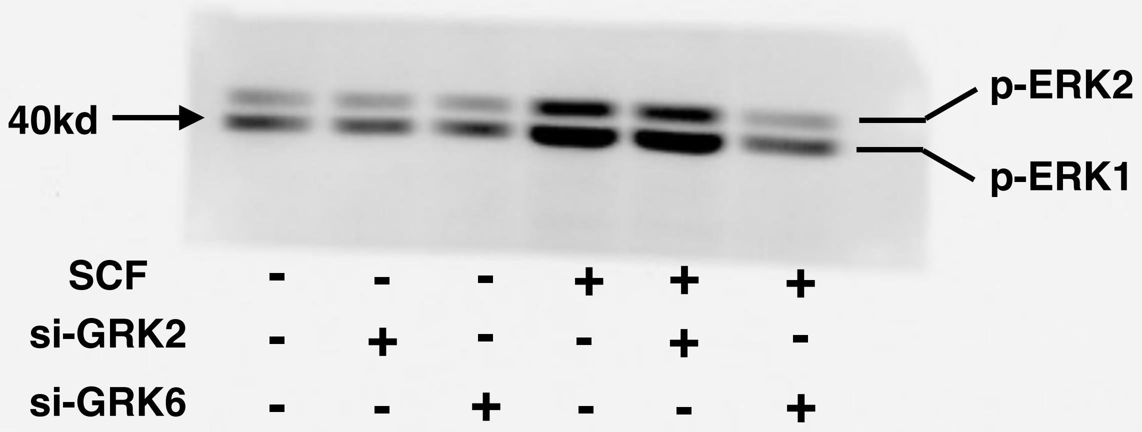

Figure. 6A ERK1/2 of Knockdown of GRK2 and GRK6

Experiment 1 (Representative)

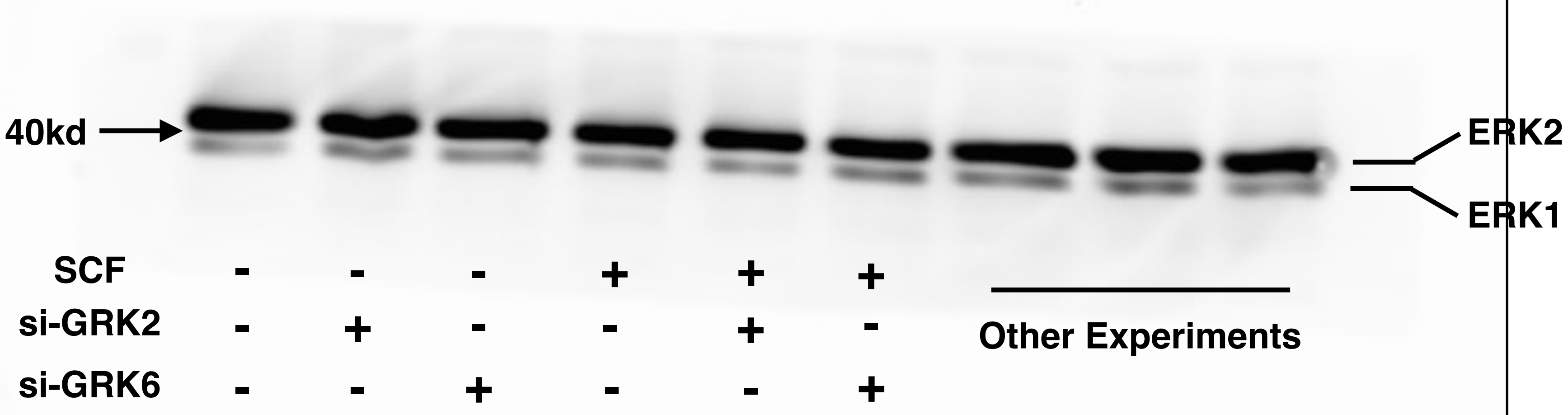

Figure. 6A ERK1/2 of Knockdown of GRK2 and GRK6

Experiment 2 (Repeated)

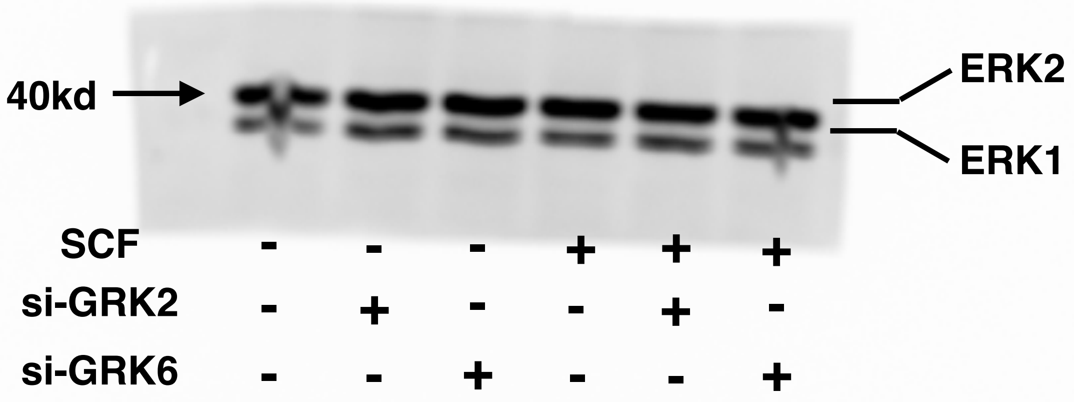

Figure. 6A ERK1/2 of Knockdown of GRK2 and GRK6

Experiment 3 (Repeated)

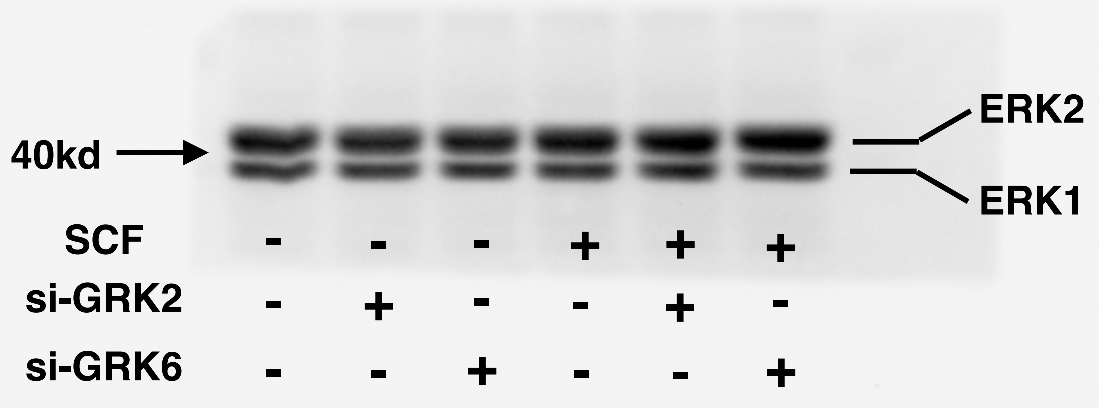

Figure. 6A p-p38 of Knockdown of GRK2 and GRK6

Experiment 1 (Representative)

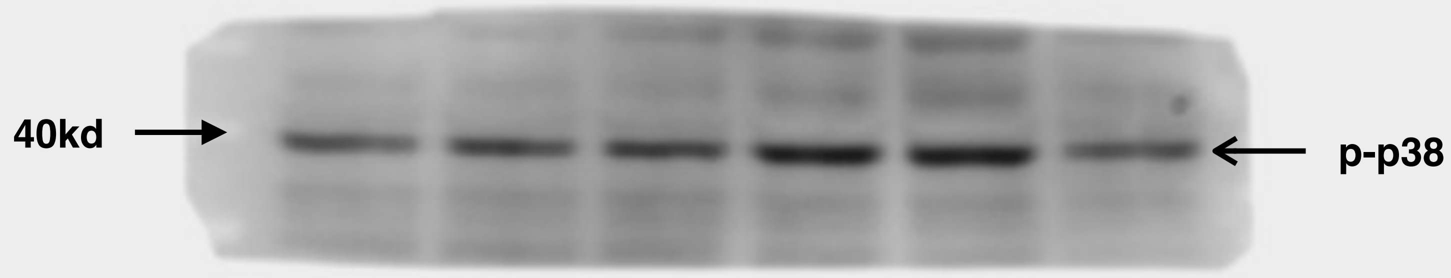

SCF	-	-	-	+	+	+
si-GRK2	-	+	-	-	+	-
si-GRK6	-	-	+	-	-	+

Figure. 6A p-p38 of Knockdown of GRK2 and GRK6

Experiment 2 (Repeated)

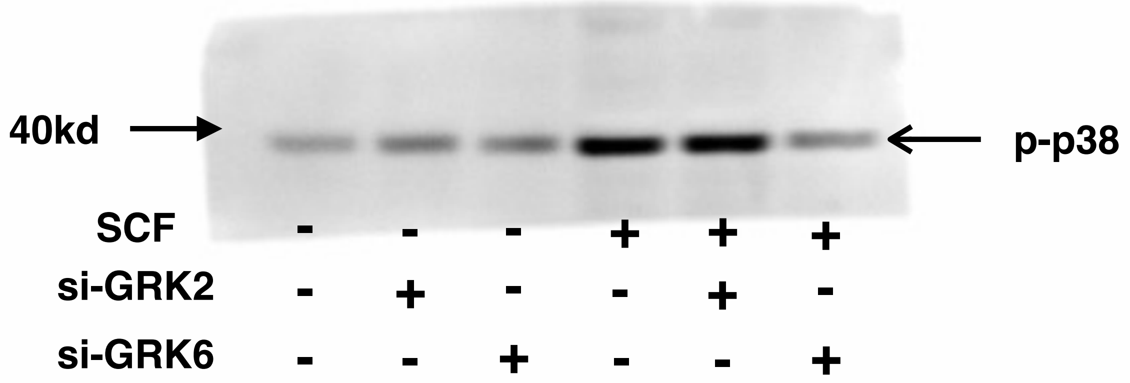

Figure. 6A p-p38 of Knockdown of GRK2 and GRK6

Experiment 3 (Repeated)

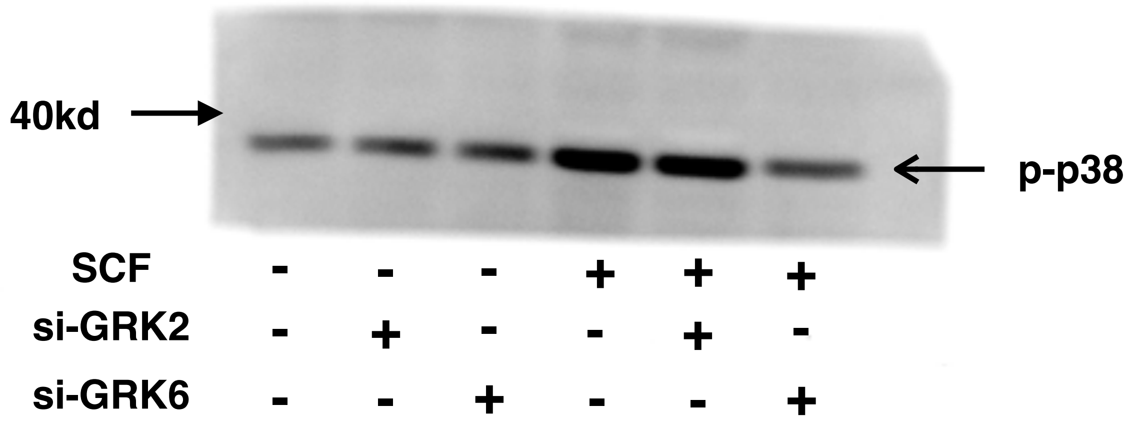

Figure. 6A p38 of Knockdown of GRK2 and GRK6

Experiment 1 (Representative)

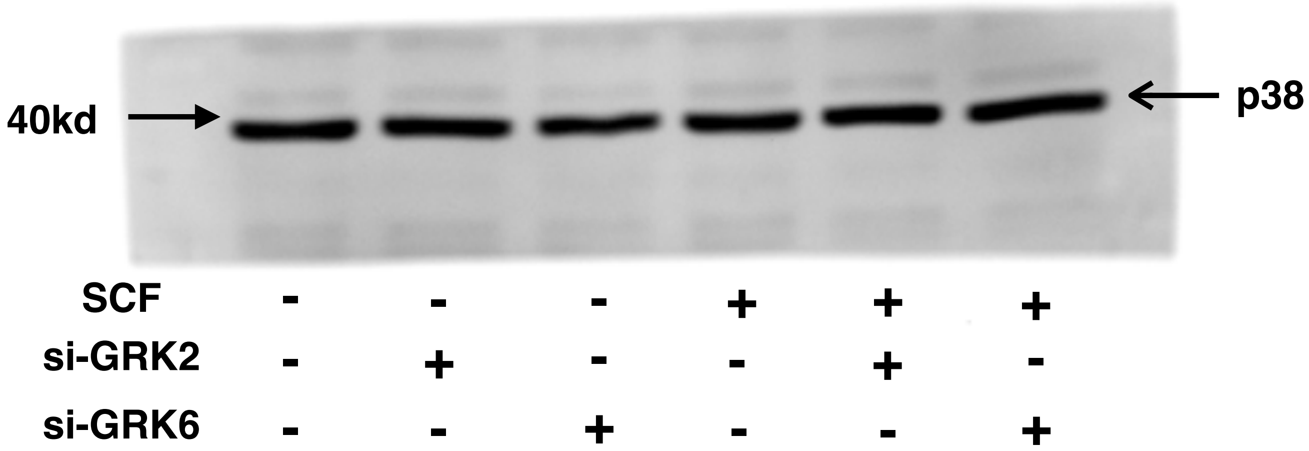

Figure. 6A p38 of Knockdown of GRK2 and GRK6

Experiment 2 (Repeated)

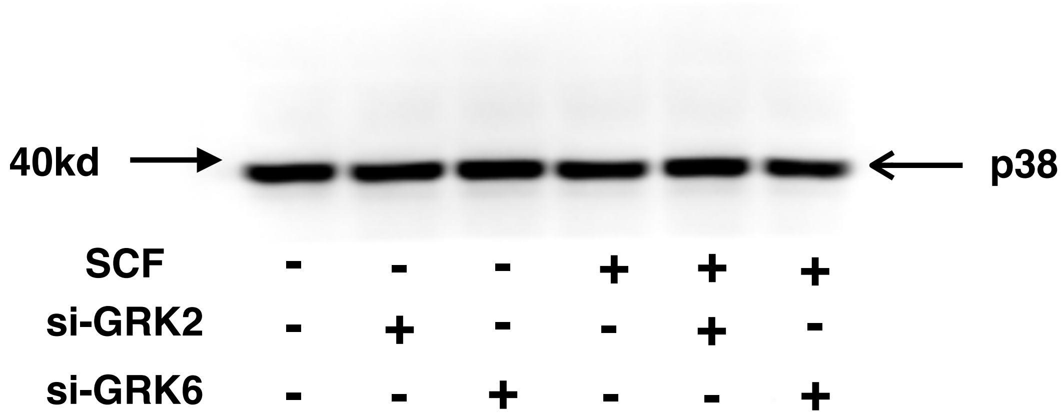

Figure. 6A p38 of Knockdown of GRK2 and GRK6

Experiment 3 (Repeated)

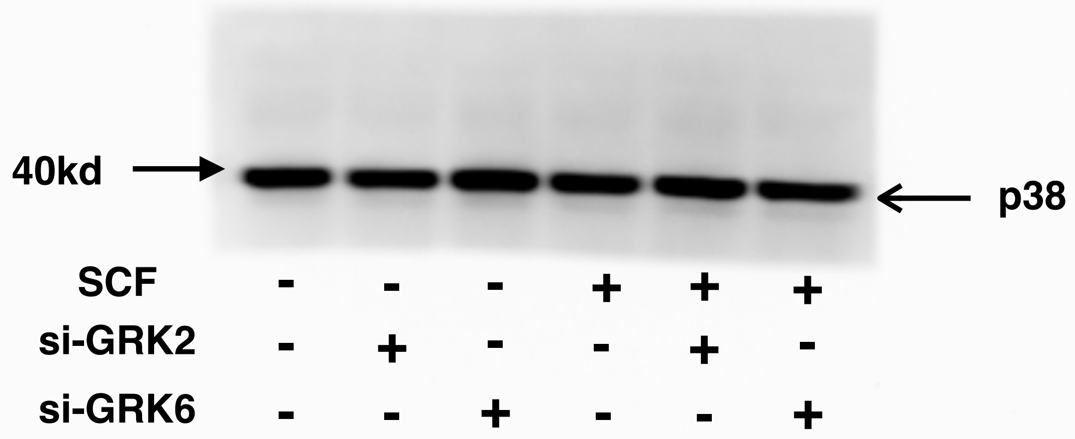

Figure. 6A GRK2 Experiment 1 (Representative)

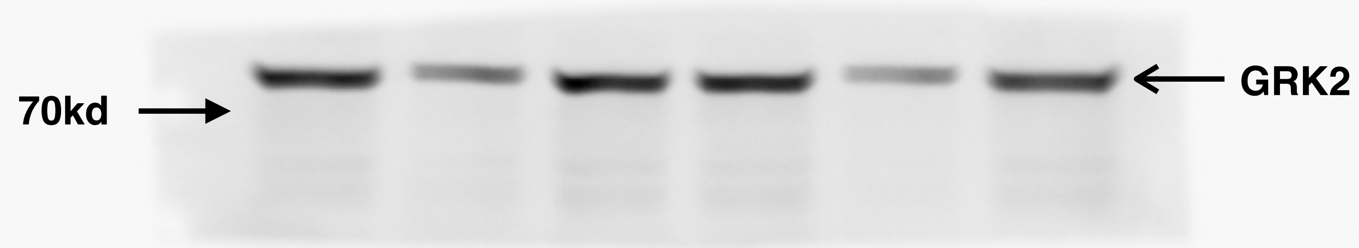

SCF	-	-	-	+	+	+
si-GRK2	-	+	-	-	+	-
si-GRK6	-	-	+	-	-	+

Figure. 6A GRK2 **Experiment 2 (Repeated)**

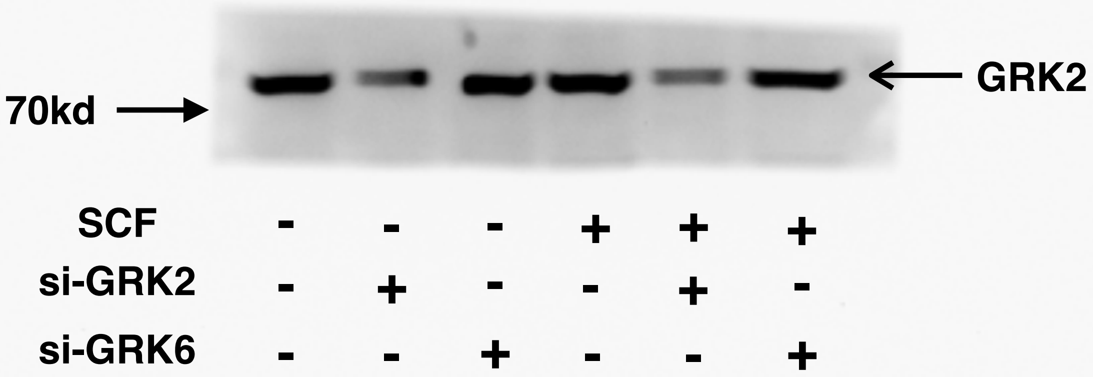

Figure. 6A GRK2 **Experiment 3 (Repeated)**

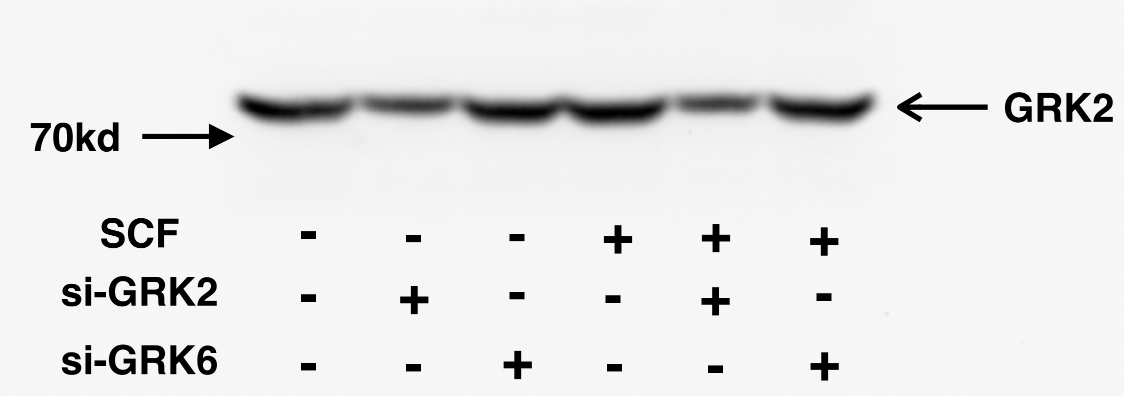

Figure. 6A GRK6 Experiment 1 (Representative)

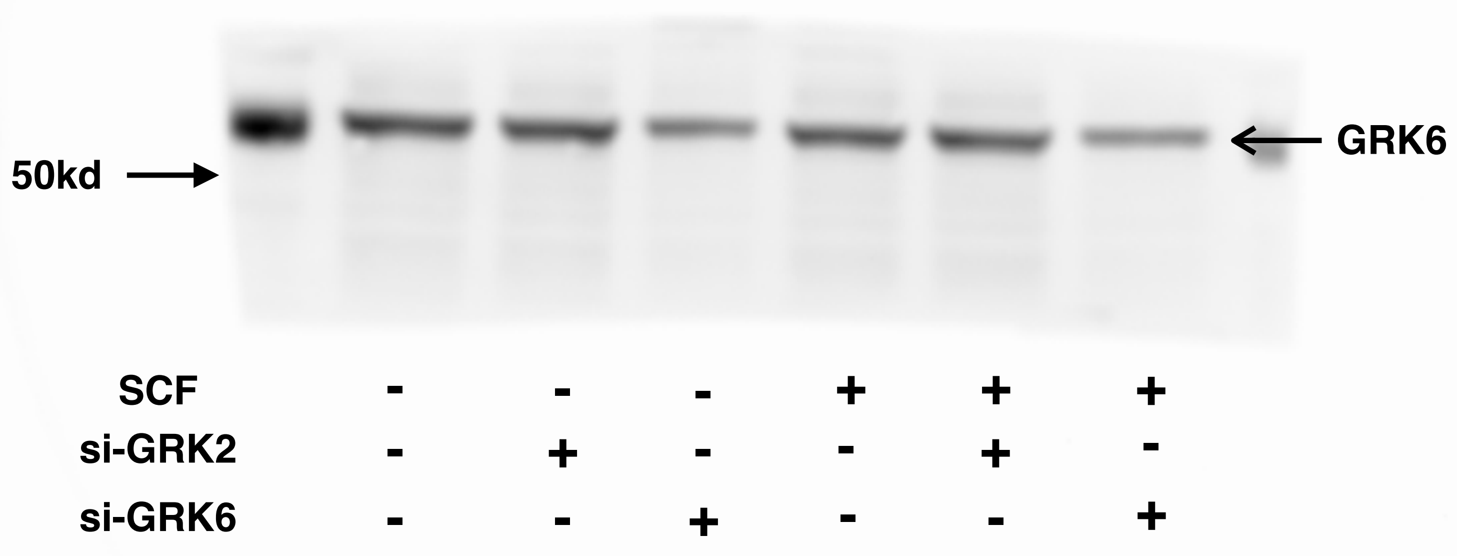

Figure. 6A GRK6 **Experiment 2 (Repeated)**

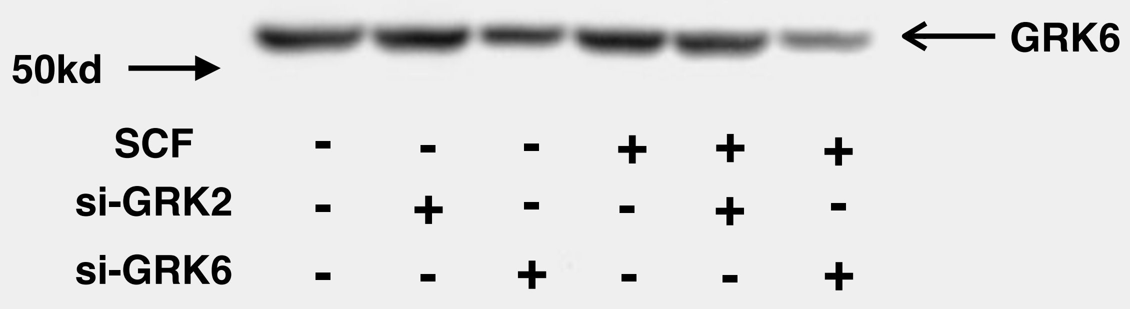

Figure. 6A GRK6 Experiment 3 (Repeated)

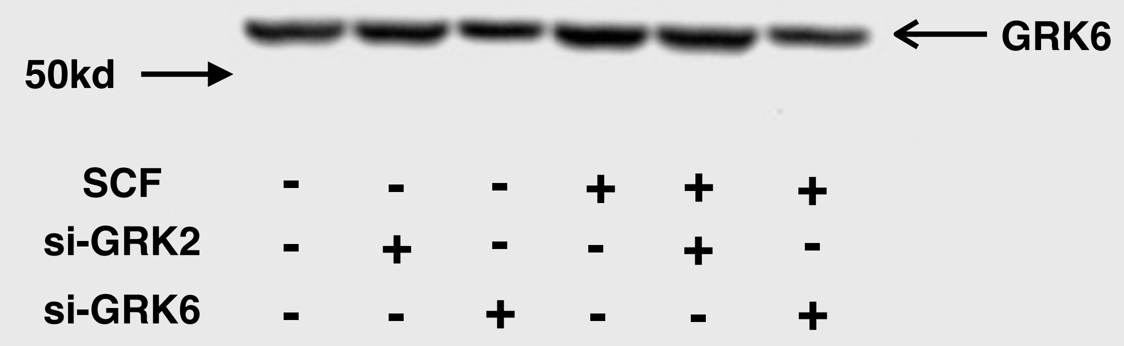

Figure. 6A β-actin of Knockdown of GRK2 and GRK6

Experiment 1 (Representative)

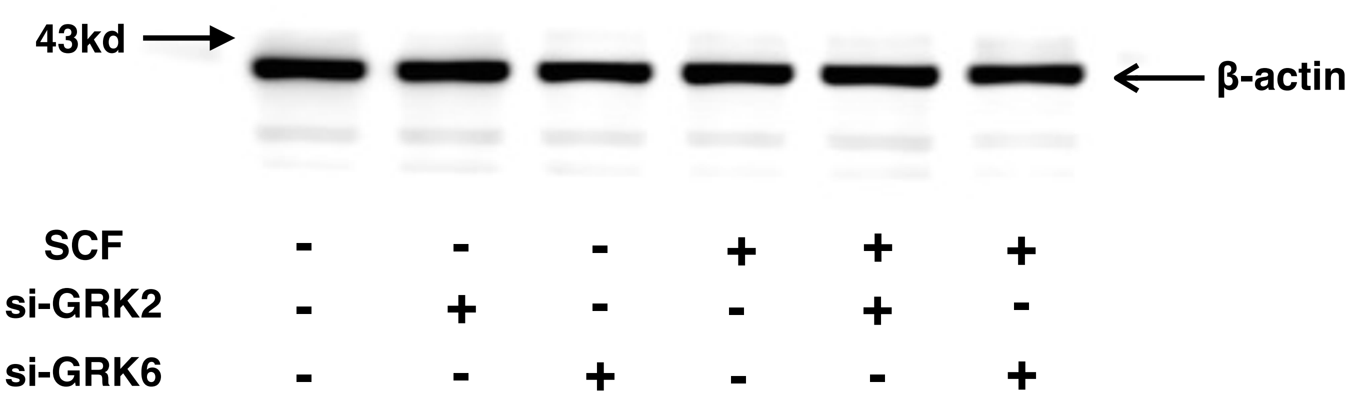

Figure. 6A β-actin of Knockdown of GRK2 and GRK6

Experiment 2 (Repeated)

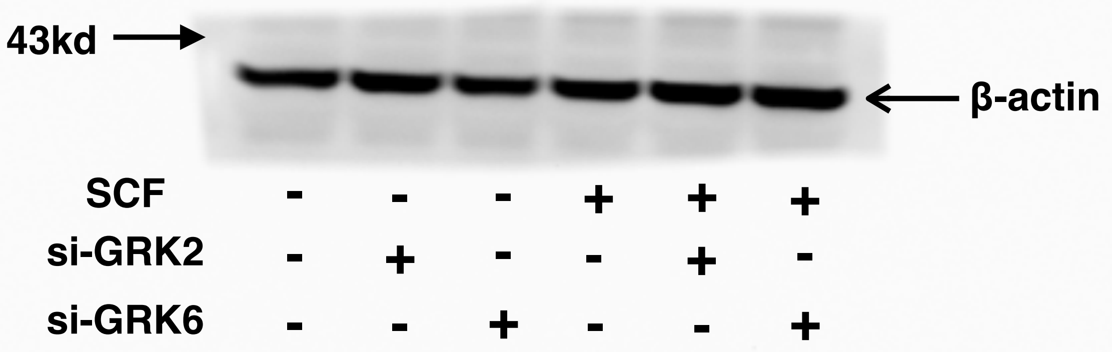

Figure. 6A β-actin of Knockdown of GRK2 and GRK6

Experiment 3 (Repeated)

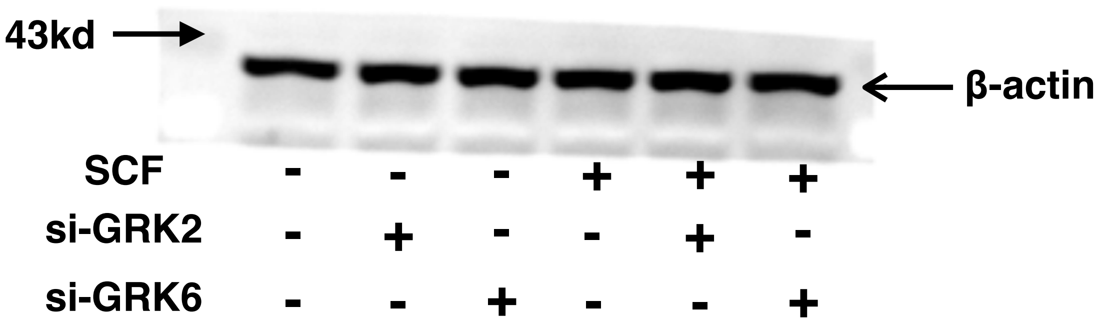

Figure. 6C p-CXCR4 **Experiment 1 (Representative)**

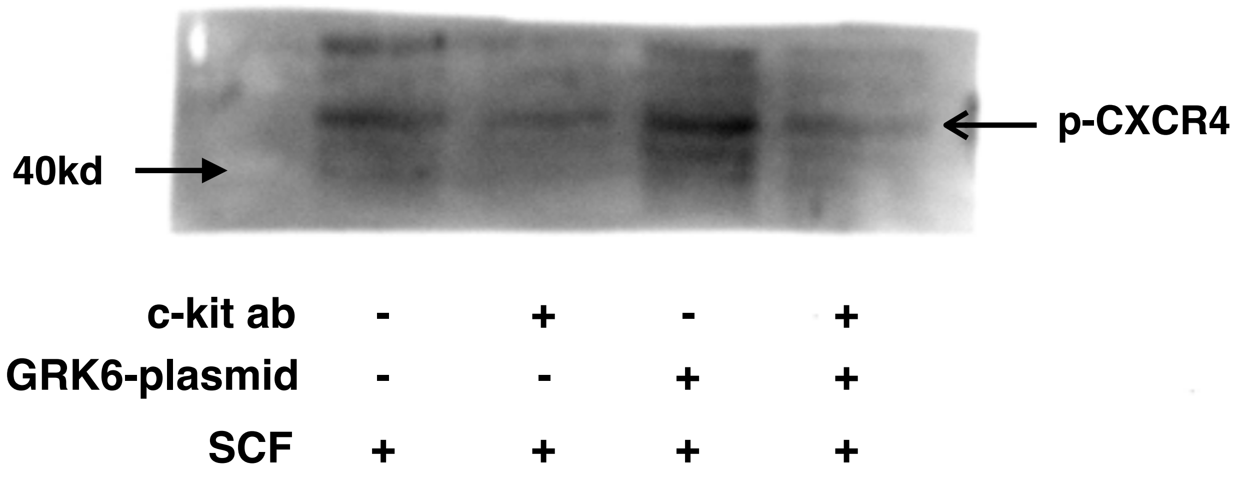

Figure. 6C p-CXCR4 **Experiment 2 (Repeated)**

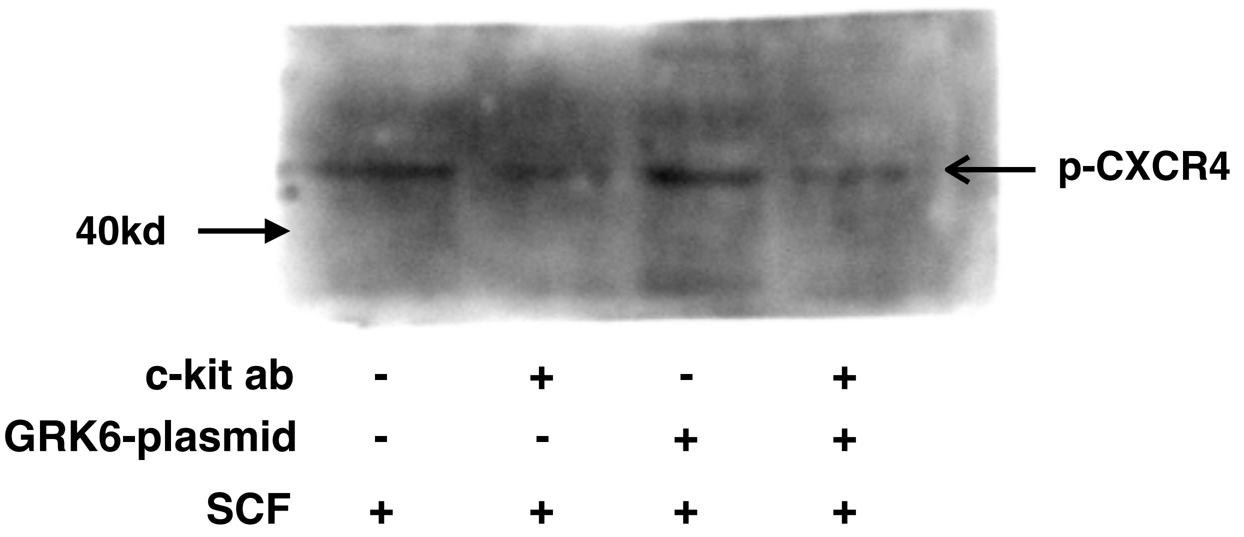

Figure. 6C p-CXCR4 **Experiment 3 (Repeated)**

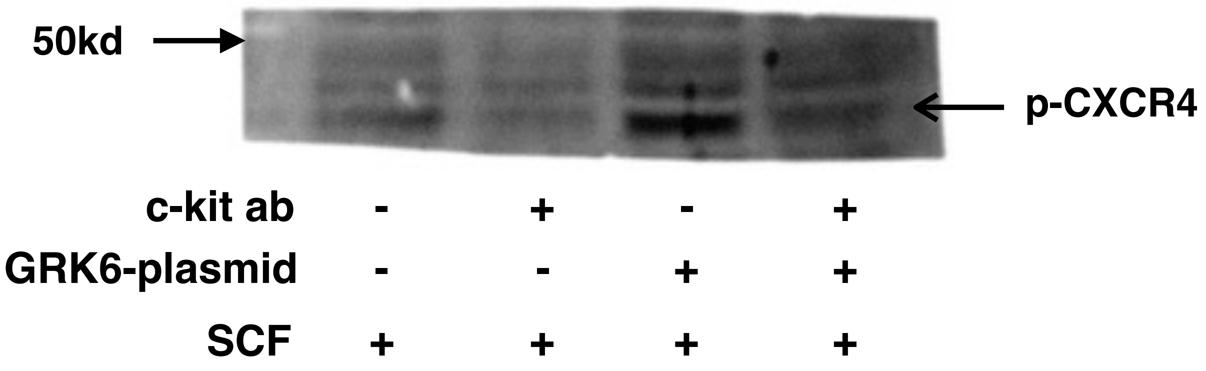

Figure. 6C CXCR4 **Experiment 1 (Representative)**

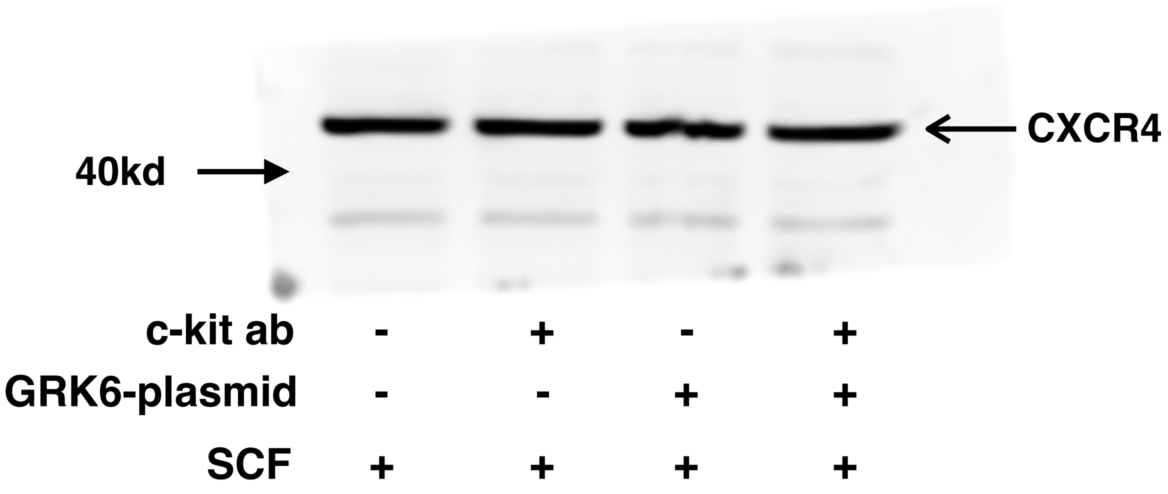

Figure. 6C CXCR4 **Experiment 2 (Repeated)**

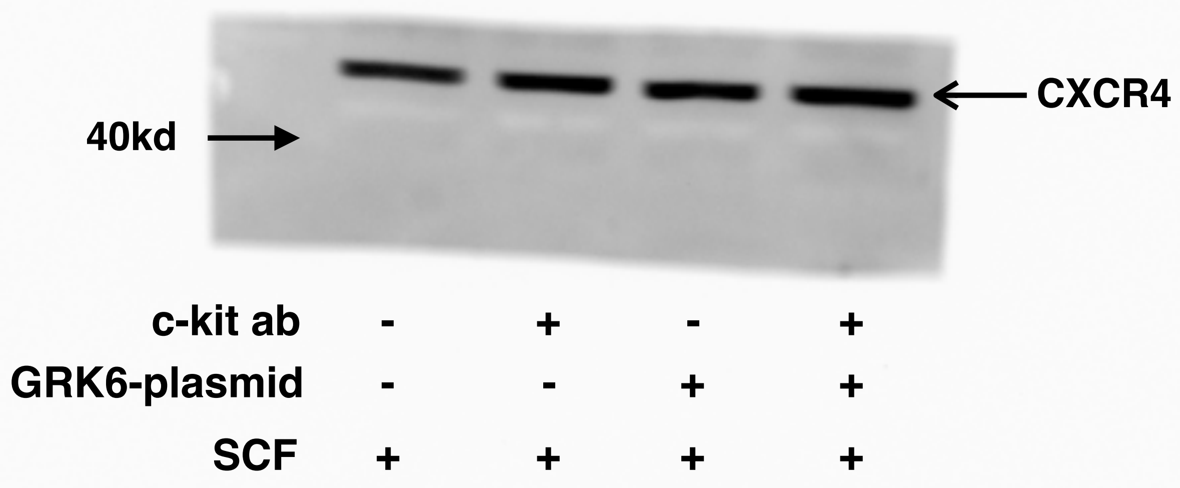

Figure. 6C CXCR4 **Experiment 3 (Repeated)**

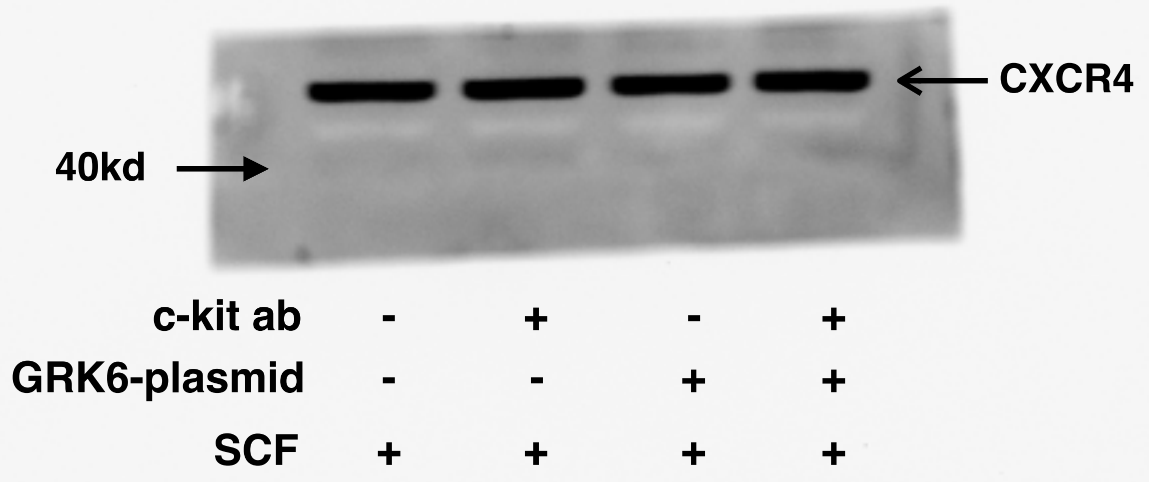

Figure. 6C p-ERK1/2   Experiment 1 (Representative)

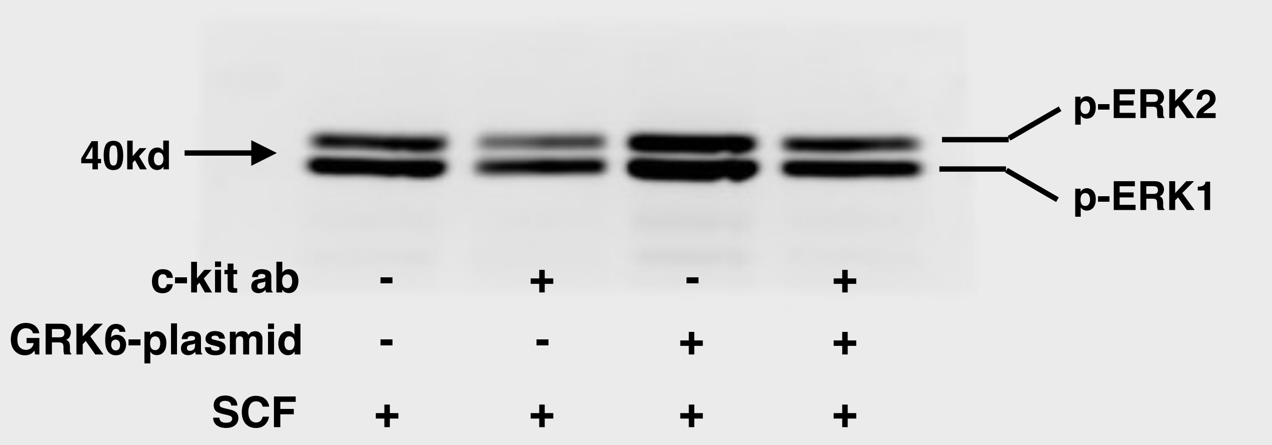

Figure. 6C p-ERK1/2 Experiment 2 (Repeated)

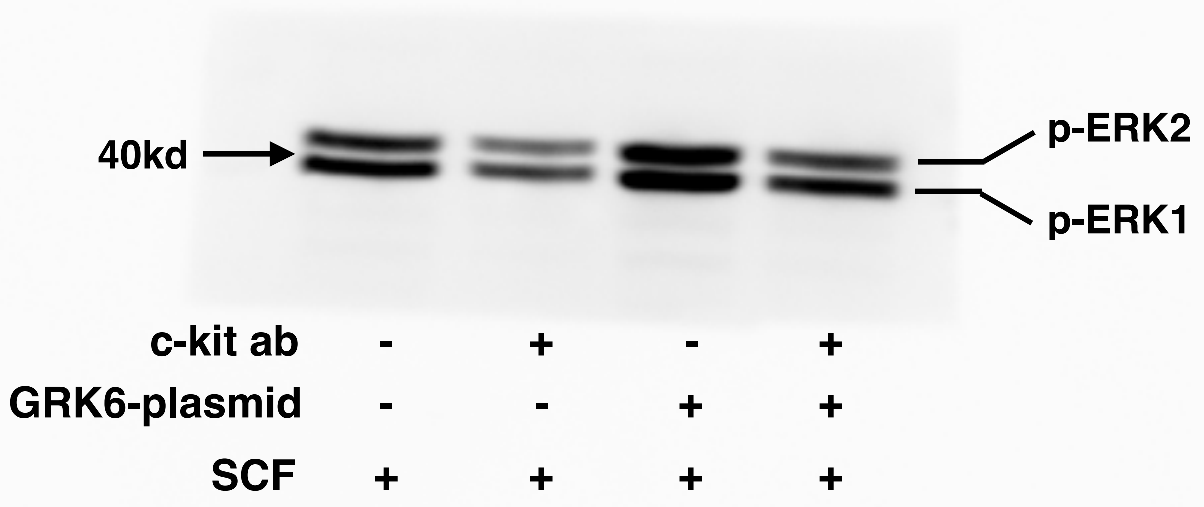

Figure. 6C p-ERK1/2 Experiment 3 (Repeated)

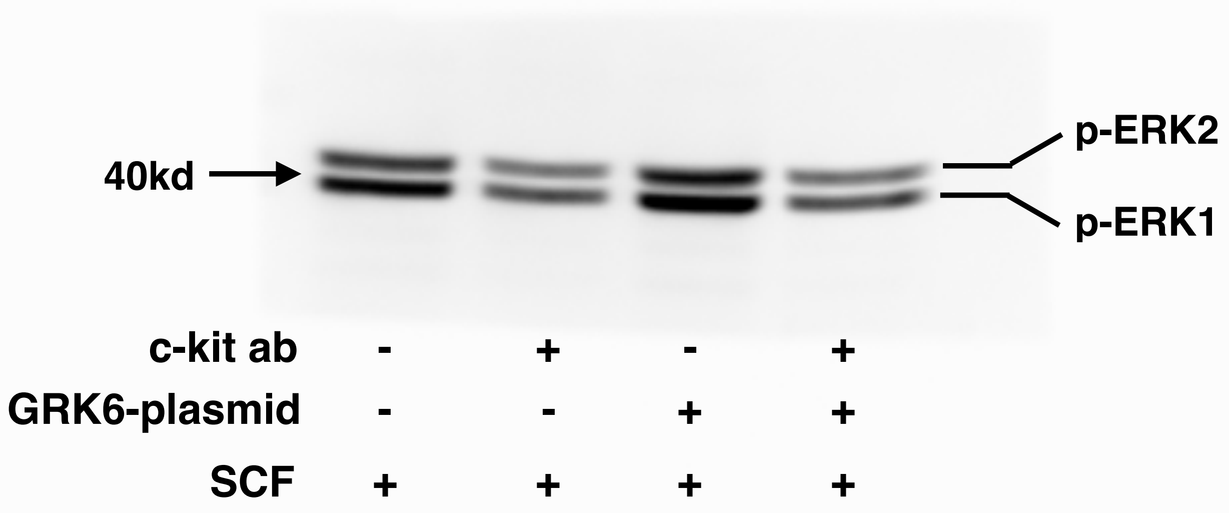

Figure. 6C ERK1/2 Experiment 1 (Representative)

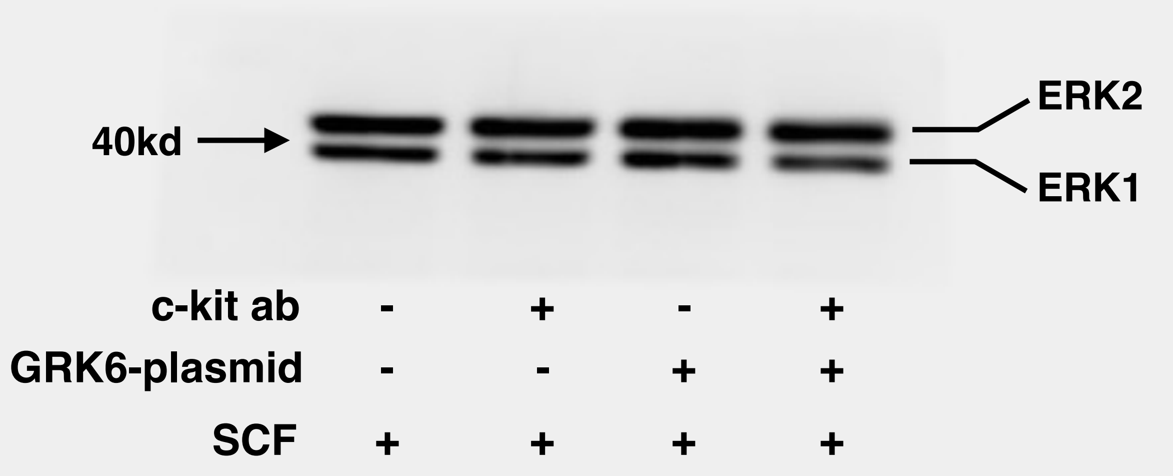

Figure. 6C ERK1/2

Experiment 2 (Repeated)

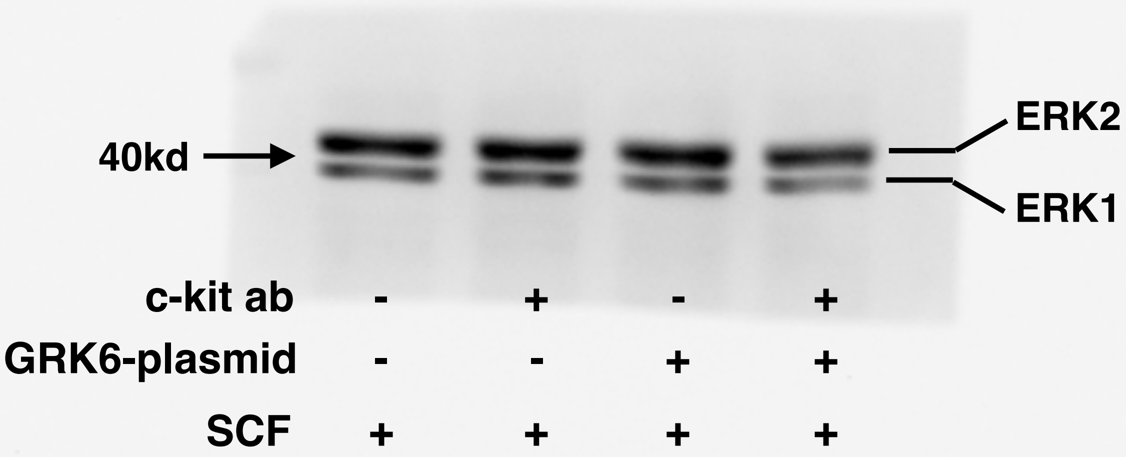

Figure. 6C ERK1/2 **Experiment 3 (Repeated)**

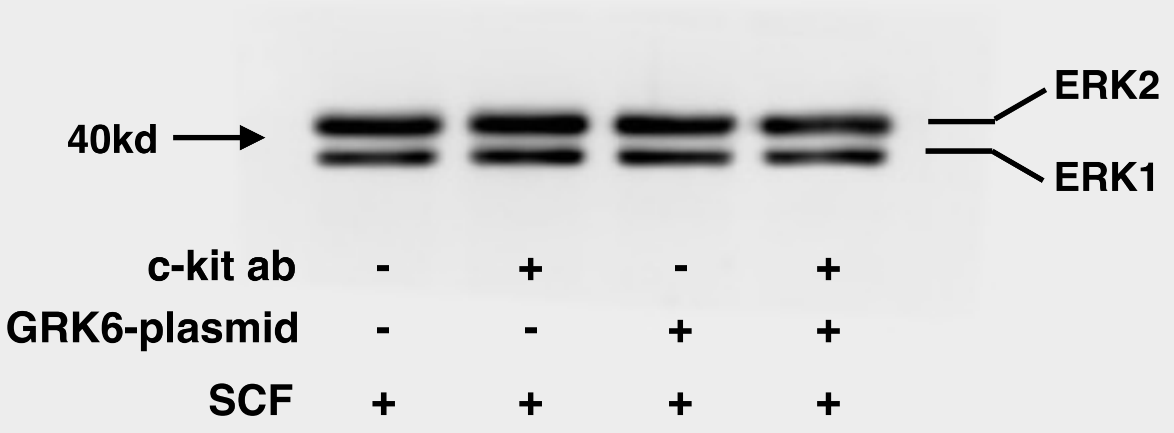

Figure. 6C p-p38 **Experiment 1 (Representative)**

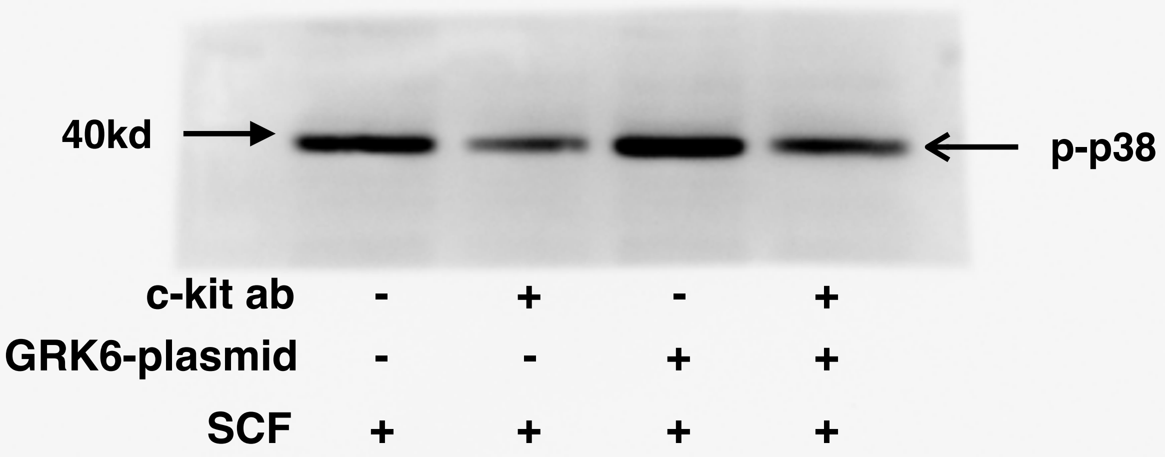

Figure. 6C p-p38 Experiment 2 (Repeated)

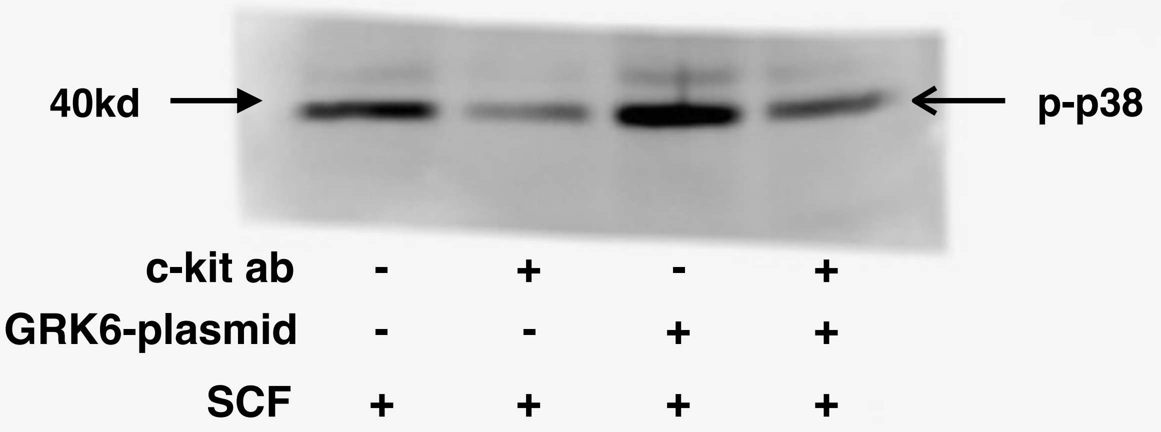

Figure. 6C p-p38 Experiment 3 (Repeated)

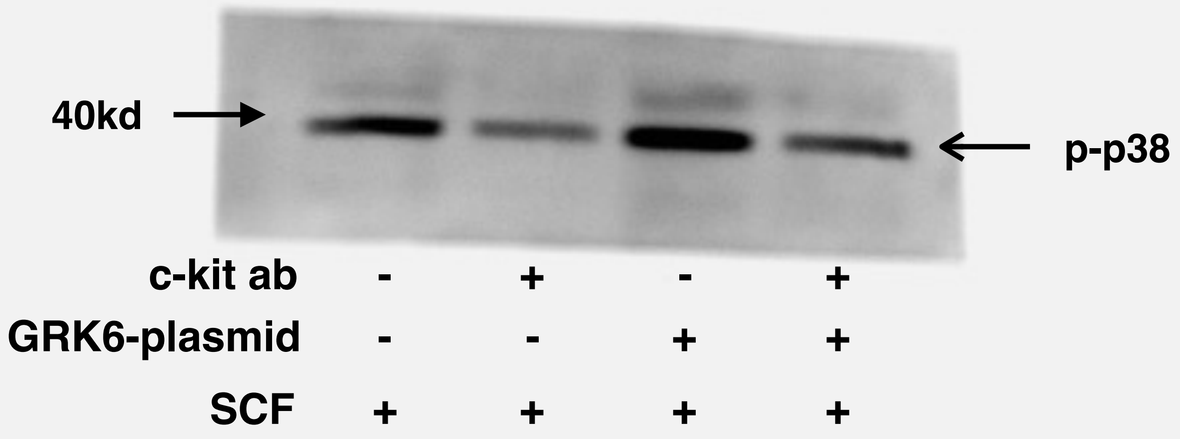

Figure. 6C p38

Experiment 1 (Representative)

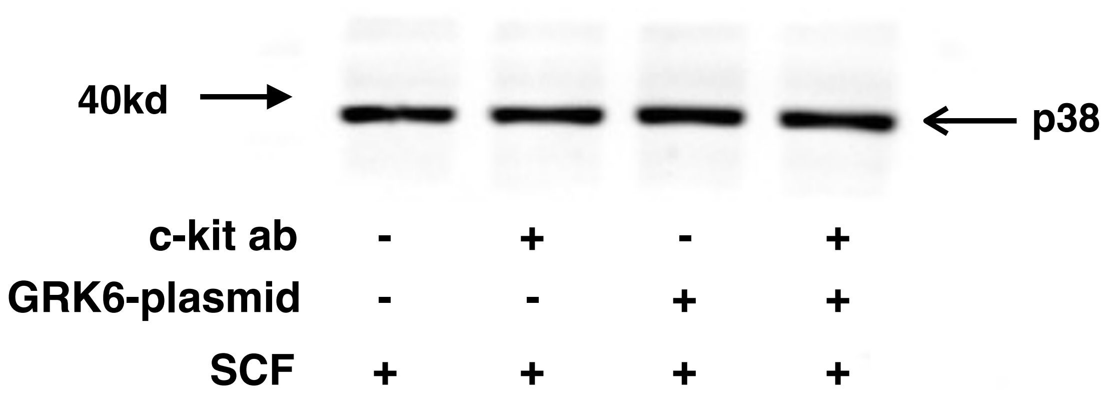

Figure. 6C p38 **Experiment 2 (Repeated)**

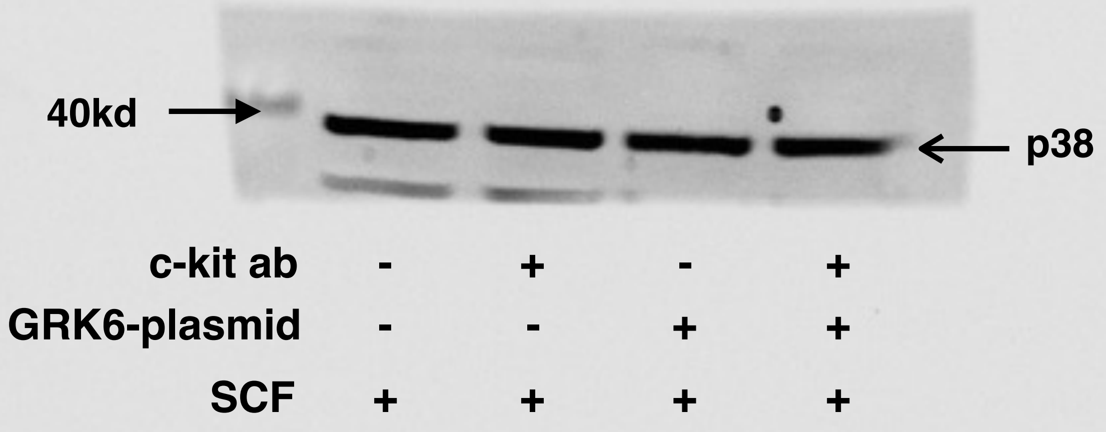

Figure. 6C p38

Experiment 3 (Repeated)

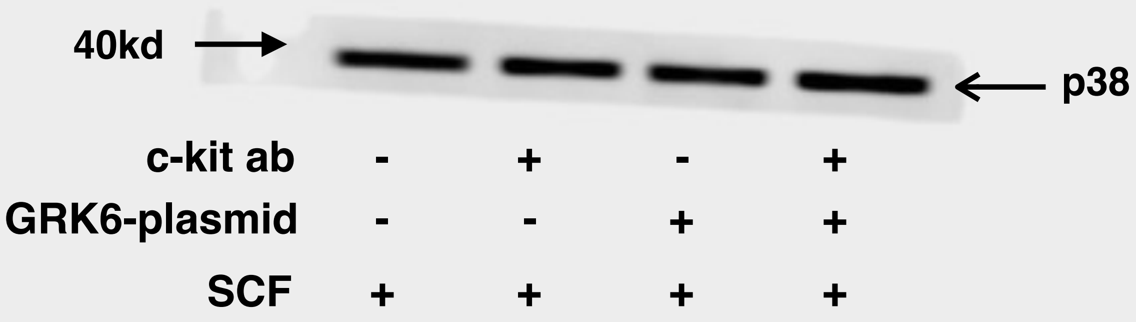

Figure. 6C GRK6 **Experiment 1 (Representative)**

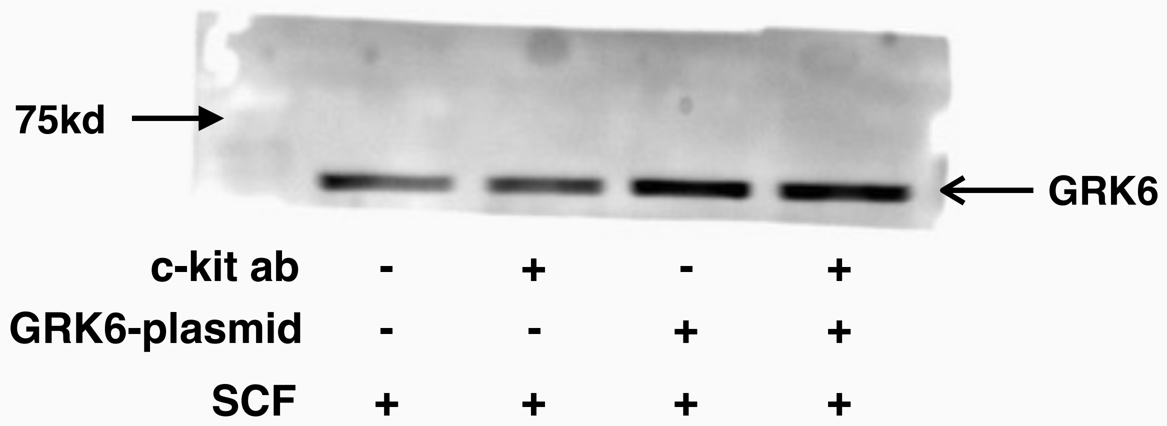

Figure. 6C GRK6 Experiment 2 (Repeated)

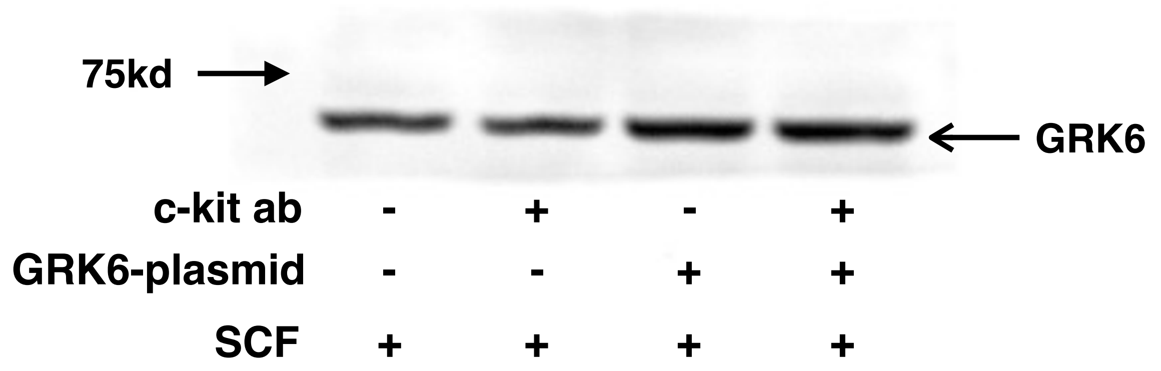

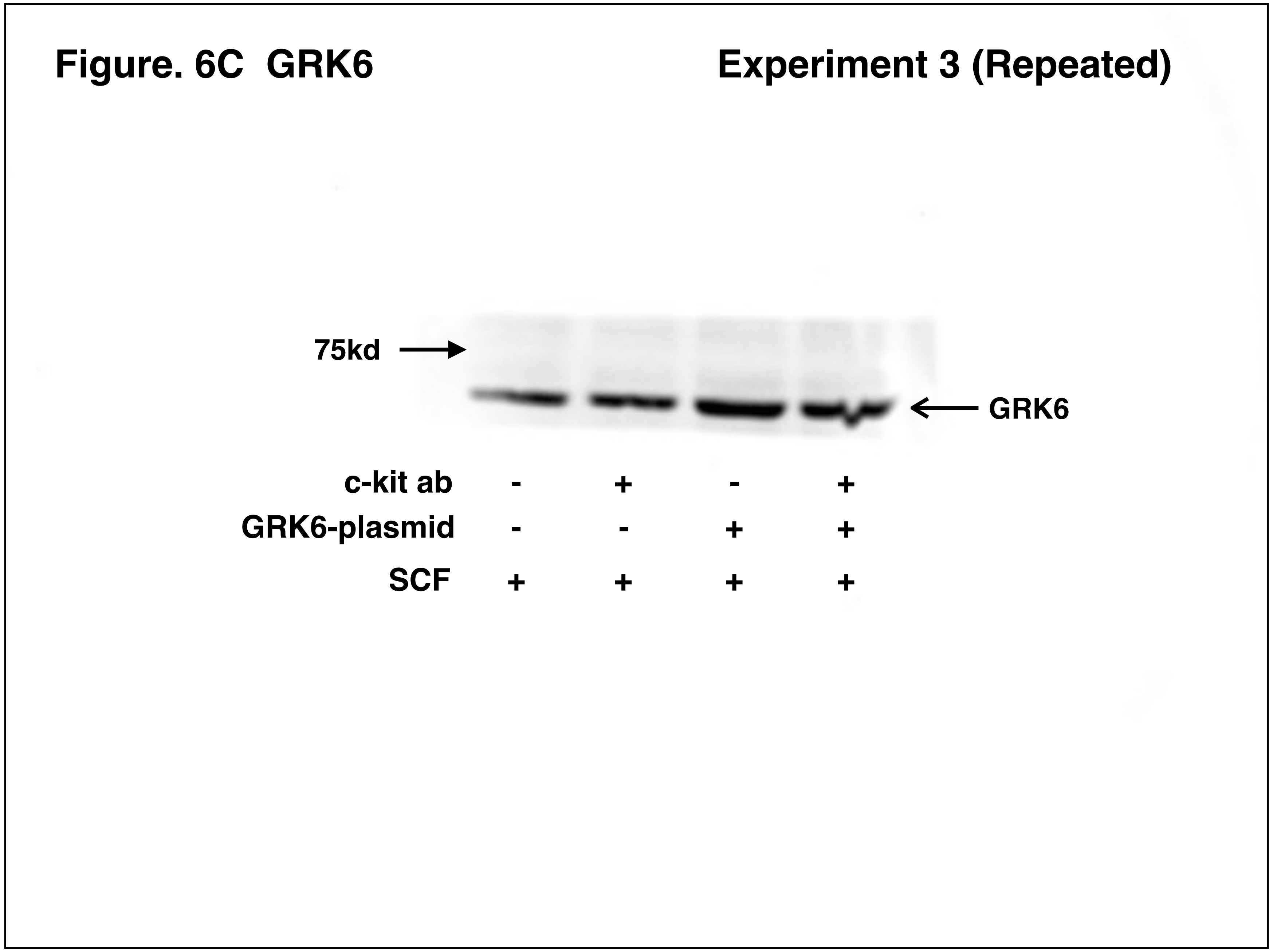
Figure. 6C GRK6
Experiment 3 (Repeated)
75kd
GRK6
c-kit ab - + - +
GRK6-plasmid - - + +
SCF + + + +

Figure. 6C β-actin **Experiment 1 (Representative)**

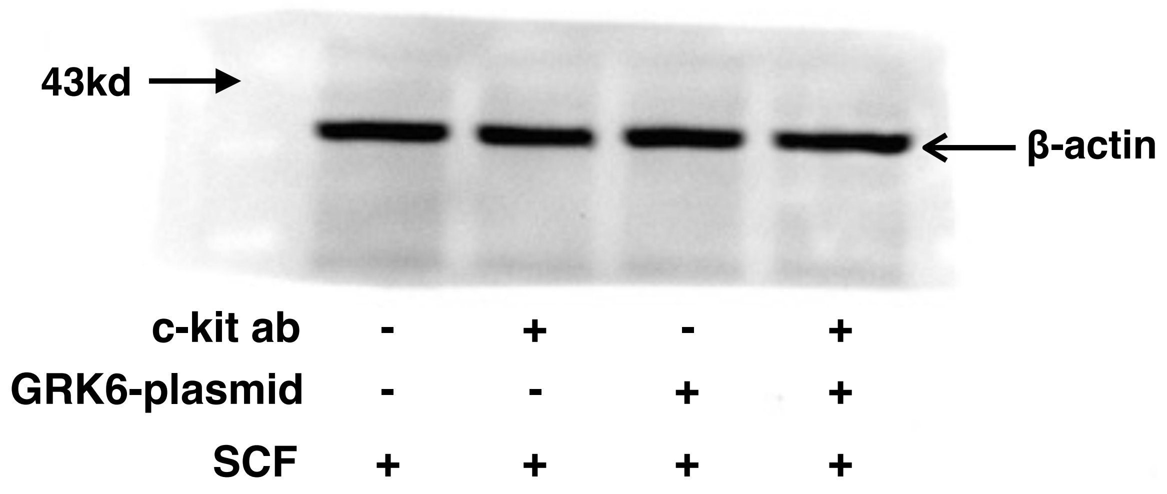

Figure. 6C β-actin **Experiment 2 (Repeated)**

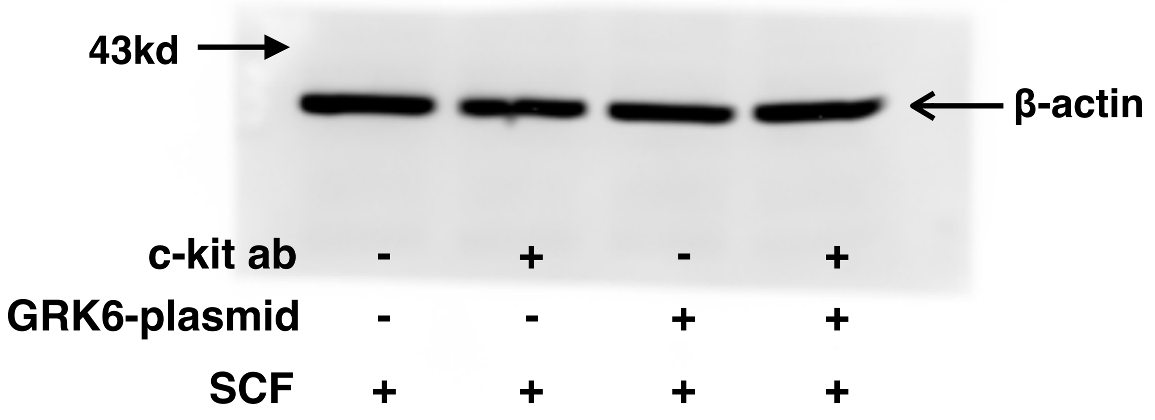

Figure. 6C β-actin

Experiment 3 (Repeated)

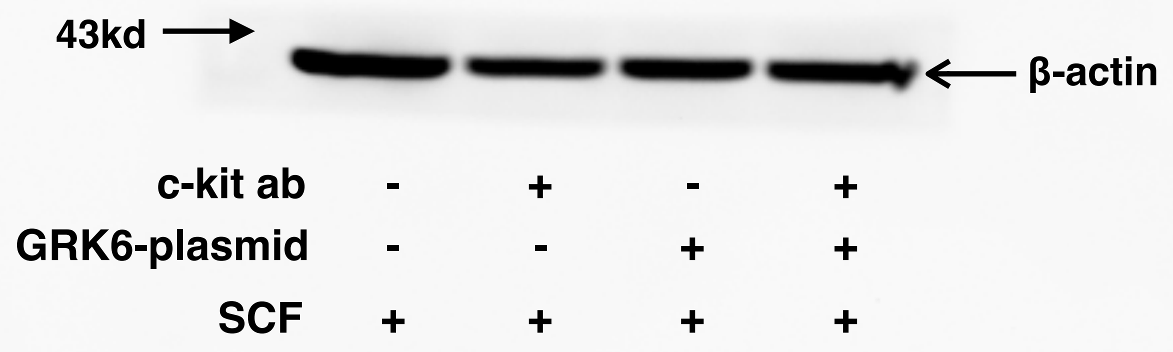

Supplement: Supplementary Figures [file srep26812-s2.pdf]
